# Concise Total Syntheses of the 6–7–5 Hamigeran Natural Products

**DOI:** 10.1021/jacs.3c06031

**Published:** 2023-08-21

**Authors:** Baiyang Jiang, Mingji Dai

**Affiliations:** † Department of Chemistry and Center for Cancer Research, Purdue University, West Lafayette, Indiana 47907, United States; ‡ Department of Chemistry and Department of Pharmacology and Chemical Biology, Emory University, Atlanta, Georgia 30322, United States

## Abstract

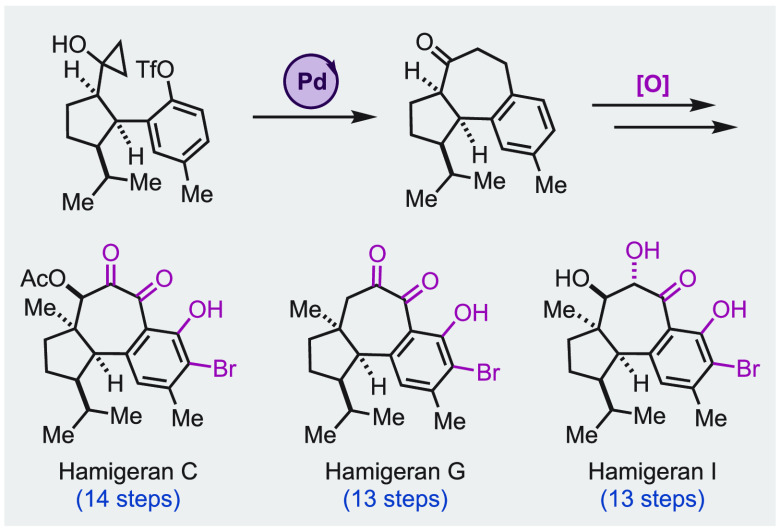

Herein, we report the total syntheses of four hamigeran natural products featuring a 6–7–5 tricyclic carbon skeleton. We utilized a palladium-catalyzed intramolecular cyclopropanol ring opening cross-coupling to build the central seven-membered ring and a series of oxidations including a challenging aromatic C–H oxidation to introduce the peripheral functionalities. This approach enabled us to achieve the first total syntheses of hamigeran C (14 steps), debromohamigeran I (12 steps), and hamigeran I (13 steps). Our synthesis also resulted in hamigeran G in 13 steps, which is significantly shorter than the previously reported one (24 steps, longest linear sequence).

The hamigeran natural products are a family of diterpenoids with diverse chemical structures and remarkable biological activities. They were first discovered from marine sponge *Hamigera tarangaensis* near the New Zealand coast by Cambie and co-workers in 2000.^1^ So far, over 30 family members have been isolated and characterized.^2^ Structurally, most of them feature a characteristic 6–6–5 (hamigerane, **1**, Figure 1A) or 6–7–5 (isohamigerane, **2**–**8**) tricyclic carbon skeleton. The hamigerans have exhibited a broad range of biological activities. For example, hamigeran B (**1**) showed antiviral activity against herpes and polio viruses.^1^ Hamigerans C (**2**), D (**3**), G (**5**), and M (**8**) were identified as promising anticancer leads with moderate cytotoxicity (2.5–37.2 μM IC_50_ values) against leukemia cells.^2^ Biosynthetically, the hamigerans are synthesized from geranyl geranyl pyrophosphate (GGPP, **9**, Figure 1B) via a sequence of C–H oxidations and cyclizations to generate intermediate **12**.^2b^ Subsequent enzymatic removal of the pyrophosphate (OPP) from **12** would trigger a 1,2-hydride shift to the hamigeranes (pathway a) or 1,2-alkyl shift and ring expansion (pathway b, cf. **12** → **13**) to the isohamigeranes.

**Figure 1:**
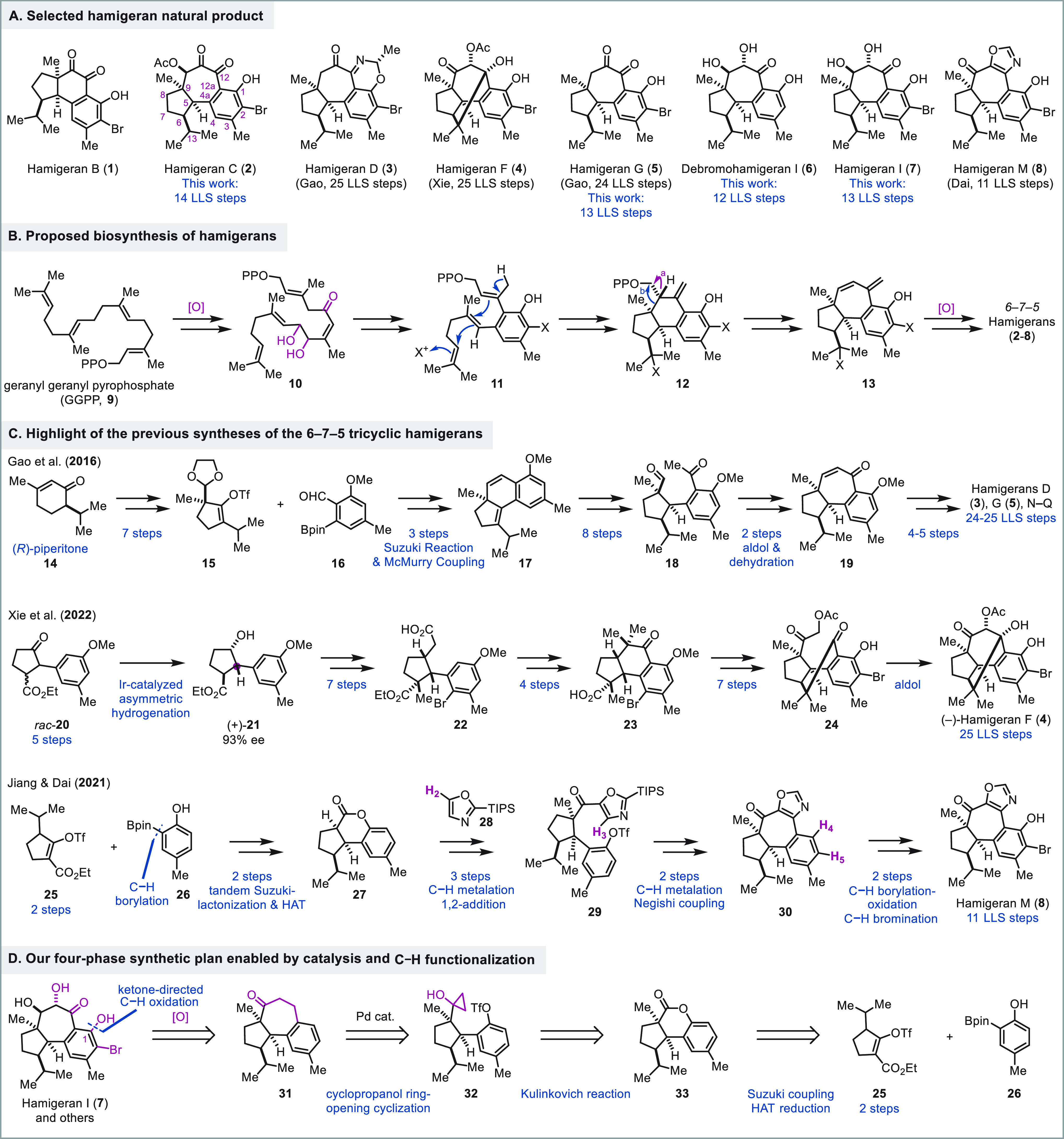
Hamigerans, plausible biosynthesis, prior synthesis, and retrosynthetic analysis.

Previously, most of the hamigeran total synthesis efforts focused on the members with a 6–6–5 tricyclic carbon skeleton,^3^ and many elegant total syntheses have been reported.^4^ Reports on the total syntheses of the more challenging hamigerans with a 6–7–5 tricyclic carbon framework are sporadic (Figure 1C).^5^ Represented by hamigeran C (**2**), these hamigerans have a polysubstituted central seven-membered ring with multiple oxygenated carbons and contiguous stereocenters. The seven-membered ring is further fused with a polyfunctionalized aromatic ring and a cyclopentane ring. These unique structural features make their synthesis difficult. In 2016, Gao and co-workers reported the first total syntheses of hamigeran G (**5**) and its nitrogen-containing congeners hamigerans D (**3**) and N–Q.^5a^ Their synthesis started from (*R*)-piperitone (**14**). A combination of Suzuki coupling between **15** and **16** and intramolecular McMurry coupling afforded **17** with a 6–6–5 tricyclic skeleton. After expanding the six-membered ring to the desired seven-membered ring via a lengthy sequence, they achieved the total syntheses of hamigeran G (**5**) in 24 longest linear sequence (LLS) steps and hamigerans D (**3**) and N–Q in 25 LLS steps. In 2022, Xie and co-workers reported their total synthesis of hamigeran F (**4**) with a unique C–C bond between C12 and C13.^5c^ Their synthesis features an Ir-catalyzed asymmetric hydrogenation of racemic **20** to **21** in high yield and enantioselectivity (93% ee). After advancing **21** to **24**, they used an intramolecular aldol reaction to close the seven-membered ring and prepared hamigeran F (**4**) in 25 steps (LLS). In 2018, we reported an approach featuring a benzyne-β-ketoester annulative ring expansion to form the seven-membered ring and a Nazarov reaction to form the five-membered ring. Unfortunately, this approach hit a dead end and did not lead to any hamigeran natural products.^6a^ In 2021, we completed the total synthesis of hamigeran M (**8**) with an unusual oxazole fused with the central seven-membered ring.^6b^ The oxazole functionality enabled us to utilize two C–H functionalizations to forge the seven-membered ring (**27** + **28** → **29** → **30**) and complete the total synthesis of hamigeran M (**8**) in 11 steps. Additionally, tricyclic lactone **27** was assembled in a convergent manner from two readily available building blocks **25** and **26** via a sequence of Suzuki reaction–lactonization and hydrogen atom transfer (HAT) chemistry to reduce the corresponding tetrasubstituted double bond. Herein, we report our continued efforts in this direction, which resulted in the total syntheses of hamigeran C (**2**) in 14 steps, hamigeran G (**5**) in 13 steps (vs 24 LLS steps from Gao’s synthesis), debromohamigeran I (**6**) in 12 steps, and hamigeran I (**7**) in 13 steps.

Retrosynthetically (Figure 1D), we envisioned **31** as an advanced intermediate from which a series of oxidation would lead to hamigeran I (**7**) and other congeners. To introduce the hydroxyl group on the aromatic ring (C1), we planned a ketone-directed C–H hydroxylation or borylation–oxidation.^7^ For the synthesis of **31**, we designed a Pd-catalyzed intramolecular cyclopropanol ring opening cross-coupling to forge the seven-membered ring. Such a seven-membered ring formation was developed by Cha and co-workers^8^ but has not been used in any total synthesis before. Cyclopropanol **32** would be derived from tricyclic lactone **33** via a Kulinkovich reaction.^9^ Lactone **33** was previously prepared by us from readily available building blocks **25** and **26**.^6b^

Our synthesis commenced with preparing **33** from **25** and **26** via a three-step sequence, namely tandem Suzuki reaction–lactonization, HAT reduction, and α-methylation.^6b^ We then investigated the Kulinkovich reaction to convert **33** to **32** and encountered the first obstacle in our synthesis. We were not able to prepare **32** presumably due to the steric hindrance generated by the all-carbon quaternary center. We thus decided to use **27** without the α-methyl group for the Kulinkovich cyclopropanol synthesis. This less hindered lactone under the Corey modified Kulinkovich conditions (TiCl(O*i*-Pr)_3_ and EtMgBr)^10a^ was converted to the desired product **34** in 66% yield after trapping the resulting phenol with bis(trifluoromethanesulfonyl)aniline (PhNTf_2_). We then explored Cha’s Pd-catalyzed cyclopropanol ring opening cross-coupling to form the central seven-membered ring.^8a^ The choice of base turned out to be critical for its success. Under the original conditions (Pd(OAc)_2_, 1,4-bis(diphenylphosphino)butane (dppb), Cs_2_CO_3_ in CH_3_CN at 80 °C) developed by Cha and co-workers, no ring closing product was observed, but instead the triflate hydrolysis and cyclopropanol ring opening byproducts. Replacing Cs_2_CO_3_ with less basic KHCO_3_ resulted in the desired product **35** in 79% yield at 110 °C. We then decided to introduce the missing α-methyl group, which was problematic for the cyclopropanol synthesis. A thermodynamic enolate formation and methylation was realized by treating **35** with KO*t-*Bu and methyl iodide in a mixture of HMPA and THF at 60 °C.^11^ Our original plan was to use a ketone-directed C–H hydroxylation or borylation–oxidation to install the C1 hydroxyl group, which turned out to be unsuccessful. As shown in Scheme 1B, after a selective and thermodynamic α-methylation, the Sharpless selenoxide elimination was used to synthesize α,β-unsaturated ketone **46**,^12^ which was obtained in 45% yield from **35** with only one purification involved. The ketone was next reduced to an allylic alcohol stereoselectively with DIBAL-H by delivering the hydride from the less hindered convex face. The resulting alkoxide was converted to acetate **47** in the same pot. We next needed to oxidize the double bond in the seven-membered ring of **47** to an α-hydroxy ketone (cf., **48**) in a stereo- and regioselective manner. After a series of explorations, the ketohydroxylation (**47** → **48**) was achieved using the Plietker protocol with RuCl_3_ as catalyst and Oxone as the external oxidant.^13^ The reaction went through a convex face [3 + 2]-cycloaddition of the in situ generated RuO_4_ with **47** to form a five-membered ruthenate intermediate, which further reacted with Oxone to open the ruthenate for a subsequent oxidation to give **48** in 33% yield. In this unoptimized case, the other α-hydroxy ketone isomer and the dihydroxylation product were observed as minor products. Unfortunately, the next ketone-directed C–H functionalization at C1 proved to be extremely difficult. Cu-, Pd-, or Ru-catalyzed or mediated C–H oxygenation did not give any desired product nor did the Rh- or Ir-catalyzed C–H borylation.^7,14^ We also tried to convert the ketone to a stronger directing group such as oximes but were not able to make the latter.^15^

**Scheme 1:**
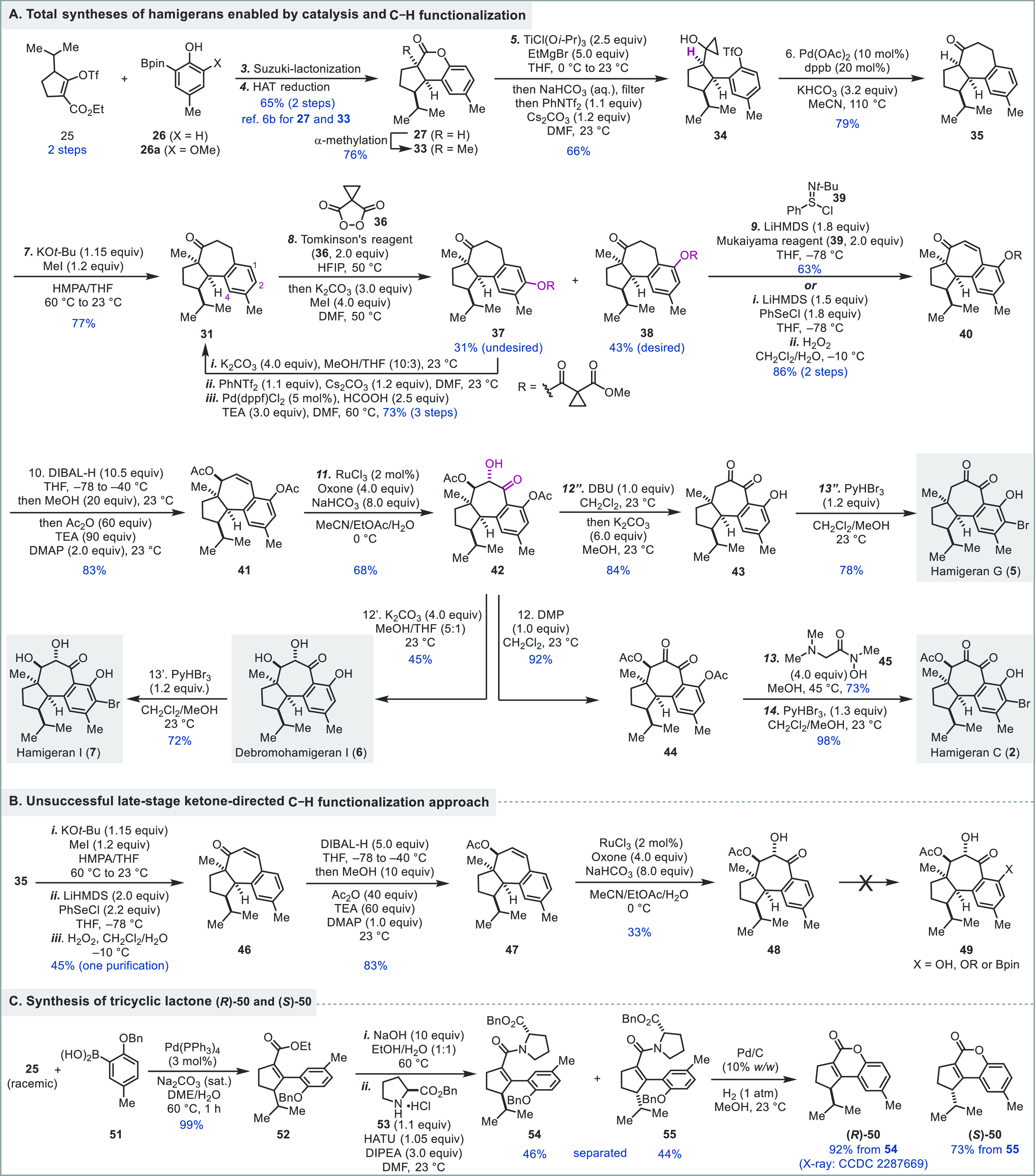
Total Syntheses of Hamigerans C, G, I, and Debromohamigeran I

The failure of the ketone-directed C–H functionalization at C1 forced us to introduce the C1 oxygenation at an earlier stage. While we could introduce it as a methoxy group at the very beginning and use **26a** (X = OMe) for the Suzuki cross-coupling with **25**, similar to what we encountered in the hamigeran M synthesis, the methoxy group was detrimental to the Pd-catalyzed cyclopropanol ring opening cross-coupling to close the seven-membered ring by inhibiting the oxidative addition step. Thus, after examination of the potential synthetic intermediates, we decided to explore C–H oxidation on **31** because it has fewer functional groups than the late-stage intermediates and could potentially tolerate harsh oxidation conditions. Meanwhile, we were aware of the challenges associated with C–H oxidation at C1 with **31**. First, there is no directing group, so regioselectivity could be an issue because there are three positions (C1, C2, and C4) on the aromatic ring. While the C4 position is more sterically hindered, the C1 and C2 positions are very similar sterically and electronically. Second, there are three benzylic positions that could potentially compete with the desired C–H oxidation at C1. Third, the ketone functionality may complicate the oxidation. With these concerns in mind, we explored the metal-free peroxide oxidation of aromatic C–H bonds developed by Siegel et al.^16^ While the phthaloyl peroxide and its derivatives failed the task, the Tomkinson malonoyl peroxide **36** turned out to be suitable.^17^ With 2.0 equiv of **36** in HFIP at 50 °C, **31** was oxidized to a mixture of **38** (desired, 43%) and **37** (undesired, 31%) in 74% total yield after the cyclopropane carboxylic acid was capped as a methyl ester with MeI and K_2_CO_3_. While the regioselectivity was only about 1.4:1, both regioisomers can be separated, and the undesired one (**37**) can be recycled back to **31** via a three-step sequence in 73% yield. The sequence involves the hydrolysis of **37**, triflate formation, and Pd-catalyzed triflate reduction. Notably, in their synthesis of cephanolide A, Sarpong and co-workers used a similar C–H oxidation to introduce a hydroxy group at the last step. In their case, the C–H oxidation prefers the C–H bond next to the methyl group with a 6:1 selectivity.^18^ Additionally, the same C–H oxidation on enone **46** led to decomposition. We also tried to block the C2 position by using a bromide, but unfortunately, the bromide blocked the reactivity at the C1 position, and no C–H oxidation occurred at C1 anymore.

With **38** in hand, we moved on to introduce other oxygen functionalities on the seven-membered ring. Desaturation of **38** to enone **40** was successful using both the one-step Mukaiyama procotol^19^ with reagent **39** or the two-step Sharpless selenoxide elimination.^12^ Stereoselective reduction of the ketone and removal of the cyclopropane 1,1-dicarboxylate were achieved by DIBAL-H reduction. The resulting intermediate was trapped in situ with acetic anhydride to provide **41** in 83% yield, which was further oxidized to α-hydroxy ketone **42** with a combination of RuCl_3_ and Oxone. Interestingly, in this case, the Plietker protocol gave a higher yield (68%) and the other α-hydroxy ketone isomer and the dihydroxylation product were barely noticeable. Compound **42** served as a key intermediate to several hamigeran natural products. A 1,8-diazabicyclo(5.4.0)undec-7-ene (DBU)-promoted β-acetate elimination followed by hydrolysis of the C1 acetate gave **43** in 84% yield. Bromination of **43** with PyHBr_3_ completed a 13-step total synthesis of hamigeran G (**5**), which is significantly shorter than the previous 24-step synthesis.^5a^ Hydrolysis of both acetates of **42** with K_2_CO_3_ in MeOH/THF gave debromohamigeran I (**6**) in 45% yield, which was further brominated to deliver hamigeran I (**7**) in 13 steps. Oxidation of **42** with Dess-Martin periodinane (DMP) gave 1,2-diketone **44** in 92% yield. At this stage, selective hydrolysis of the aryl acetate was required and achieved in 73% yield using hydroxamic acid **45** in MeOH.^20^ A subsequent bromination gave hamigeran C (**2**) in 14 steps. In addition, we established an approach to synthesize both enantiomers of **50**, the precursor of **27**, for the asymmetric synthesis of these hamigerans (Scheme 1C). Suzuki reaction between **25** and **51** gave **52**. After hydrolysis of the ester, the resulting carboxylic acid reacted with benzyl l-prolinate (**53**) to give a pair of diastereomers **54** (46%) and **55** (44%), which after benzyl group removal spontaneously cyclized to give (*R*)-**50** (92%, CCDC 2287669) and (*S*)-**50** (73%), respectively.

In summary, total syntheses of hamigerans C (**2**), G (**5**), I (**7**), and debromohamigeran I (**6**) in 12 to 14 steps were achieved. Among them, hamigerans C (**2**), I (**7**), and debromohamigeran I (**6**) were made for the first time. Our synthesis was enabled by a Pd-catalyzed intramolecular cyclopropanol ring opening cross-coupling to form the seven-membered ring (cyclase phase), a metal-free C–H oxidation using Tomkinson malonoyl peroxide, and a Ru-catalyzed regio- and stereoselective ketohydroxylation to introduce the acyloin on the seven-membered ring (oxidase phase). This work highlights how modern transition metal catalysis, C–H functionalization, and the two-phase terpene synthesis logic^21^ can impact the design and the efficiency of natural product synthesis.

## Supporting Information Available

Experimental procedures and NMR spectra for all new compounds (PDF)
