# Supplementary material for: Concise Total Syntheses of the 6–7–5 Hamigeran Natural Products
Source: J Am Chem Soc. 2023 Aug 21;145(34):18731–6. doi: 10.1021/jacs.3c06031 (PMC10472436; doi:10.1021/jacs.3c06031)
Supplement: Supplementary file 1 — ja3c06031_si_001.pdf [file ja3c06031_si_001.pdf]

Supporting Information

Concise Total Syntheses of the 6–7–5 Hamigeran Natural Products

Baiyang Jiang,[1] and Mingji Dai*,[1,2]

[1]Department of Chemistry and Center for Cancer Research, Purdue University, West Lafayette, IN 47907, United States

[2]Department of Chemistry and Department of Pharmacology and Chemical Biology, Emory University, Atlanta, GA 30322, United States

Table of Contents

Part 1. Experimental procedures and spectra data..S3

Part 2. References..S30

Part 3. ^{1}H and ^{13}C NMR spectra...S31

Part 1. Experimental procedures and spectra data.

General Methods. NMR spectra were recorded on Bruker spectrometers (^{1}H at 400 MHz, 500 MHz, 800 MHz and ^{13}C at 100 MHz, 126 MHz, 200 MHz). Chemical shifts (δ) were given in ppm with reference to solvent signals [^{1}H NMR: $CHCl_3$ (7.26); ^{13}C NMR: $CDCl_3$ (77.2), C_6D_6 (128.02), CD_3OD (49.0)]. High-resolution mass measurements for compound characterization were carried out using a Waters SYNAPT G2-Si system with QuanTof analyzer or an Agilent 6550 QTOF system. Column chromatography was performed on silica gel. All reactions requiring heating were heated in an oil bath. All reactions sensitive to air or moisture were conducted under argon atmosphere in dry and freshly distilled solvents under anhydrous conditions, unless otherwise noted. Anhydrous THF and toluene were distilled over sodium benzophenone ketyl under Argon. Anhydrous CH_2Cl_2 was distilled over calcium hydride under Argon. All other solvents and reagents were used as obtained from commercial sources without further purification. Room temperature is about 23 °C.

Experiment procedure

Compounds 27 and **33** were prepared according to the reported procedure.[1]

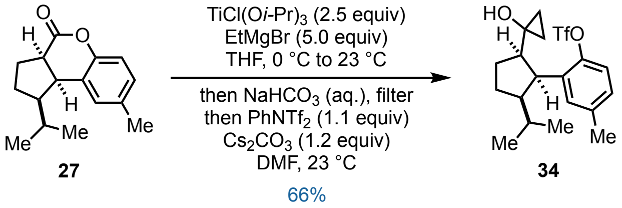

Compound 34[2,3]**:** To a 100 mL flask were added compound **27** (1.00 g, 4.09 mmol, 1.0 equiv) and THF (100 mL). The solution was then cooled down to 0 °C and TiCl(O*i*Pr)$_3$ (2.4 mL, 10.0 mmol, 2.5 equiv) was added. At the same temperature, EtMgBr (20.5 mL, 1.0 M in THF, 20.5 mmol, 5.0 equiv) was slowly added via a syringe pump during 1 h and 15 min. Upon completion of addition, the mixture was slowly warmed up to room temperature and stirred overnight. Then aqueous saturated sodium bicarbonate (2.5 mL) was slowly added. The resulting suspension was diluted with ether (50 mL), filtered through a bi-layer column of Na_2SO_4 (top-layer) and celite (bottom-layer) and concentrated. The residue was dissolved in dry DMF (15 mL) and $PhNTf_2$ (1.61 g, 4.51 mmol, 1.1 equiv) and Cs_2CO_3 (1.60 g, 4.91 mmol, 1.2 equiv) were added.

The reaction was stirred at room temperature for 30 min before it was quenched with aqueous saturated sodium bicarbonate (20 mL) and extracted with ether (20 mL × 3). The combined organic phase was washed with aqueous saturated sodium bicarbonate (20 mL × 3), dried (Na_2SO_4), filtered and concentrated. The residue was purified on column chromatography (hexanes/EtOAc = 97/3 to 95/5). Compound **34** was obtained as white solid (1.10 g, 66%). **^{1}H NMR (500 MHz, $CDCl_3$)** δ 7.18 (d, J = 8.3 Hz, 1H), 7.15 – 7.07 (m, 2H), 3.74 (dd, J = 6.5, 6.5 Hz, 1H), 2.36 (s, 3H), 2.23 (ddd, J = 15.8, 12.5, 7.6 Hz, 1H), 2.14 (dtd, J = 13.5, 9.6, 7.4 Hz, 1H), 2.03 – 1.92 (m, 1H), 1.89 – 1.79 (m, 2H), 1.77 – 1.68 (m, 1H), 1.46 – 1.36 (m, 1H), 0.90 (d, J = 6.5 Hz, 3H), 0.71 (d, J = 6.5 Hz, 3H), 0.61 (ddd, J = 10.7, 6.6, 5.4 Hz, 1H), 0.48 (ddd, J = 10.0, 6.7, 5.3 Hz, 1H), 0.40 (m, 1H), 0.15 (ddd, J = 10.2, 6.6, 5.2 Hz, 1H); **^{13}C NMR (126 MHz, $CDCl_3$)** δ 146.5, 137.3, 133.7, 132.4, 128.6, 121.0, 118.4 (q, J = 317.5 Hz), 54.5, 53.1, 53.1, 41.8, 29.4, 29.1, 24.6, 22.0, 21.7, 21.4, 12.9, 12.8; **^{19}F NMR (471 MHz, $CDCl_3$)** δ -75.7; **IR (film)**: 2957, 2877, 1489, 1414, 1206, 1139, 1084, 880 cm^{-1}; **HRMS (APCI):** *m/z* Calc. for $C_{19}H_{26}F_3O_4S$ [M+H]$^+$: 407.1504, found: 407.1515.

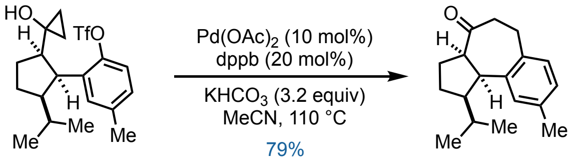

34 35

Compound 35[4]: To a seal-tube were added compound **34** (2.82 g, 6.97 mmol, 1 equiv), $Pd(OAc)_2$ (156.5 mg, 0.697 mmol, 10 mol%), dppb (594.7 mg, 1.39 mmol, 20 mmol%), $KHCO_3$ (2.23 g, 22.3 mmol, 3.2 equiv) and freshly distilled MeCN (90 mL) in the glovebox. The reaction tube was then sealed, heated to 110 °C and stirred for 19 h before it was cooled down to room temperature. The resulting suspension was diluted with DCM (90 mL), filtered through celite, and evaporated to dryness. The residue was purified on column chromatography (hexanes/DCM = 3/2 to 1/1). Compound **35** was obtained as white solid (1.41 g, 79%). **^{1}H NMR (500 MHz, $CDCl_3$)** δ 7.13 (s, 1H), 7.02 (d, J = 7.6 Hz, 1H), 6.91 (d, J = 7.6 Hz, 1H), 3.90 – 3.84 (m, 1H), 3.35 (dt, J = 14.1, 9.3 Hz, 1H), 3.14 – 3.05 (m, 1H), 2.86 – 2.71 (m, 2H), 2.53 (ddd, J = 14.6, 9.7, 8.7 Hz, 1H), 2.28 (s, 3H), 2.24 – 2.14 (m, 1H), 2.06 – 1.84 (m, 4H), 1.81 – 1.71 (m, 1H), 0.99 (d, J = 6.5 Hz, 3H), 0.83 (d, J = 6.5 Hz, 3H). **^{13}C NMR (126 MHz,**

$CDCl_3$) δ 210.7, 138.3, 136.4, 135.6, 129.0, 128.1, 127.1, 58.2, 54.1, 44.5, 42.6, 28.6, 28.3, 27.2, 24.1, 22.5 (two carbons), 21.5; **IR (film)**: 2956, 2870, 1699, 1466, 818 cm^{-1}; **HRMS (APCI):** *m/z* Calc. for $C_{18}H_{25}O_1$ $[M+H]^+$: 257.1905, found: 257.1909.

Note: The TLC sampling must be done in the glovebox to avoid decomposition.

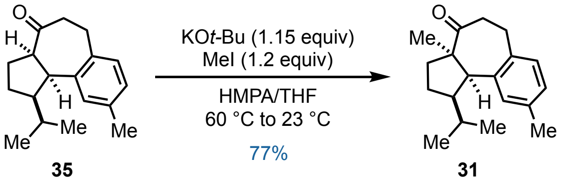

Compound 31: To a reaction flask charged with compound **35** (1.25 g, 4.88 mmol, 1.0 equiv) and KO*t*Bu (629.2 mg, 5.61 mmol, 1.15 equiv) were added THF (20 mL) and HMPA (4 mL) at room temperature. The mixture was then heated to 60 °C and stirred for 3 h before it was cooled to 0 °C. MeI (0.36 mL, 5.8 mmol, 1.2 equiv) was added and the mixture was then warmed to room temperature and stirred for 30 min before it was quenched by pouring into saturated aqueous ammonium chloride (20 mL). The residue was extracted with ether (20 mL × 3) and the combined organic phase was washed with brine, dried (Na_2SO_4), filtered and concentrated. The residue was purified by column chromatography (hexanes/EtOAc = 97/3). Compound **31** was obtained as a colorless solid (1.01 g, 77%). **^{1}H NMR (500 MHz, 323 K, $CDCl_3$)** δ 7.12 (s, 1H), 7.01 (d, *J* = 7.5 Hz, 1H), 6.90 (d, *J* = 7.6 Hz, 1H), 3.47 – 3.19 (m, 2H), 2.99 (brs, 1H), 2.73 (brs, 1H), 2.49 – 2.32 (brs, 1H), 2.27 (s, 3H), 2.27 – 2.11 (m, 3H), 2.11 – 2.00 (m, 1H), 1.84 – 1.71 (m, 1H), 1.57 – 1.46 (m, 1H), 1.38 (s, 3H), 1.00 (brs, 3H), 0.75 (d, *J* = 6.2 Hz, 3H); **^{13}C NMR (126 MHz, 323 K, $CDCl_3$)** δ 213.1, 139.1, 137.0, 135.4, 128.5, 127.1 (two carbons), 60.6, 50.4 (two carbons), 40.6, 31.9, 28.6, 28.5, 27.0, 24.9, 22.5, 22.3, 21.4; **IR (film)**: 2956, 2871, 1697, 1463, 816 cm^{-1}; **HRMS (APCI):** *m/z* Calc. for $C_{19}H_{27}O$ $[M+H]^+$: 271.2062, found: 271.2066.

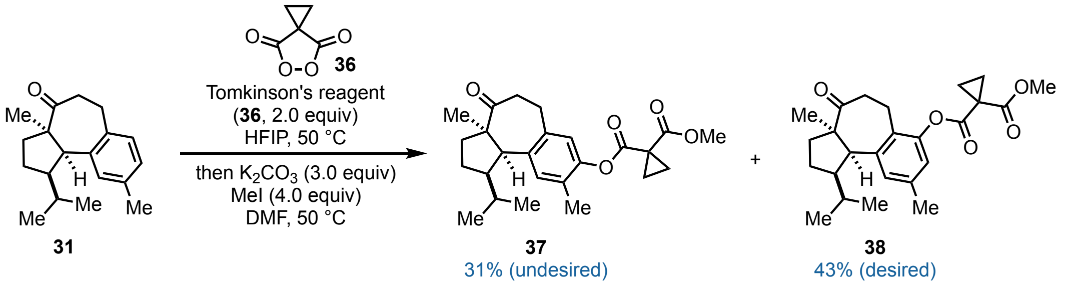

Compounds 37 and 38[5]**:** To compound **31** (201.4 mg, 0.744 mmol, 1.0 equiv) in a flame-dried seal-tube was added Tomkinson's reagent (**36**, 190.8 mg, 1.49 mmol, 2.0 equiv) and HFIP (4 mL). The reaction tube was sealed and warmed up to 50 °C. The reaction was stirred for 60 h before it was cooled down to room temperature and evaporated to dryness. Then to the residue was added DMF (2 mL), K_2CO_3 (308.8 mg, 2.23 mmol, 3.0 equiv) and MeI (0.18 mL, 2.9 mmol, 4.0 equiv). The resulting mixture was stirred at room temperature for 6 h and 30 min before it was filtered through celite, quenched with saturated aqueous ammonium chloride (5 mL) and extracted with ether (5 mL × 3). The combined organic phase was washed with brine, dried (Na_2SO_4), filtered and concentrated. The residue was purified by column chromatography (hexanes/DCM/EtOAc = 15/2/1 to 12/2/1). Compound **38** was obtained in 43% yield (133.5 mg) and compound **37** was obtained as a white solid (96.5 mg, 31%).

Compound **38**: **^{1}H NMR (500 MHz, CDCl$_3$)** *δ* 7.03 (brs, 1H), 6.69 (s, 1H), 3.80 (s, 3H), 3.39 (brs, 1H), 3.09 – 2.91 (m, 3H), 2.36 – 2.08 (m, 4H), 2.27 (s, 3H), 2.08 – 2.00 (m, 1H), 1.78 – 1.65 (m, 1H), 1.63 – 1.56 (m, 4H), 1.56 – 1.48 (m, 1H), 1.38 (s, 3H), 1.00 (brs, 3H), 0.73 (d, *J* = 5.9 Hz, 3H); **^{13}C NMR (126 MHz, CDCl$_3$)** *δ* 213.0, 170.0, 168.6, 147.8, 141.1, 136.3, 129.0, 125.6, 120.5, 60.8, 52.8, 50.2, 50.1, 39.3, 31.5, 28.6, 28.2, 26.1, 24.8, 22.6, 22.3, 21.5, 20.4, 17.2, 17.1; **IR (film)**: 2957, 2871, 1731, 1697, 1438, 1316, 1206, 1179, 1117 cm^{-1}; **HRMS (APCI):** *m/z* Calc. for $C_{25}H_{33}O_5$ $[M+H]^+$: 413.2328, found: 413.2328.

Compound **37: ^{1}H NMR (500 MHz, CDCl$_3$)** *δ* 7.15 (brs, 1H), 6.85 (s, 1H), 3.79 (s, 3H), 3.46 – 3.21 (m, 2H), 3.01 (brs, 1H), 2.66 (brs, 1H), 2.35 (brs, 1H), 2.25 – 2.15 (m, 2H), 2.12 (s, 3H), 2.11 – 1.97 (m, 2H), 1.79 – 1.65 (m, 1H), 1.64 – 1.55 (m, 4H), 1.55 – 1.46 (m, 1H), 1.36 (s, 3H), 0.99 (s, br3H), 0.73 (d, *J* = 5.8 Hz, 3H); **^{13}C NMR (126 MHz, CDCl$_3$)** *δ* 213.1, 170.0, 168.1, 147.3, 139.0, 137.1, 129.5, 127.2, 121.6, 60.9, 52.7, 50.0, 49.8, 40.2, 31.4, 28.6, 28.3, 28.1, 26.2, 24.7, 22.6, 22.4, 17.1 (two carbons), 16.2; **IR (film)**: 2957, 2872, 1732, 1698, 1437, 1314, 1207, 1186, 1119 cm^{-1}; **HRMS (APCI):** *m/z* Calc. for $C_{25}H_{33}O_5$ $[M+H]^+$: 413.2328,

found: 413.2344.

Due to safety concerns of using peroxide, a two-step procedure for large-scale reaction is described as follows:

To compound **31** (668.8 mg, 2.47 mmol, 1.0 equiv) in a flame-dried microwave-tube was added Tomkinson's reagent (633.5 mg, 4.95 mmol, 2.0 equiv) and HFIP (14 mL). The reaction tube was sealed and warmed up to 50 °C with a microwave-reactor. The reaction was stirred for 20 h before it was cooled down to room temperature and poured into aqueous saturated $Na_2S_2O_5$ (40 mL). The residue was extracted with ether (30 mL × 3) and the combined organic phase was washed with aqueous saturated $Na_2S_2O_5$ and brine, dried (Na_2SO_4), filtered through celite and concentrated. Then to the residue was added DMF (16 mL), K_2CO_3 (1.37 g, 9.91 mmol, 4.0 equiv) and MeI (1.23 mL, 19.7 mmol, 8.0 equiv). The resulting mixture was stirred at 50 °C for 1 h before it was poured into saturated aqueous ammonium chloride (100 mL) and extracted with ether (80 mL ×3). The combined organic phase was washed with brine, dried (Na_2SO_4), filtered and concentrated. The residue was purified by column chromatography (hexanes/DCM/EtOAc = 12/2/1). Compound **38** was obtained as a colorless oil (467.1 mg, 46%) and compound **37** was obtained as a white solid (363.6 mg, 36%).

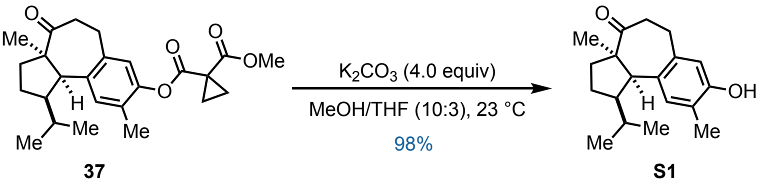

Compound S1: To compound **37** (180.5 mg, 0.438 mmol, 1.0 equiv) in a co-solvent of MeOH (5 mL) and THF (1.5 mL) was added K_2CO_3 (241.9 mg, 1.75 mmol, 4.0 equiv). The mixture was stirred at room temperature for 60 min before it was filtered through celite, quenched with saturated aqueous ammonium chloride (10 mL) and extracted with ether (10 mL × 3). The combined organic phase was washed with brine, dried (Na_2SO_4), filtered and concentrated. The residue was purified on column chromatography (hexanes/EtOAc = 4/1) and **compound S1** was obtained as a white solid (123.3 mg, 98%). **^{1}H NMR (500 MHz, $CDCl_3$)** δ 7.05 (brs, 1H), 6.57 (s, 1H), 4.55 (s, 1H), 3.48 – 3.23 (m, 2H), 3.02 (brs, 1H), 2.60 (brs, 1H), 2.35 (brs, 1H),

2.26 – 2.09 (m, 3 H), 2.16 (s, 3H), 2.09 – 1.96 (m, 1H), 1.82 – 1.67 (m, 1H), 1.56 (s, 3H), 1.54 – 1.45 (m, 1H), 1.35 (s, 3H), 1.00 (brs, 3H), 0.74 (d, *J* = 5.8 Hz, 3H); **^{13}C NMR (126 MHz, CDCl$_3$)** δ 213.7, 152.0, 139.1, 131.3, 129.5, 120.7, 115.4, 60.9, 50.0, 49.5, 40.5, 31.4, 28.6, 28.4, 26.4, 24.6, 22.6, 22.3, 15.8; **IR (film)**: 3378, 2959, 2871, 1684, 1500, 1460, 1415, 1287, 1201, 1154, 734 cm^{-1}; **HRMS (APCI):** *m/z* Calc. for $C_{19}H_{27}O_2$ [M+H]$^+$: 287.2011, found: 413.2001.

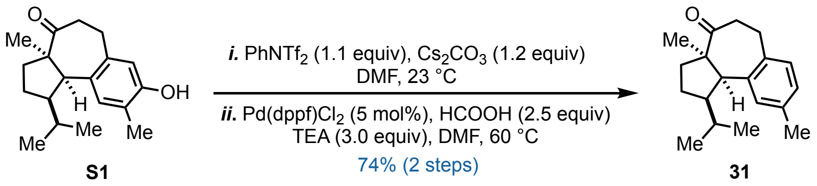

Compound 31[6]: To compound **S1** (17.7 mg, 0.0618 mmol, 1.0 equiv) in DMF (1 mL) were added $PhNTf_2$ (24.3 mg, 0.0680 mmol, 1.1 equiv) and Cs_2CO_3 (24.2 mg, 0.0743 mmol, 1.2 equiv). The mixture was stirred at room temperature for 2 h before it was quenched with saturated aqueous ammonium chloride (1 mL) and extracted with ether (3 mL × 3). The combined organic phase was washed with brine, dried (Na_2SO_4), filtered and concentrated. The crude residue was used in the next step without further purification.

To the above crude triflate was added Pd(dppf)Cl_2 (2.5 mg, 0.0031, 5% mol) and the reaction vial was charged with argon. DMF was added to dissolve the reagents, and then triethylamine (26 μL, 0.19 mmol, 3.0 equiv) and formic acid (6.0 μL, 0.16 mmol, 2.5 equiv) were added. The resulting mixture was warmed up to 60 ˚C and stirred for 17.5 h before it was quenched with water (1 mL) and extracted with ether (2 mL × 3). The combined organic phase was washed with brine, dried (Na_2SO_4), filtered and concentrated. The residue was purified by column chromatography (hexanes/EtOAc = 97/3). Compound **31** was obtained as a colorless solid (12.4 mg, 74%).

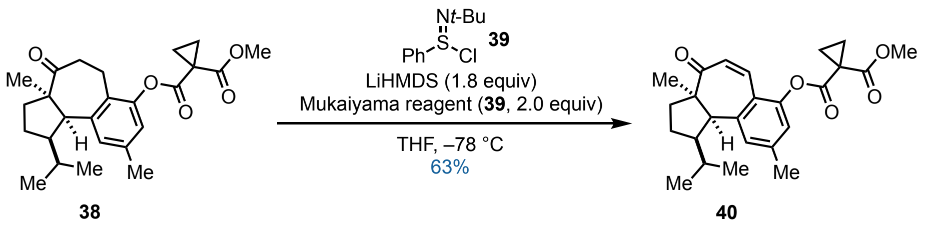

Compound 40[7]**:** To compound **38** (23.7 mg, 0.0502 mmol, 1.0 equiv) in THF (1 mL) was added LiHMDS (0.090 mL, 1 M in THF, 0.090 mmol, 1.8 equiv) at −78 °C under argon. The reaction was stirred at this temperature for 90 min. Mukaiyama's reagent (**39**, 27.1 mg, 80%, 0.100 mmol, 2.0 equiv) in THF (0.3 mL) was added dropwise to the reaction solution. The mixture was stirred at −78 °C for 60 min before it was quenched with saturated aqueous ammonium chloride (1 mL) and extracted with ether (1 mL × 3). The combined organic phase was washed with brine, dried (Na_2SO_4), filtered and concentrated. The residue was purified on column chromatography (hexanes/EtOAc = 10/1) and compound **40** was obtained as a yellow oil (13.0 mg, 63%). **^{1}H NMR (500 MHz, $CDCl_3$)** δ 7.24 (d, 1H), 7.01 (s, 1H), 6.82 (s, 1H), 6.11 (d, J = 13.1 Hz, 1H), 3.83 (s, 3H), 3.23 (d, J = 10.4 Hz, 1H), 2.61 – 2.55 (m, 1H), 2.34 (s, 3H), 2.21 – 2.11 (m, 1H), 1.93 – 1.84 (m, 1H), 1.67 – 1.62 (m, 4H), 1.50 – 1.35 (m, 2H), 1.35 – 1.26 (m, 1H), 1.15 (s, 3H), 0.63 (d, J = 6.5 Hz, 3H), 0.31 (d, J = 6.3 Hz, 3H); **^{13}C NMR (126 MHz, $CDCl_3$)** δ 204.9, 169.7, 168.3, 150.1, 142.9, 141.1, 132.8, 132.1, 129.6, 124.5, 121.4, 58.7, 58.0, 52.9, 52.1, 38.7, 31.1, 28.7, 28.2, 23.7, 23.2, 21.6, 21.3, 17.3 (two carbons); **IR (film)**: 2955, 2868, 1732, 1647, 1613, 1439, 1347, 1314, 1258, 1204, 1178, 1108 cm^{-1}; **HRMS (APCI):** *m/z* Calc. for $C_{25}H_{31}O_5$ $[M+H]^+$: 411.2171, found: 411.2177.

The two-step procedure with PhSeCl is described below.

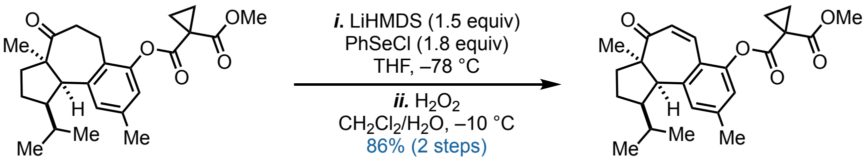

38 **40**

To compound **38** (454.5 mg, 1.10 mmol, 1.0 equiv) in THF (6 mL) was added LiHMDS (1.65 mL, 1 M in THF, 1.65 mmol, 1.5 equiv) at −78 °C under argon. The reaction was stirred at this temperature for 2 h. PhSeCl (379.8 mg, 1.98 mmol, 1.8 equiv) in THF (2 mL) was added dropwise to the reaction solution. The mixture was stirred at −78 °C for 30 min before it was quenched with saturated aqueous ammonium chloride (6 mL) and extracted with ether (5 mL × 3). The combined organic phase was washed with brine, dried (Na_2SO_4), filtered and concentrated. The crude residue was dissolved in DCM (6 mL) and cooled down to −15 °C and H_2O_2 (2 mL, 30% in water) was slowly added. The mixture was warmed to −10 °C and stirred

for 20 min before it was carefully quenched with saturated aqueous sodium bicarbonate (10 mL) and extracted with DCM (10 mL × 3). The combined organic phase was washed with 10% $Na_2S_2O_5$ (20 mL) and brine, dried (Na_2SO_4), filtered and concentrated. The residue was purified on column chromatography (hexanes/EtOAc = 9/1) and compound **40** was obtained as a yellow oil (390.3 mg, 86%).

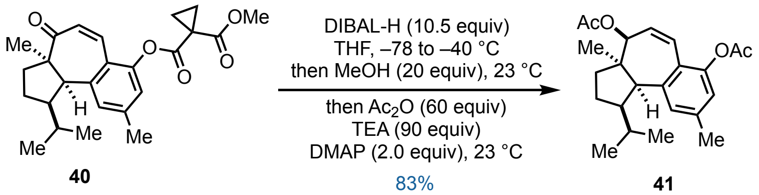

Compound 41[8]: To a flame-dried vial were added compound **40** (13.0 mg, 0.0317 mmol, 1.0 equiv) and THF (1 mL). The mixture was cooled down to −78 °C and DIBAL-H (0.10 mL, 1 M in toluene, 0.1 mmol, 3 equiv) was added dropwise. The resulting mixture was stirred at the same temperature for 1 h, and then a second portion of DIBAL-H (0.10 mL, 1 M in toluene, 0.1 mmol, 3 equiv) was added. The resulting mixture was stirred at the same temperature for 40 min and then was warmed up to −40 °C. After 1 h, a third portion of DIBAL-H (0.05 mL, 1 M in toluene, 0.05 mmol, 1.5 equiv) was added. After another 30 min, a final portion of DIBAL-H (0.10 mL, 0.1 M in toluene, 0.1 mmol, 3 equiv) was added. The reaction was further stirred for 30 min before it was quenched with MeOH (26 μL, 0.64 mmol, 20 equiv) and warmed up to room temperature. Triethylamine (0.40 mL, 2.9 mmol, 90 equiv), acetic anhydride (0.18 mL, 1.9 mmol, 60 equiv) and DMAP (7.8 mg, 0.064 mmol, 2.0 equiv) was subsequently added to the reaction mixture. The reaction was further stirred at room temperature for 12 h before it was quenched with saturated aqueous sodium bicarbonate (6 mL) and extracted with EtOAc (8 mL × 3). The combined organic phase was washed with brine (10 mL), dried (Na_2SO_4), filtered and concentrated. The residue was purified on column chromatography (hexanes/EtOAc = 8/1). Compound **41** was obtained as a white solid (9.7 mg, 83%). **^{1}H NMR (500 MHz, $CDCl_3$)** δ 6.87 (s, 1H), 6.79 (s, 1H), 6.34 (dd, J = 10.9, 2.3 Hz, 1H), 5.78 (dd, J = 10.9, 4.9 Hz, 1H), 5.13 – 5.00 (m, 1H), 3.11 (d, J = 11.0 Hz, 1H), 2.40 – 2.29 (m, 1H), 2.31 (s, 3H), 2.26 (s, 3H), 2.06 (s, 3H), 1.99 – 1.88 (m, 1H), 1.78 – 1.71 (m, 2H), 1.41 – 1.31 (m, 1H), 1.26 – 1.12 (m, 1H), 1.17 (s, 3H), 0.70 (d, J = 6.6 Hz, 3H), 0.35 (d, J = 6.3 Hz,

3H); **^{13}C NMR (126 MHz, $CDCl_3$,** one peak covered by the solvent peak**)** δ 170.3, 169.0, 148.0, 141.6, 137.6, 135.0, 132.3, 127.0, 123.7, 120.9, 64.7, 59.5, 55.0, 35.8, 35.2, 28.8, 28.1, 22.7, 22.2, 21.2, 21.1, 20.9; **IR (film)**: 2956, 2868, 1767, 1735, 1446, 1369, 1239, 1201, 1025, 908, 730 cm^{-1}; **HRMS (APCI):** *m/z* Calc. for $C_{23}H_{31}O_4$ $[M+H]^+$: 371.2222, found: 371.2211.

A two-step procedure is described below.

To a flame-dried vial were added compound **40** (356.9 mg, 0.869 mmol, 1.0 equiv) and THF (10 mL). The mixture was cooled down to −78 °C and DIBAL-H (2.6 mL, 1 M in toluene, 2.6 mmol, 3 equiv) was added dropwise. The resulting mixture was stirred at the same temperature for 70 min, and then a second portion of DIBAL-H (2.6 mL, 1 M in toluene, 2.6 mmol, 3 equiv) was added. The resulting mixture was stirred at the same temperature for 80 min before it was quenched with MeOH (15 ml) and warmed up to room temperature. Then K_2CO_3 (0.48 g, 3.47 mmol, 4 equiv) was added to promote phenol deprotection. After stirring for 60 min, the resulting mixture was poured into saturated aqueous ammonium chloride (100 mL) and extracted with ether (80 mL × 3). The combined organic phase was washed with saturated sodium bicarbonate (100 mL), dried (Na_2SO_4), filtered and concentrated. The crude product was further dried under vacuum to remove the solvent and was then used without further purification.

The above crude residue was dissolved in DCM (10 mL) and cooled down to 0 °C. DMAP (421.0 mg, 3.48 mmol, 4.0 equiv) and acetic anhydride (0.49 mL, 5.24 mmol, 6.0 equiv) were added sequentially. The reaction mixture was stirred at the same temperature for 30 min before it was quenched with saturated aqueous sodium bicarbonate (10 mL) and extracted with DCM (10 mL × 3). The combined organic phase was washed with brine (2 mL), dried (Na_2SO_4), filtered and concentrated. The residue was purified on column chromatography (hexanes/EtOAc = 8/1). Compound **41** was obtained as a white solid (273.6 mg, 85%).

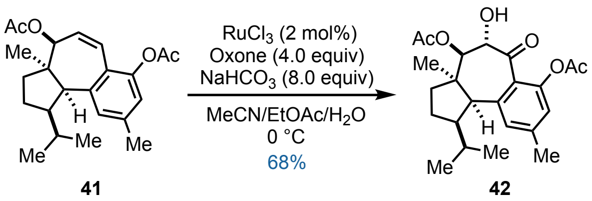

Compound 42[9]: To a reaction vial were added oxone (199.4 mg, 0.324 mmol, 4.0 equiv) and

$NaHCO_3$ (54.4 mg, 0.648 mmol, 8.0 equiv). Water (0.15 mL) was added, and the resulting mixture was kept at room temperature for 5 min while CO_2 was generated vigorously. When the gas release slowed down, MeCN (0.90 mL) and EtOAc (0.90 mL) was added. Compound **41** (30.0 mg, 0.0810 mmol, 1.0 equiv) was added to the suspension and the mixture was cooled down to 0 °C. To another vial were added $RuCl_3 \cdot xH_2O$ (4.0 mg, Ruthenium content 38-42%, 0.016 mmol, 20 mol%), $NaHCO_3$ (54.4 mg, 0.648 mmol, 8 equiv), water (0.15 mL), MeCN (0.9 mL) and EtOAc (0.9 mL). Oxone (199.4 mg, 0.324 mmol, 4 equiv) was added to the mixture and the vial was gently shaken until the suspension turned bright yellow. Carefully transferred the solution with freshly prepared Ru species (0.2 mL, containing 0.0016 mmol [Ru], 2 mol%) to the reaction vial, and the resulting mixture was stirred at 0 °C for 75 min before it was quenched with aqueous $NaHSO_3$ (10%, 3 mL) and extracted with ether (5 mL × 3). The combined organic phase was washed with aqueous $NaHSO_3$ (10%, 10 mL × 2) and saturated aqueous sodium bicarbonate (10 mL × 2), dried (Na_2SO_4), filtered and concentrated. The residue was purified on column chromatography (hexanes/EtOAc = 6/1 to 4/1). Compound **42** was obtained as a white solid (22.2 mg, 68%). **^{1}H NMR (500 MHz, DMSO)** *δ* 7.07 (s, 1H), 6.94 (s, 1H), 6.28 (brs, 1H), 4.57 (brs, 1H), 3.86 (brs, 1H), 3.30 (m, 1H, covered by water peak), 2.33 (s, 3H), 2.11 (s, 3H), 2.06 – 1.84 (m, 5H), 1.63 (brs, 1H), 1.28 – 1.20 (m, 1H), 1.16 – 0.98 (m, 1H), 1.09 (s, 3H), 0.88 – 0.75 (m, 1H), 0.66 – 0.48 (m, 6H); **^{13}C NMR (126 MHz, DMSO)** *δ* 200.8, 170.3, 169.4, 147.7, 141.5, 138.8, 131.8, 127.9, 123.1, 77.8, 76.3, 58.7, 53.2, 47.0, 34.6, 32.1, 30.2, 27.7, 22.8, 22.2, 21.3, 21.0 (two carbons); **IR (film)**: 3484, 2957, 2870, 1770, 1737, 1702, 1614, 1454, 1368, 1227, 1196, 1026, 736 cm^{-1}; **HRMS (APCI):** *m/z* Calc. for $C_{23}H_{31}O_6$ $[M+H]^+$: 403.2121, found: 413.2101.

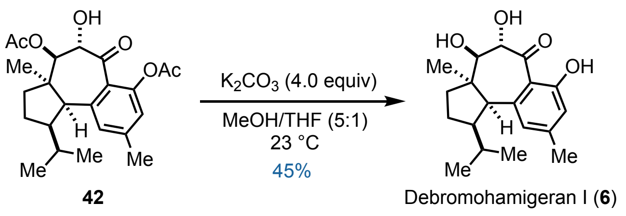

Debromohamigeran I (6): To a reaction vial flask were added compound **42** (10.1 mg, 0.0251 mmol, 1.0 equiv), THF (0.2 mL) and MeOH (1 mL). K_2CO_3 (13.8 mg, 0.0998 mmol, 4 .0 equiv) was then added and the mixture was stirred at room temperature for 60 min before it was

quenched with saturated aqueous ammonium chloride (2 mL) and extracted with ether (3 mL × 3). The combined organic phase was washed with brine (5 mL), dried (Na_2SO_4), filtered and concentrated. The residue was purified on prep. TLC (hexanes/EtOAc = 5/2). Debromohamigeran I (**6**) was obtained as a light-yellow solid (3.6 mg, 45%). **^{1}H NMR (500 MHz, CDCl$_3$)** δ 12.52 (s, 1H), 6.77 (s, 1H), 6.64 (s, 1H), 4.57 (d, J = 10.8 Hz, 1H), 4.25 (s, 1H), 3.46 (d, J = 11.6 Hz, 1H), 3.34 (d, J = 10.7 Hz, 1H), 3.18 (s, 1H), 2.61 – 2.54 (m, 1H), 2.32 (s, 3H), 2.26 – 2.16 (m, 1H), 1.97 – 1.86 (m, 1H), 1.66 – 1.53 (m, 1H), 1.47 – 1.37 (m, 2H), 1.25 (s, 3H), 1.18 – 1.08 (m, 1H), 0.77 (d, J = 5.2 Hz, 3H), 0.19 (d, J = 4.8 Hz, 3H); **^{13}C NMR (126 MHz, CDCl$_3$)** δ 204.3, 164.9, 148.5, 143.0, 127.8, 117.9, 115.4, 74.5, 74.2, 61.3, 53.8, 47.7, 34.4, 32.3, 30.0, 29.6, 23.2, 22.0, 21.8; **IR (film)**: 3444, 2925, 2868, 1616, 1566, 1544, 1454, 1333, 1298, 1251, 1196, 1112 cm^{-1}; **HRMS (APCI):** *m/z* Calc. for $C_{19}H_{27}O_4$ $[M+H]^+$: 319.1909, found: 319.1920.

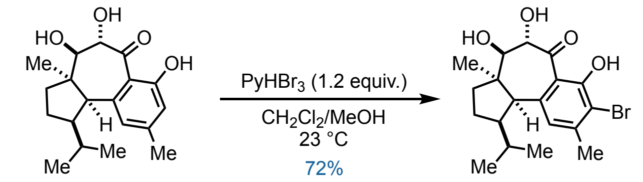

Debromohamigeran I (**6**) Hamigeran I (**7**)

Hamigeran I (7): To a reaction vial were added debromohamigeran I (**6**) (3.0 mg, 0.0094 mmol, 1.0 equiv) and a co-solvent of DCM and MeOH (DCM/MeOH = 5:2, 0.75 mL). To the reaction solution was slowly added py·HBr_3 (4.0 mg, 90%, 0.011 mmol, 1.2 equiv) dissolved in the same co-solvent (0.2 mL). After completion of addition, the mixture was stirred at room temperature for 60 min before it was quenched with aqueous $NaHSO_3$ (10%, 1 mL) and diluted with ether (2 mL). The organic phase was washed with saturated aqueous sodium bicarbonate (3 mL) and brine (3 mL), dried (Na_2SO_4), filtered and concentrated. The residue was purified on prep. TLC (hexanes/EtOAc = 3/1). Hamigeran I (**7**) was obtained as a yellow solid (2.7 mg, 72%). **^{1}H NMR (500 MHz, CDCl$_3$)** δ 13.27 (s, 1H), 6.77 (s, 1H), 4.59 (d, J = 10.7 Hz, 1H), 4.14 (s, 1H), 3.44 (d, J = 11.7 Hz, 1H), 3.33 (d, J = 10.7 Hz, 1H), 3.17 (s, 1H), 2.64 – 2.53 (m, 1H), 2.45 (s, 3H), 2.30 – 2.17 (m, 1H), 1.99 – 1.87 (m, 1H), 1.66 – 1.51 (m, 1H), 1.49 – 1.36 (m, 1H), 1.25 (s, 3H), 1.15 – 1.04 (m, 1H), 0.78 (d, J = 6.6 Hz, 3H), 0.21 (d, J = 6.1 Hz, 3H); **^{13}C NMR (126 MHz, CDCl$_3$)** δ 204.8, 160.8, 148.0, 141.7, 128.2, 116.2, 113.4, 74.3 (two carbons), 61.1, 53.9,

47.7, 34.4, 32.3, 30.0, 29.5, 23.9, 23.4, 22.0; **IR (film)**: 3398, 2923, 2853, 1616, 1444, 1385, 1251, 1190, 486 cm^{-1}; **HRMS (APCI):** *m/z* Calc. for $C_{19}H_{26}BrO_4$ $[M+H]^+$: 397.1014, found: 397.1022.

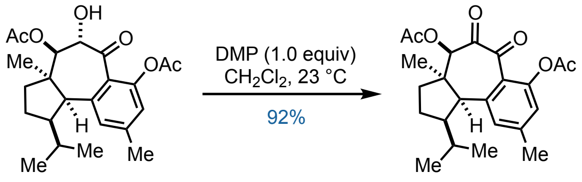

Compound 44: To a reaction vial were added compound **42** (22.2 mg, 0.0552 mmol, 1.0 equiv) and DCM (3 mL). Then Dess–Martin periodinane (23.4 mg, 0.0552 mmol, 1.0 equiv) was added and the reaction mixture was stirred at room temperature for 30 min before it was quenched with aqueous $NaHSO_3$ (10%, 2 mL) and extracted with DCM (3 mL × 3). The combined organic phase was washed with saturated aqueous sodium bicarbonate (4 mL), dried (Na_2SO_4), filtered and concentrated. The residue was purified on column chromatography (hexanes/ether = 6/1). Compound **45** was obtained as a light-yellow solid (20.4 mg, 92%). **^{1}H NMR (500 MHz, $CDCl_3$)** δ 7.00 (d, *J* = 1.6 Hz, 1H), 6.92 (d, *J* = 1.6 Hz, 1H), 5.33 (s, 1H), 3.53 (d, *J* = 11.3 Hz, 1H), 2.40 (s, 3H), 2.23 (s, 3H), 2.16 (s, 3H), 2.15 – 2.10 (m, 1H), 2.04 – 1.95 (m, 1H), 1.75 – 1.66 (m, 1H), 1.41 – 1.34 (m, 1H), 1.32 (s, 3H), 1.03 – 0.93 (m, 1H), 0.93 – 0.83 (m, 1H), 0.66 (d, *J* = 6.6 Hz, 3H), 0.42 (d, *J* = 6.3 Hz, 3H); **^{13}C NMR (126 MHz, $CDCl_3$)** *δ* 189.2, 184.8, 169.8, 169.6, 149.5, 144.6, 139.7, 132.5, 127.7, 123.1, 81.9, 59.2, 53.5, 49.3, 34.9, 31.9, 28.9, 28.4, 22.3, 22.0, 21.5, 20.9, 20.5; **IR (film)**: 2929, 1870, 1772, 1737, 1701, 1611, 1368, 1227, 1193, 1036 cm^{-1}; **HRMS (APCI):** *m/z* Calc. for $C_{23}H_{29}O_6$ $[M+H]^+$: 401.1964, found: 401.1976.

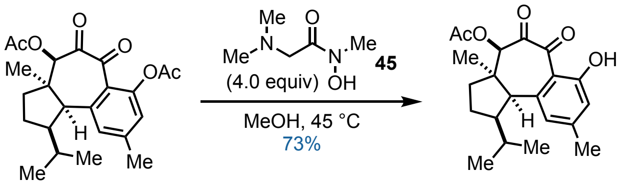

Compound S2[10]: To a reaction vial were added compound **44** (19.9 mg, 0.0497 mmol, 1.0 equiv), *N*-methyl-2-dimethylaminoacetohydroxamic acid (26.3 mg, 0.199 mmol, 4.0 equiv) and

MeOH (1 mL). The mixture was warmed up to 45 °C and stirred for 9 h before it was concentrated. The residue was purified on column chromatography (hexanes/EtOAc = 8/1). Compound **S2** was obtained as a light-yellow solid (13.0 mg, 73%). **^{1}H NMR (500 MHz, $CDCl_3$)** δ 11.37 (s, 1H), 6.84 (s, 1H), 6.67 (s, 1H), 5.12 (s, 1H), 3.49 (d, J = 11.5 Hz, 1H), 2.36 (s, 3H), 2.34 – 2.24 (m, 1H), 2.16 (s, 3H), 2.05 – 1.95 (m, 1H), 1.78 – 1.69 (m, 1H), 1.45 – 1.36 (m, 1H), 1.33 (s, 3H), 1.12 – 0.99 (m, 1H), 0.93 – 0.82 (m, 1H), 0.67 (d, J = 6.5 Hz, 3H), 0.26 (d, J = 6.1 Hz, 3H); **^{13}C NMR (126 MHz, $CDCl_3$)** δ 192.0, 187.2, 170.1, 164.3, 149.2, 141.1, 127.8, 118.0, 117.8, 81.9, 60.0, 53.2, 47.6, 34.8, 31.6, 29.7, 29.6, 28.9, 22.2, 22.1, 22.1, 20.5; **IR (film)**: 2927, 1737, 1626, 1374, 1231, 1039 cm^{-1}; **HRMS (APCI):** *m/z* Calc. for $C_{21}H_{27}O_5$ $[M+H]^+$: 359.1858, found: 359.1853.

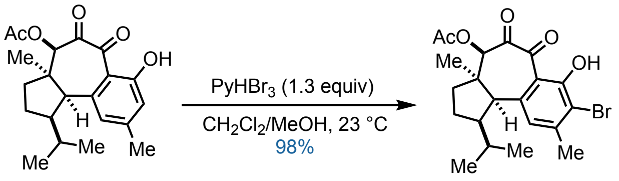

S2 Hamigeran C (**2**)

Hamigeran C (2): To a reaction vial were added **S2** (4.0 mg, 0.011 mmol, 1.0 equiv) and a co-solvent of DCM and MeOH (DCM/MeOH = 5:2, 1 mL). To the reaction solution was slowly added py·HBr_3 (5.2 mg, 90%, 0.016 mmol, 1.3 equiv) dissolved in the same co-solvent (0.5 mL). After completion of addition, the mixture was stirred at room temperature for 60 min before it was quenched with aqueous $NaHSO_3$ (10%, 2 mL) and diluted with ether (2 mL). The organic phase was washed with saturated aqueous sodium bicarbonate (3 mL) and brine (3 mL), dried (Na_2SO_4), filtered and concentrated. The residue was purified on column chromatography (hexanes/EtOAc = 10/1). Hamigeran C (**2**) was obtained as a yellow solid (4.8 mg, 98%). **^{1}H NMR (500 MHz, $CDCl_3$)** δ 12.04 (s, 1H), 6.80 (s, 1H), 5.08 (s, 1H), 3.49 (d, J = 11.2 Hz, 1H), 2.49 (s, 3H), 2.34 – 2.27 (m, 1H), 2.16 (s, 3H), 2.08 – 1.97 (m, 1H), 1.78 – 1.70 (m, 1H), 1.47 – 1.37 (m, 1H), 1.34 (s, 3H), 1.11 – 1.01 (m, 1H), 0.91 – 0.81 (m, 1H), 0.68 (d, J = 6.5 Hz, 3H), 0.27 (d, J = 6.2 Hz, 3H); **^{13}C NMR (126 MHz, $CDCl_3$)** δ 191.2, 187.5, 170.0, 160.4, 148.8, 139.7, 128.3, 118.3, 113.6, 81.7, 59.8, 53.2, 47.6, 34.8, 31.6, 29.6, 28.9, 24.0, 22.3, 22.1, 20.4; **IR (film)**: 2924, 2853, 1738, 1625, 1463, 1374, 1231, 1189, 1040 cm^{-1}; **HRMS (APCI):** *m/z* Calc. for $C_{21}H_{26}BrO_5$ $[M+H]^+$: 437.0964, found: 437.0970.

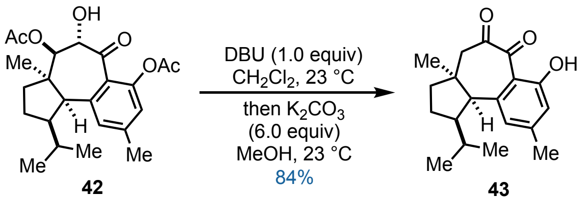

Compound 43: To a reaction vial were added compound **42** (8.5 mg, 0.021 mmol, 1.0 equiv) and DCM (1 mL). Then to the mixture were added DBU (1.6 μL, 0.011 mmol, 0.5 equiv) and the reaction was stirred at room temperature for 100 min. Then more DBU (1.6 μL, 0.011 mmol, 0.5 equiv) was added and the mixture was stirred overnight. To the resulting green solution was added MeOH (1 mL) and K_2CO_3 (17.5 mg, 0.127 mmol, 6 equiv) and the reaction was further stirred at room temperature for 1 h (during which it turned red) before it was quenched with saturated aqueous ammonium chloride (2 mL) and extracted with DCM (3 mL × 3). The combined organic phase was washed with brine (5 mL), dried (Na_2SO_4), filtered and concentrated. The residue was purified on column chromatography (hexanes/EtOAc = 15/1). Compound **43** was obtained as a yellow solid (5.3 mg, 84%). For major conformer: **^{1}H NMR (500 MHz, $CDCl_3$)** δ 11.48 (s, 1H), 6.82 (s, 1H), 6.65 (s, 1H), 3.39 (d, J = 11.2 Hz, 1H), 2.74 (d, J = 11.8 Hz, 1H), 2.55 (d, J = 11.8 Hz, 1H), 2.36 (s, 3H), 2.03 – 1.94 (m, 2H), 1.76 – 1.69 (m, 1H), 1.65 – 1.58 (m, 1H), 1.21 – 1.08 (m, 1H), 0.92 – 0.84 (m, 1H), 0.68 (d, J = 6.4 Hz, 3H), 0.25 (d, J = 6.1 Hz, 3H); **^{13}C NMR (126 MHz, $CDCl_3$)** δ 196.6, 190.5, 164.2, 148.6, 143.1, 127.5, 118.0, 117.6, 60.9, 53.6, 53.5, 45.2, 40.6, 31.8, 31.3, 29.8, 22.1, 22.1, 22.0; **IR (film)**: 2955, 2868, 1723, 1625, 1565, 1453, 1335, 1177 cm^{-1}; **HRMS (APCI):** *m/z* Calc. for $C_{19}H_{25}O_3$ $[M+H]^+$: 301.1804, found: 301.1800.

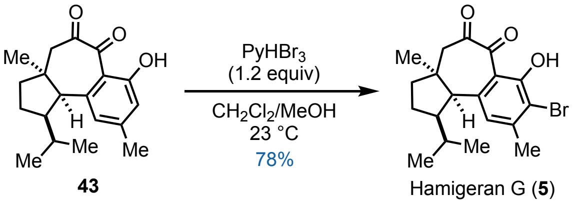

Hamigeran G (5) [11]: To a reaction vial were added compound **43** (5.3 mg, 0.018 mmol, 1.0 equiv) and a co-solvent of DCM and MeOH (DCM/MeOH = 5:2, 1 mL). To the reaction solution was slowly added py·HBr_3 (7.6 mg, 90%, 0.021 mmol, 1.2 equiv) dissolved in the same co-solvent (0.5 mL). After completion of addition, the mixture was stirred at room temperature for 30 min before it was quenched with aqueous $NaHSO_3$ (10%, 1 mL) and diluted with ether

(2 mL). The organic phase was washed with saturated aqueous sodium bicarbonate (3 mL) and brine (3 mL), dried (Na_2SO_4), filtered and concentrated. The residue was purified on prep. TLC (hexanes/EtOAc = 5/1). Hamigeran G (**5**) was obtained as a yellow solid (5.2 mg, 78%). For major conformer: **^{1}H NMR (500 MHz, CDCl$_3$)** δ 12.17 (s, 1H), 6.78 (s, 1H), 3.39 (d, J = 11.2 Hz, 1H), 2.71 (d, J = 11.9 Hz, 1H), 2.58 (d, J = 11.9 Hz, 1H), 2.48 (s, 3H), 2.08 – 1.95 (m, 2H), 1.78 – 1.71 (m, 1H), 1.62 (m, 1H), 1.29 (s, 3H), 1.21 – 1.11 (m, 1H), 0.90 – 0.80 (m, 1H), 0.69 (d, J = 6.5 Hz, 3H), 0.25 (d, J = 6.2 Hz, 3H); **^{13}C NMR (126 MHz, CDCl$_3$)** δ 195.6, 190.7, 160.3, 148.1, 141.8, 128.0, 121.7, 118.5, 113.1, 60.7, 53.5, 53.4, 45.3, 40.6, 31.8, 31.3, 29.8, 24.0, 22.2, 22.0; **IR (film)**: 2955, 2868, 1723, 1620, 1444, 1384, 1331, 1286, 1169, 734 cm^{-1}; **HRMS (APCI):** *m/z* Calc. for $C_{19}H_{24}BrO_3$ [M+H]$^+$: 379.0909, found: 379.0919.

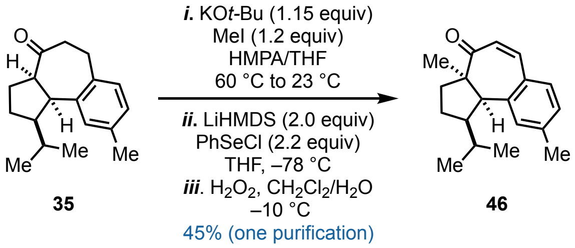

Compound 46: To a reaction flask charged with compound **35** (500.0 mg, 1.95 mmol, 1.0 equiv) and KO*t*Bu (229.7 mg, 2.05 mmol, 1.05 equiv) were added THF (10 mL) and HMPA (2 mL) at room temperature. The mixture was then warmed up to 60 °C and stirred for 1 h 20 min before it was cooled to room temperature. MeI (0.135 mL, 2.17 mmol, 1.1 equiv) was added and the mixture was stirred for 30 min before it was quenched with saturated aqueous ammonium chloride (10 mL). The residue was extracted with ether (15 mL × 3) and the combined organic phase was washed with brine, dried (Na_2SO_4), filtered and concentrated.

The above crude residue was dissolved in dry THF (6 mL), and then LiHMDS (3.9 mL, 1 M in THF, 3.9 mmol, 2.0 equiv) was added at −78 °C under argon. The reaction was stirred at this temperature for 1 h. PhSeCl (821.3 mg, 4.29 mmol, 2.2 equiv) in THF (2 mL) was added dropwise to the reaction solution. The mixture was stirred at −78 °C for 30 min before it was quenched with saturated aqueous ammonium chloride (6 mL) and extracted with ether (10 mL × 3). The combined organic phase was washed with brine, dried (Na_2SO_4), filtered and concentrated.

The above crude residue was dissolved in DCM (20 mL) and cooled down to −10 °C and H_2O_2

(10 mL, 30% in water) was slowly added. The mixture was stirred at this temperature for 1 h before it was carefully quenched with saturated aqueous $NaHCO_3$ (10 mL) and extracted with DCM (10 mL × 3). The combined organic phase was washed with 10% $Na_2S_2O_5$ (20 mL) and brine, dried (Na_2SO_4), filtered and concentrated. The residue was purified on column chromatography (hexanes/EtOAc = 97/3) and compound **46** was obtained as a white solid (237.0 mg, 45%). **^{1}H NMR (500 MHz, CDCl$_3$)** δ 7.19 (d, J = 7.7 Hz, 1H), 7.11 (s, 1H), 7.07 (d, J = 7.8 Hz, 1H), 6.92 (d, J = 12.7 Hz, 1H), 6.03 (d, J = 12.7 Hz, 1H), 3.22 (d, J = 10.3 Hz, 1H), 2.71 – 2.61 (m, 1H), 2.36 (s, 3H), 2.23 – 2.12 (m, 1H), 1.89 – 1.80 (m, 1H), 1.50 – 1.37 (m, 2H), 1.33 – 1.23 (m, 1H), 1.16 (s, 3H), 0.59 (d, J = 6.6 Hz, 3H), 0.27 (d, J = 6.3 Hz, 3H); **^{13}C NMR (126 MHz, CDCl$_3$)** δ 204.6, 142.4, 141.1, 140.4, 134.1, 133.9, 131.5, 128.0, 127.7, 58.9, 56.6, 51.7, 37.9, 30.7, 27.3, 24.2, 23.2, 21.3, 21.0; **IR (film)**: 2954, 2868, 1647, 1608, 1459, 840 cm^{-1}; **HRMS (APCI):** *m/z* Calc. for $C_{19}H_{25}O$ [M+H]$^+$: 269.1905, found: 269.1914.

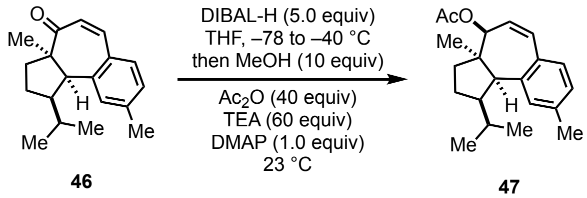

46

47

83%

Compound 47[8]: To a flame-dried vial were added compound **46** (17.0 mg, 0.0634 mmol, 1.0 equiv) and THF (1 mL). The mixture was cooled down to −78 °C and DIBAL-H (0.30 mL, 1 M in toluene, 0.3 mmol, 5 equiv) was added dropwise. The resulting mixture was warmed up to −40 °C and stirred for 30 min before it was quenched with MeOH (25 µL, 0.62 mmol, 10 equiv) and warmed up to room temperature. Triethylamine (0.53 mL, 3.8 mmol, 60 equiv), acetic anhydride (0.24 mL, 2.5 mmol, 40 equiv) and DMAP (7.7 mg, 0.063 mmol, 1.0 equiv) was subsequently added to the reaction mixture. The reaction was further stirred at room temperature for 7 h before it was quenched with saturated aqueous sodium bicarbonate (6 mL) and extracted with EtOAc (8 mL × 3). The combined organic phase was washed with brine (10 mL), dried (Na_2SO_4), filtered and concentrated. The residue was purified on column chromatography (hexanes/EtOAc = 30/1). Compound **47** was obtained as a white solid (16.4 mg, 83%). **^{1}H NMR (500 MHz, CDCl$_3$)** δ 7.03 – 6.94 (m, 3H), 6.45 (dd, J = 10.8, 2.2 Hz, 1H), 5.69 (dd, J = 10.7, 4.5 Hz, 1H), 5.12 – 5.03 (m, 1H), 3.07 (d, J = 10.6 Hz, 1H), 2.35 – 2.25 (m,

1H), 2.31 (s, 3H), 2.07 (s, 3H), 1.98 – 1.86 (m, 1H), 1.80 – 1.72 (m, 2H), 1.38 – 1.28 (m, 1H), 1.19 (s, 3H), 1.11 – 1.03 (m, 1H), 0.66 (d, J = 6.7 Hz, 3H), 0.37 (d, J = 6.3 Hz, 3H); **^{13}C NMR (126 MHz, $CDCl_3$, one peak covered by solvent peak)** δ 170.5, 139.9, 136.3, 134.9, 134.7, 133.1, 129.7, 128.8, 126.9, 64.1, 59.8, 54.8, 35.8, 34.8, 29.2, 28.1, 22.7, 22.0, 21.2, 21.1; **IR (film)**: 2952, 2867, 1736, 1367, 1233, 1024, 832 cm^{-1}; **HRMS (APCI):** *m/z* Calc. for $C_{21}H_{29}O_2$ $[M+H]^+$: 313.2168, found: 313.2163.

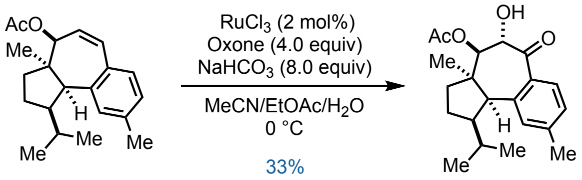

47 **48**

Compound 48 [9]: To a reaction vial were added oxone (89.8 mg, 0.146 mmol, 4.0 equiv), $NaHCO_3$ (24.9 mg, 0.296 mmol, 8 equiv) and water (0.05 mL). The resulting mixture was kept at room temperature for 5 min while CO_2 was generated vigorously. When gas release slowed down, MeCN (0.3 mL) and EtOAc (0.3 mL) was added. Compound **47** (11.4 mg, 0.0365 mmol, 1.0 equiv) was added to the suspension and the mixture was cooled down to 0 °C. To another vial were added $RuCl_3 \cdot xH_2O$ (1.8 mg, Ruthenium content 38-42%, 0.0074 mmol, 20 mol%), $NaHCO_3$ (24.9 mg, 0.296 mmol, 8 equiv), water (0.05 mL), MeCN (0.30 mL) and EtOAc (0.30 mL). Oxone (89.8 mg, 0.146 mmol, 4.0 equiv) was added to the mixture and the vial was gently shaken until the suspension turned bright yellow. The solution with freshly prepared Ru species (0.06 mL, containing 0.00074 mmol [Ru], 2 mol%) was carefully transferred to the reaction vial, and the resulting mixture was stirred at 0 °C for 90 min before it was quenched with aqueous $NaHSO_3$ (5%, 1 mL) and extracted with ether (3 mL × 3). The combined organic phase was washed with aqueous $NaHSO_3$ (10%, 3 mL × 2) and saturated aqueous sodium bicarbonate (3 mL × 2), dried (Na_2SO_4), filtered and concentrated. The residue was purified on column chromatography (hexanes/EtOAc = 8/1 to 6/1). Compound **48** was obtained as a colorless oil (4.2 mg, 33%). **^{1}H NMR (500 MHz, $CDCl_3$)** δ 7.63 (brs, 1H), 7.17 (d, J = 7.8 Hz, 1H), 7.06 (s, 1H), 4.85 (d, J = 10.0 Hz, 1H), 4.36 (d, J = 9.9 Hz, 1H), 3.36 (d, J = 10.0 Hz, 1H), 3.11 (brs, 1H), 2.38 (s, 3H), 2.36 – 2.25 (m, 1H), 2.18 – 1.98 (m, 1H), 2.10 (brs, 3H), 1.93 – 1.79 (m, 1H), 1.48 – 1.37 (m, 2H), 1.19 (s, 3H), 1.06 – 0.91 (m, 1H), 0.70 (d, J = 6.4 Hz, 3H), 0.36 (d, J = 6.1

Hz, 3H).; **^{13}C NMR (126 MHz, $CDCl_3$)** δ 202.7, 171.1, 143.3, 139.3, 134.7, 133.2, 129.8, 128.3, 128.1, 76.4 (covered by solvent peak), 75.1, 59.9, 53.7, 47.2, 35.2, 32.1, 29.9, 28.6, 22.6, 21.9, 21.5, 21.0; **IR (film)**: 3440, 2957, 2925, 1743, 1686, 1605, 1233, 1031 cm^{-1}; **HRMS (APCI):** *m/z* Calc. for $C_{21}H_{29}O_2$ $[M+H]^+$: 345.2066, found: 345.2061.

Table S1. ^{1}H NMR and ^{13}C NMR comparison of synthetic Hamigeran I and natural Hamigeran I.[12]

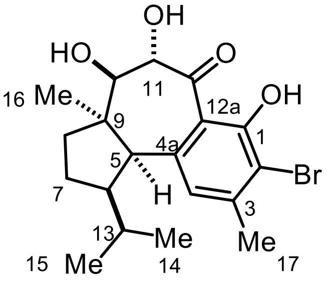

Hamigeran I

position	^{1}H		^{13}C		
	natural	synthesized	natural	synthesized	$\Delta_{nat\text{-}syn}$
1			160.9	164.8	0.1
1-OH	13.30	13.27			
2			113.6	113.4	0.2
3			148.2	148.0	0.2
4	6.77	6.77	128.4	128.2	0.2
4a			141.9	141.7	0.2
5	3.44	3.44	61.2	61.1	0.1
6	2.22	2.30 – 2.17	53.9	53.9	0
7	1.58	1.66 – 1.51	32.5	32.3	0.2
	1.92	1.99 – 1.87			
8	1.42	1.49 – 1.36	34.6	34.4	0.2
	2.57	2.64 – 2.53			
9			47.9	47.7	0.2
10	3.32	3.33	74.45**	74.3	0.2
11	4.59	4.59	74.43**	74.3	0.1
12			204.9	204.8	0.1
12a			116.3	116.2	0.1
13	1.10	1.15 – 1.04	29.6	29.5	0.1
14	0.77	0.78	22.1	22.0	0.1
15	0.20	0.21	23.5	23.4	0.1
16	1.25	1.25	30.1	30.0	0.1
17	2.45	2.45	24.0	23.9	0.1
10-OH	4.15	3.17			
11-OH	3.17	4.14*			

* Assigned by H-H COSY spectrum

** Interchangeable

Table S2. ^{1}H NMR and ^{13}C NMR comparison of synthetic debromohamigeran I and natural debromohamigeran I.[12]

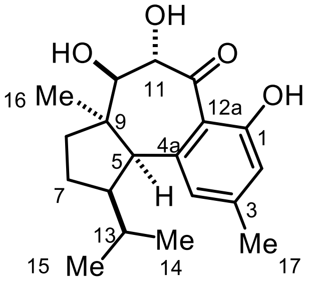

Debromohamigeran I

position	^{1}H		^{13}C		
	natural	synthesized	natural	synthesized	$\Delta_{nat\text{-}syn}$
1			165.0	164.9	0.1
1-OH	12.54	12.52			
2	6.77	6.77	118.1	117.9	0.2
3			143.1	143.0	0.1
4	6.64	6.64	127.9	127.8	0.1
4a			148.7	148.5	0.2
5	3.47	3.46	61.4	61.3	0.1
6	2.21	2.26 – 2.16	53.9	53.8	0.1
7	1.60	1.66 – 1.53	32.4	32.3	0.1
	1.91	1.97 – 1.86			
8	1.40	1.47 – 1.37	34.6	34.4	0.2
	2.59	2.61 – 2.54			
9			47.9	47.7	0.2
10	3.33	3.34	74.3	74.2	0.1
11	4.56	4.57	74.6	74.5	0.1
12			204.4	204.3	0.1
12a			115.5	115.4	0.1
13	1.12	1.18 – 1.08	29.7	29.6	0.1
14	0.76	0.77	23.3	23.2	0.1
15	0.18	0.19	22.2	22.0	0.2
16	1.24	1.25	30.2	30.0	0.2
17	2.32	2.32	22.0	21.8	0.2
10-OH	*	3.18			
11-OH	*	4.25			

* Not reported.

Table S3. ^{1}H NMR and ^{13}C NMR comparison of synthetic Hamigeran G and natural Hamigeran G.[12]

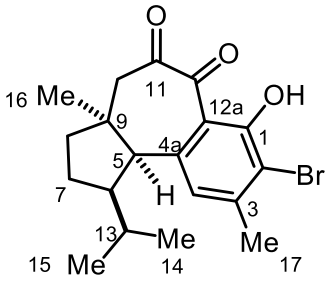

Hamigeran G

position	^{1}H		^{13}C		
	natural	synthesized	natural	synthesized	$\Delta_{nat\text{-}syn}$
1			160.4	160.3	0.1
1-OH	12.18	12.17			
2			113.2	113.1	0.1
3			148.3	148.1	0.2
4	6.78	6.78	128.1	128.0	0.1
4a			141.9	141.8	0.1
5	3.39	3.39	60.8	60.7	0.1
6	2.01	2.08 – 1.95	53.6	53.5	0.1
7	1.16	1.21 – 1.11	31.4	31.3	0.1
	1.74	1.78 – 1.71			
8	1.62	1.62	40.7	40.6	0.1
	1.98	2.08 – 1.95			
9			45.4	45.3	0.1
10	2.58	2.58	53.5	53.4	0.1
11	2.71	2.71	195.8	195.6	0.2
12			190.8	190.7	0.1
12a			118.6	118.5	0.1
13	0.84	0.90 – 0.80	29.9	29.8	0.1
14	0.69	0.69	22.2	22.0	0.2
15	0.25	0.25	22.4	22.2	0.2
16	1.29	1.29	31.9	31.8	0.1
17	2.48	2.48	24.2	24.0	0.2

Table S4. ^{1}H NMR and ^{13}C NMR comparison of synthetic Hamigeran C and natural Hamigeran C.[13]

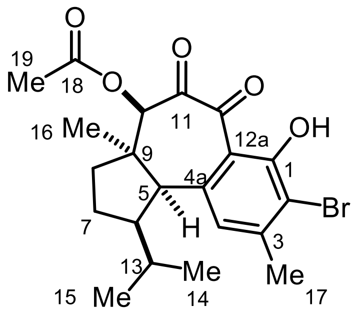

Hamigeran C

position	^{1}H		^{13}C		
	natural	synthesized	natural	synthesized	$\Delta_{nat-syn}$
1			160.4	160.4	0
1-OH	12.04	12.04			
2			113.6	113.6	0
3			148.8	148.8	0
4	6.80	6.80	128.2	128.3	-0.1
4a			139.7	139.7	0
5	3.49	3.49	59.8	59.8	0
6	2.02	2.08 – 1.97	53.2	53.2	0
7	1.05	1.11 – 1.01	31.6	31.6	0
	1.74	1.78 – 1.70			
8	2.30	2.34 – 2.27	34.8	34.8	0
	1.41	1.47 – 1.37			
9			47.6	47.6	0
10	5.08	5.08	81.6	81.7	-0.1
11			191.2	191.2	0
12			187.4	187.5	-0.1
12a			118.3	118.3	0
13	0.85	0.91 – 0.81	29.6	29.6	0
14	0.68	0.68	22.1	22.1	0
15	0.27	0.27	22.3	22.3	0
16	1.34	1.34	28.9	28.9	0
17	2.49	2.49	24.0	24.0	0
18			170.0	170.0	0
19	2.16	2.16	20.4	20.4	0

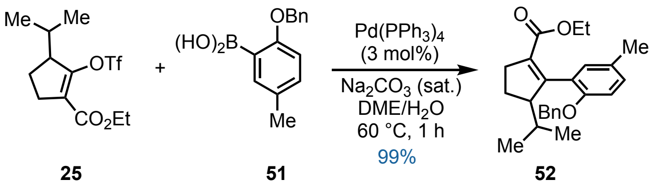

25 **51** **52**

Compound 52: To compound **25** (racemic, 0.720 g, 2.18 mmol, 1.0 equiv), boronic acid **51** (0.631 g, 2.62 mmol, 1.2 equiv) and $Pd(PPh_3)_4$ (75.3 mg, 0.0653 mmol, 3 mol%) in a reaction flask under argon was added DME (10 mL) and degassed saturated aqueous sodium carbonate (2 mL). The reaction was warmed up to 60 ºC and stirred for 1 h. Then the mixture was cooled down to room temperature and quenched with saturated aqueous ammonium chloride (20 mL). The residue was extracted with ether (20 mL × 3). The combined organic phase was washed with brine (40 mL), dried (Na_2SO_4), filtered and concentrated. The residue was purified by column chromatography (hexane/EtOAc = 95/5). Compound **52** was obtained as a colorless oil (0.817 g, 99%). **^{1}H NMR (500 MHz, CDCl$_3$)** δ 7.42 – 7.34 (m, 4H), 7.34 – 7.29 (m, 1H), 7.03 (dd, J = 8.3, 1.8 Hz, 1H), 6.93 (d, J = 1.8 Hz, 1H), 6.83 (d, J = 8.3 Hz, 1H), 5.05 (s, 2H), 4.09 – 3.98 (m, 1H), 3.98 – 3.87 (m, 1H), 3.38 – 3.26 (m, 1H), 2.86 – 2.74 (m, 1H), 2.74 – 2.64 (m, 1H), 2.30 (s, 3H), 2.04 – 1.90 (m, 1H), 1.88 – 1.71 (m, 2H), 1.01 (t, J = 7.2 Hz, 3H), 0.89 (d, J = 6.9 Hz, 3H), 0.75 (d, J = 6.8 Hz, 3H); **^{13}C NMR (126 MHz, CDCl$_3$)** δ 166.0, 153.4, 137.6, 131.2, 130.2, 129.3, 128.8, 128.4, 127.5, 126.9, 126.8, 112.2, 77.3, 77.0, 76.8, 70.3, 59.6, 56.5, 33.4, 29.0, 22.0, 21.7, 20.5, 16.2, 13.9; **HRMS (ESI):** *m/z* Calc. for $C_{25}H_{31}O_3$ [M+H]$^+$: 379.2273, found: 379.2270.

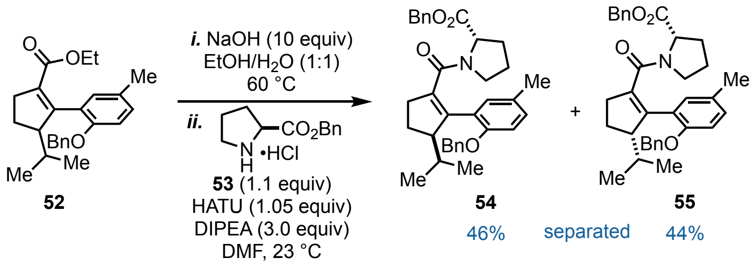

Compounds 54 and **55**: To compound **52** (0.817 g, 2.16 mmol, 1.0 equiv) in EtOH (2 mL) was added sodium hydroxide (0.86 g, 22 mmol, 10 equiv) in water (3 mL). The mixture was warmed up to 60 ºC and stirred overnight before it was cooled down to room temperature and acidified with HCl (3 M) to pH = 1. The resulting residue was extracted with ether (10 mL × 3). The combined organic phase was washed with brine (20 mL), dried (Na_2SO_4), filtered, concentrated, and used in the next step without further purification.

To the crude carboxylic acid was added (*L*)-proline derivative **53** (0.574 g, 2.37 mmol, 1.10 equiv), HATU (0.862 g, 2.27 mmol, 1.05 equiv) and DMF (12 mL). To the mixture was slowly added DIPEA (1.1 mL, 6.5 mmol, 3.0 equiv) and the reaction was stirred at room temperature for 1 h before it was quenched with water (15 mL) and extracted with ether (20 mL × 3). The combined organic phase was washed with brine (20 mL × 3), dried (Na_2SO_4), filtered and concentrated. The residue was purified by repeated column chromatography (hexane/EtOAc = 5/1 to 2/1). Compounds **54** and **55** were obtained as colorless oil (compound **54**: 530.4 mg, 46%; compound **55**: 511.8 mg, 44%).

54: **^{1}H NMR (500 MHz, $CDCl_3$)** δ 7.47 – 7.29 (m, 10H), 7.07 – 6.97 (m, 2H), 6.80 (d, *J* = 8.2 Hz, 1H), 5.25 – 5.03 (m, 4H), 4.32 (dd, *J* = 8.6, 3.9 Hz, 1H), 3.58 – 3.51 (m, 1H), 3.42 – 3.30 (m, 1H), 2.95 – 2.78 (m, 2H), 2.63 – 2.50 (m, 1H), 2.24 (s, 3H), 2.01 – 1.67 (m, 6H), 1.35 – 1.22 (m, 2H), 0.86 (d, *J* = 6.9 Hz, 3H), 0.70 (d, *J* = 6.9 Hz, 3H); **^{13}C NMR (126 MHz, $CDCl_3$)** δ 172.2, 168.8, 153.4, 144.1, 137.3, 135.9, 135.7, 131.5, 130.1, 129.2, 128.5, 128.5, 128.5, 128.1, 128.1, 127.8, 127.1, 125.8, 111.6, 70.1, 66.6, 58.3, 53.6, 47.0, 34.3, 29.4, 29.3, 24.4, 22.8, 21.4, 20.4, 16.3; **HRMS (ESI):** *m/z* Calc. for $C_{35}H_{40}NO_4$ $[M+H]^+$: 538.2957, found: 538.2954. $[\alpha]_D^{25}$ = –116.2 (c 1.0, $CHCl_3$).

55 (two conformers): **^{1}H NMR (500 MHz, $CDCl_3$)** δ 7.44 – 7.20 (m, 10H), 7.09 – 6.97 (m, 2H), 6.81 (s, 0.5H), 6.80 (s, 0.5H), 5.26 – 5.00 (m, 4H), 4.49 (t, *J* = 7.3 Hz, 0.5H), 3.99 (dd, *J* = 8.2, 3.2 Hz, 0.5H), 3.66 – 3.51 (m, 1H), 3.48 – 3.38 (m, 1H), 3.38 – 3.26 (m, 0.5H), 2.97 – 2.87 (m, 0.5H), 2.81 – 2.64 (m, 1H), 2.62 – 2.52 (m, 0.5H), 2.28 (s, 1.5H), 2.23 (s, 1.5H), 2.18 – 2.09 (m, 0.5H), 2.00 – 1.90 (m, 1H), 1.90 – 1.79 (m, 1H), 1.79 – 1.57 (m, 4H), 1.57 – 1.48 (m, 0.5H), 1.48 – 1.40 (m, 0.5H), 0.87 (d, *J* = 6.8 Hz, 1.5H), 0.84 (d, *J* = 6.9 Hz, 1.5H), 0.71 (d, *J* = 6.8 Hz, 1.5H), 0.67 (d, *J* = 6.9 Hz, 1.5H); **^{13}C NMR (126 MHz, $CDCl_3$)** δ 172.2, 171.7, 169.4, 168.6, 153.2, 153.2, 145.8, 143.5, 137.5, 137.4, 136.6, 136.1, 135.7, 134.9, 132.3, 131.5, 130.6, 130.0, 129.4, 129.2, 128.5, 128.5, 128.3, 128.1, 128.0, 127.9, 127.8, 127.8, 127.1, 125.5, 125.5, 111.7, 111.4, 77.3, 77.1, 76.8, 70.1, 66.7, 66.3, 59.2, 58.3, 54.1, 53.4, 47.3, 45.8, 34.7, 34.0, 30.4, 29.4, 29.3, 29.2, 25.2, 22.8, 22.6, 21.5, 21.4, 20.4, 20.3, 16.4, 16.2; **HRMS (ESI):** *m/z* Calc. for $C_{35}H_{40}NO_4$ $[M+H]^+$: 538.2957, found: 538.2953. $[\alpha]_D^{25}$ = 29.2 (c 1.0, $CHCl_3$).

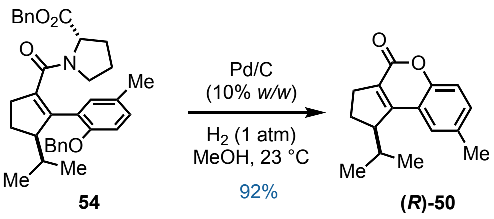

Compound (*R*)-50: To compound **54** (197.3 mg, 0.367 mmol, 1.0 equiv) and Pd/C (200 mg, 10% w/w, 54 mg Pd/mmol) was carefully added MeOH (3 mL) under argon. The resulting suspension was bubbled with H_2 (1 atm, balloon) for 20 min and then stirred under H_2 atmosphere for 1 day at room temperature before it was filtered through celite and concentrated. The residue was purified by column chromatography (hexane/DCM/EtOAc = 45/15/2). **(*R*)-50** was obtained as a white solid (81.6 mg, 92%). The characterization data was in accordance with reported racemic one.[1] $[\alpha]_D^{25}$ = 85.3 (c 1.0, $CHCl_3$).

Crystal Structure of **(*R*)-50:**

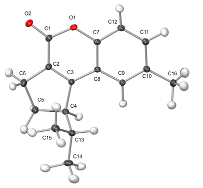

Experimental. Single colorless crystals of **(*R*)-50** were recrystallized from hexane. A suitable crystal with dimensions 0.2 × 0.15 × 0.06 mm^3 was selected and mounted on a loop with paratone on a XtaLAB Synergy S diffractometer. The crystal was kept at a steady T = 100(1) K during data collection. The structure was solved with the ShelXT 2018/2 (Sheldrick, 2018) solution program using dual methods and by using Olex2 1.5-alpha (Dolomanov et al., 2009) as the graphical interface. The model was refined with olex2.refine 1.5-alpha (Bourhis et al., 2015) using full matrix least squares minimisation on $\boldsymbol{F}^2$.

Crystal Data. $C_{16}H_{18}O_2$, M_r = 242.320, monoclinic, $P2_1$ (No. 4), a = 7.9321(6) Å, b = 6.5815(6) Å, c = 12.8255(13) Å, b = 96.180(8)°, a = g = 90°, V = 665.67(10) $Å^3$, T = 100(1) K, Z = 2, Z' = 1, m(Cu K_a) = 0.618, 7910 reflections measured, 2239 unique (R_{int} = 0.0761) which were used in all calculations. The final wR_2 was 0.1038 (all data) and R_1 was 0.0445 (I≥2 s(I)). The Flack parameter was refined to 0.2(3). Determination of absolute structure using Bayesian statistics on Bijvoet differences using the Olex2 results in 0.2(3). The chiral atoms in this

structure are: C4(R). Note: The Flack parameter is used to determine chirality of the crystal studied, the value should be near 0, a value of 1 means that the stereochemistry is wrong and the model should be inverted. A value of 0.5 means that the crystal consists of a racemic mixture of the two enantiomers.

**Compound**	**(*R*)-50**<br>LL-E-JB-20230801
Formula	$C_{16}H_{18}O_2$
$D_{calc.}$/ g cm^{-3}	1.209
m/mm^{-1}	0.618
Formula Weight	242.320
Colour	colourless
Shape	prism-shaped
Size/mm^3	0.20×0.15×0.06
T/K	100(1)
Crystal System	monoclinic
Flack Parameter	0.2(3)
Hooft Parameter	0.2(3)
Space Group	$P2_1$
a/Å	7.9321(6)
b/Å	6.5815(6)
c/Å	12.8255(13)
a/°	90
b/°	96.180(8)
g/°	90
V/Å^3	665.67(10)
Z	2
Z'	1
Wavelength/Å	1.54184
Radiation type	Cu K_a
Q_{min}/°	3.47
Q_{max}/°	65.08
Measured Refl's.	7910
Indep't Refl's	2239
Refl's I≥2 *s*(I)	1893
R_{int}	0.0761
Parameters	325
Restraints	325
Largest Peak	0.1843
Deepest Hole	-0.1959
GooF	1.0533
wR_2 (all data)	0.1038
wR_2	0.0984
R_1 (all data)	0.0538
R_1	0.0445

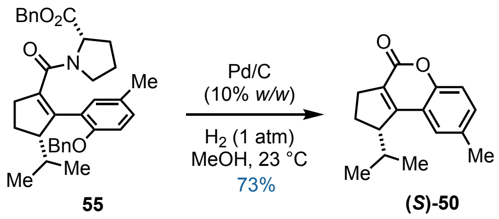

Compound (*S*)-50: To compound **55** (54.8 mg, 0.102 mmol, 1.0 equiv) and Pd/C (54 mg, 10% w/w, 54 mg Pd/mmol) was carefully added MeOH (1 mL) under argon. The resulting suspension was bubbled with H_2 (1 atm, balloon) for 20 min and then stirred under H_2 atmosphere for 1 day at room temperature before it was filtered through celite and concentrated. The residue was purified by column chromatography (hexane/DCM/EtOAc = 45/15/2). **(*S*)-50** was obtained as a white solid (18.0 mg, 73%). The characterization data was in accordance with reported racemic one.[1] $[\alpha]_D^{25}$ = –75.8 (c 1.0, $CHCl_3$).

Part 2. References

[1] Jiang, B.; Dai, M. 11-Step and Scalable Total Synthesis of Hamigeran M Enabled by Five C–H Functionalizations. *J. Am. Chem. Soc.* **2021**, *143*, 20084–20089.

[2] Kingsbury, J. S.; Corey, E. J. Enantioselective Total Synthesis of Isoedunol and β-Araneosene Featuring Unconventional Strategy and Methodology. *J. Am. Chem. Soc.* **2005**, *127*, 13813–13815.

[3] Corey, E. J.; Rao, S. A.; Noe, M. C. Catalytic Diastereoselective Synthesis of Cis-1,2-Disubstituted Cyclopropanols from Esters Using a Vicinal Dicarbanion Equivalent. *J. Am. Chem. Soc.* **1994**, *116*, 9345–9346.

[4] Ydhyam, S.; Cha, J.-K. Construction of Seven-Membered Carbocycles via Cyclopropanols. *Org. Lett.* **2015**, *17*, 5820–5823.

[5] Dragan, A.; Kubczyk, T. M.; Rowley, J. H.; Sproules, S.; Tomkinson, N. C. O. Arene Oxidation with Malonoyl Peroxides. *Org. Lett.* **2015**, *17*, 2618–2621.

[6] Peterson, G. A.; Kunng, F.-A.; McCallum, J. S.; Wulffe, W. D.; Palladium catalyzed reduction of aryl triflates-utilization in thesynthesis of angelicin, olivin and chromomycinone from phenolsproduced in the benzannulation reaction of chromium carbenecomplexes. *Tetrahedron Lett.* **1987**, *28*, 1381–1384.

[7] Mukaiyama, T.; Matsuo, J.; Kitagawa, H. A New and One-Pot Synthesis of α,β-Unsaturated Ketones by Dehydrogenation of Various Ketones with *N-tert*-Butyl Phenylsulfinimidoyl Chloride. *Chem. Lett.* **2000**, 1250–1251.

[8] Wilde, N. C.; Isomura, M.; Mendoza, A.; Baran, P. S. Two-Phase Synthesis of (−)-Taxuyunnanine D. *J. Am. Chem. Soc.* **2014**, *136*, 4909–4912.

[9] Plietker, B. RuO_4-Catalyzed Ketohydroxylation of Olefins. *J. Org. Chem.* **2003**, *68*, 7123–7125.

[10] Ono, M.; Ito, I. N-methyl-2-dimethylaminoacetohydroxamic acid as a new reagent for the selective cleavage of active esters under neutral conditions. *Tetrahedron Lett.* **1989**, *30*, 207–210.

[11] Li, X.; Xue, D.; Wang, C.; Gao, S. Total Synthesis of the Hamigerans. *Angew. Chem. Int. Ed.* **2016**, *55*, 9942–9946.

[12] Singh, A. J.; Dattelbaum, J. D.; Field, J. J.; Smart, Z.; Woolly, E. F.; Barber, J. M.; Heathcott, R.; Miller, J. H.; Northcote, P. T. Structurally diverse hamigerans from the New Zealand marine sponge Hamigera tarangaensis: NMR-directed isolation, structure elucidation and antifungal activity. *Org. Biomol. Chem*. **2013**, *11*, 8041–8051.

[13] Wellington, K. D.; Cambie, R. C.; Rutledge, P. S.; Bergquist, P. R. Chemistry of Sponges. 19. Novel Bioactive Metabolites from Hamigera tarangaensis. *J. Nat. Prod.* **2000**, *63*, 79–85.

Part 3. ^{1}H and ^{13}C NMR spectra

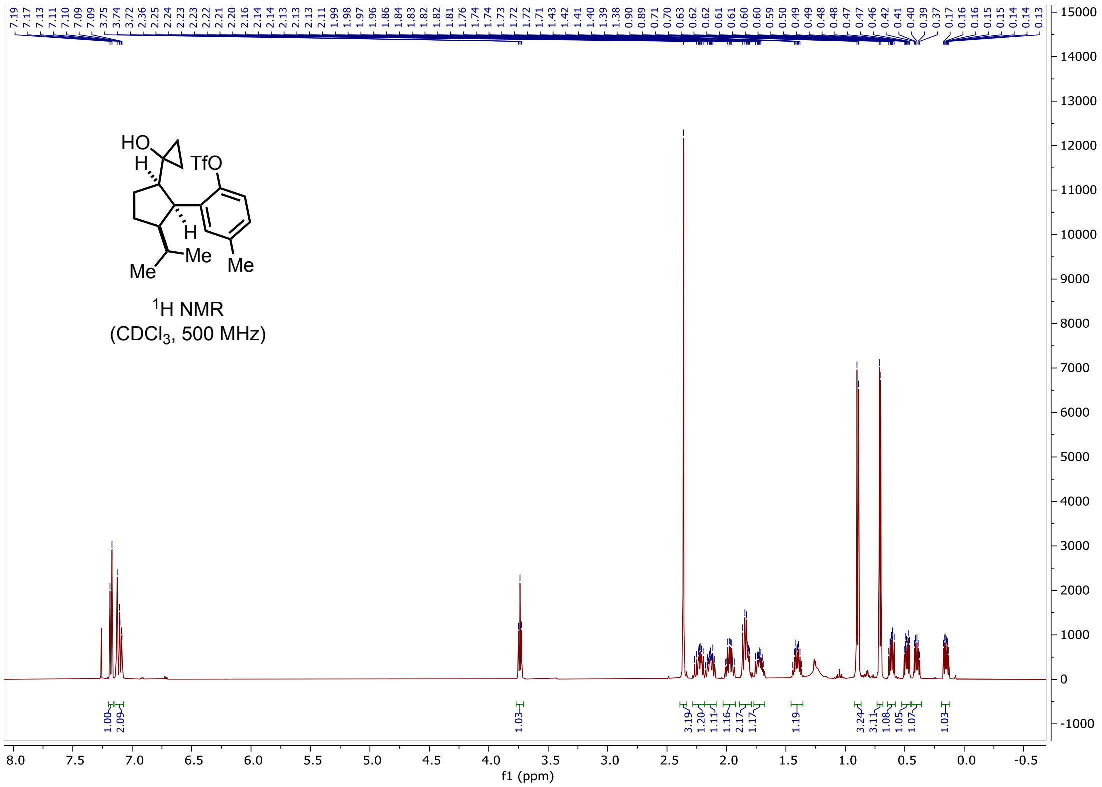

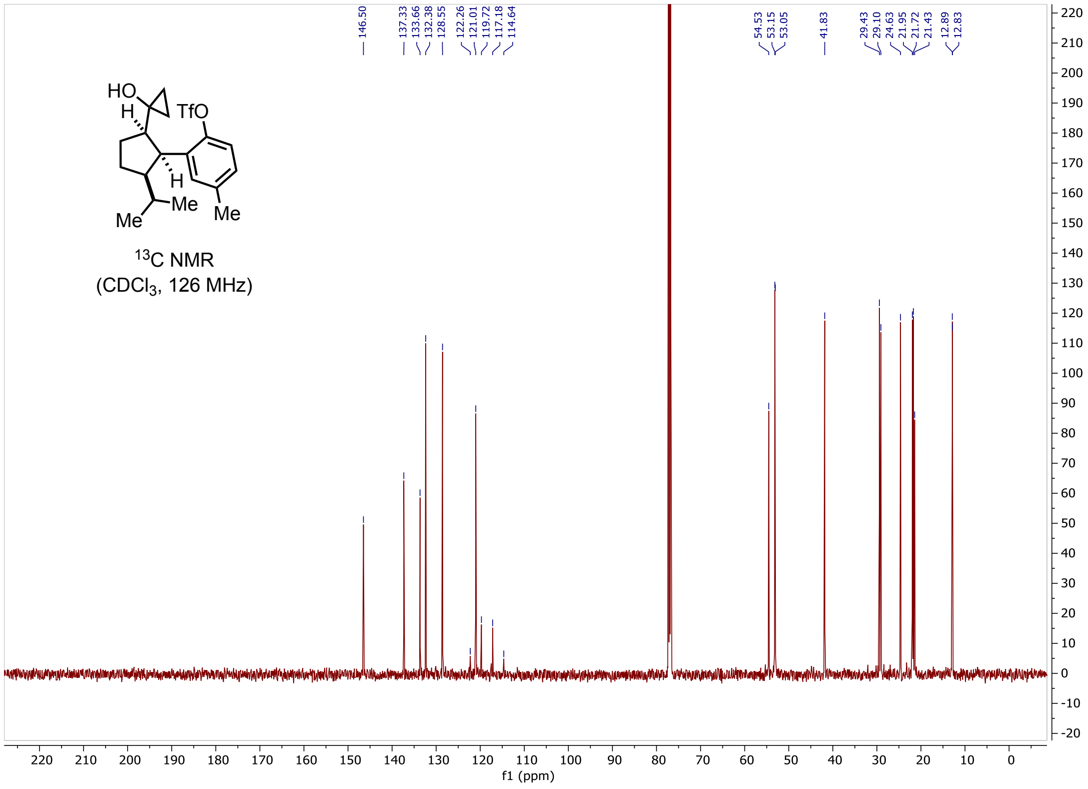

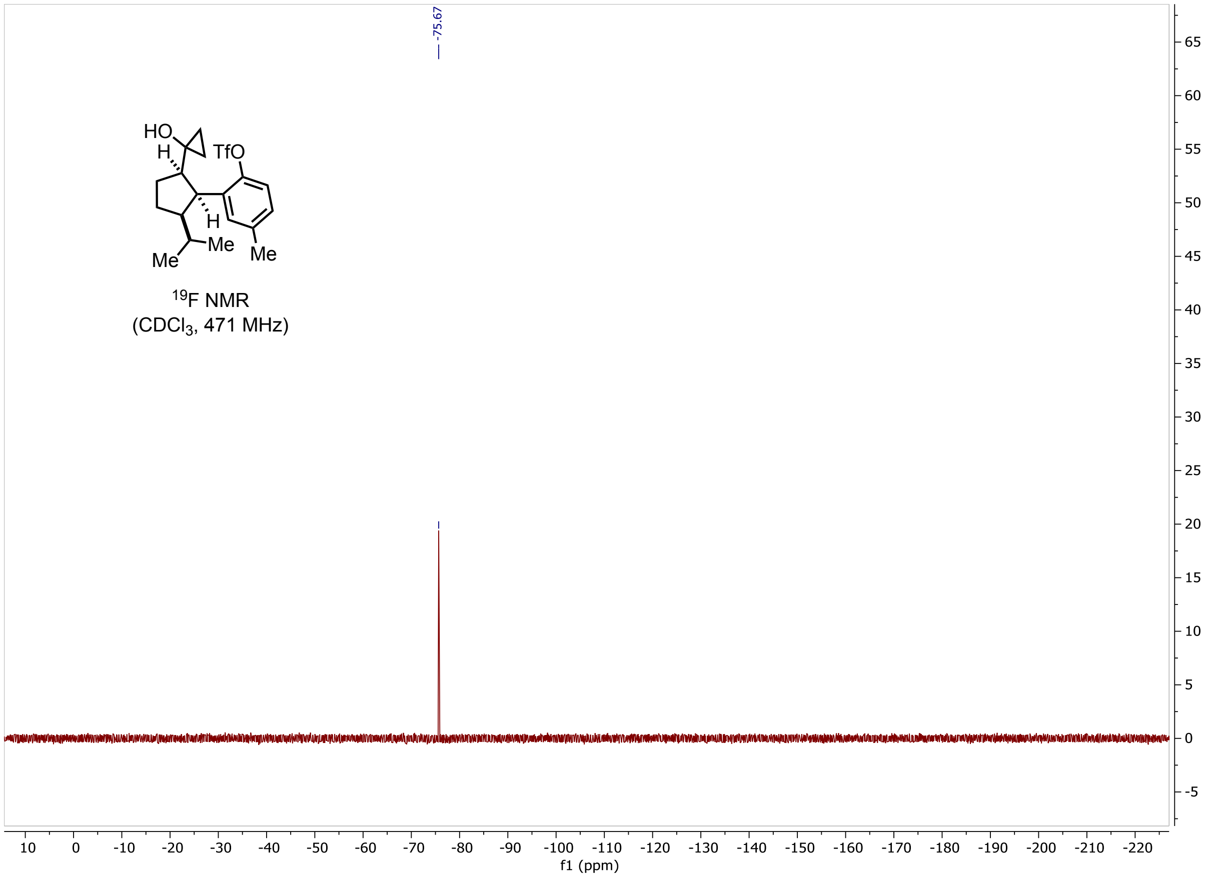

HO
H
TfO
H
Me
Me
Me
^{19}F NMR
($CDCl_3$, 471 MHz)
-75.67
f1 (ppm)

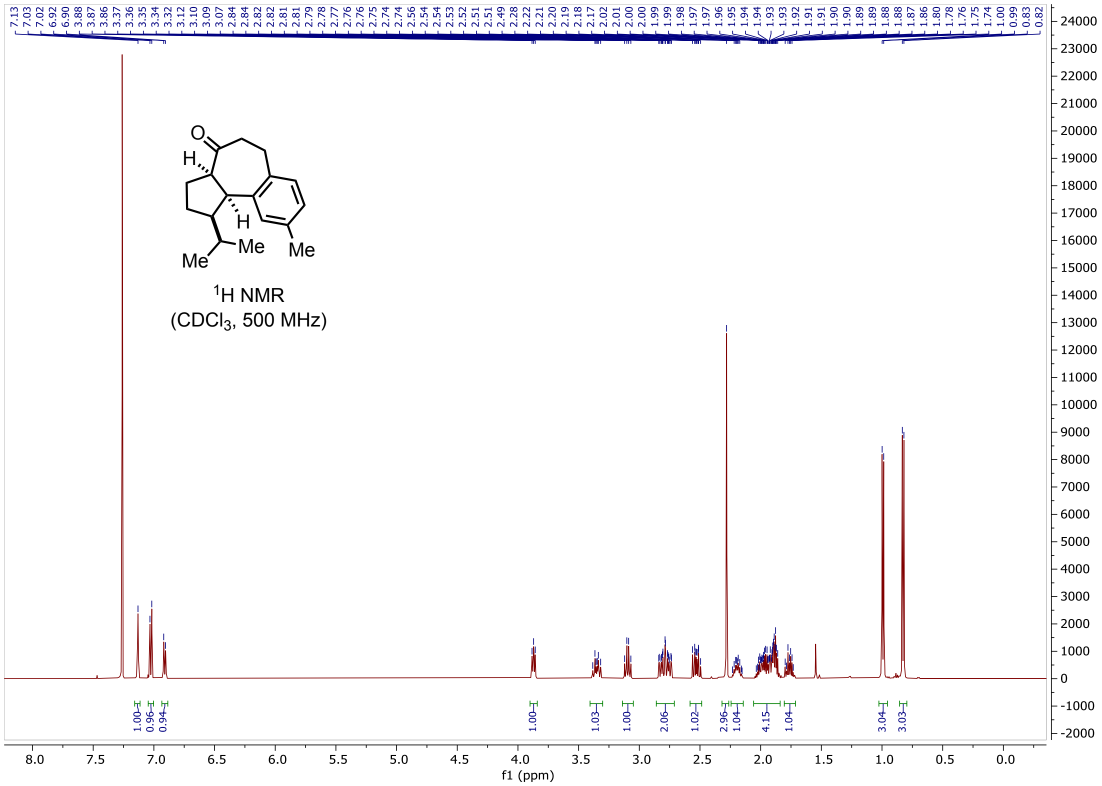
7.13 7.03 7.02 6.92 6.90 3.88 3.87 3.86 3.37 3.36 3.35 3.34 3.32 3.12 3.10 3.09 3.07 2.84 2.84 2.82 2.81 2.81 2.79 2.78 2.77 2.76 2.75 2.74 2.74 2.56 2.54 2.54 2.53 2.52 2.51 2.51 2.49 2.28 2.22 2.21 2.20 2.19 2.18 2.17 2.02 2.01 2.00 2.00 1.99 1.99 1.98 1.97 1.97 1.96 1.95 1.94 1.94 1.93 1.93 1.93 1.92 1.91 1.91 1.90 1.90 1.89 1.89 1.88 1.88 1.87 1.86 1.80 1.78 1.76 1.75 1.74 1.00 0.99 0.83 0.82
O
H
H
Me
Me
Me
^{1}H NMR
(CDCl$_3$, 500 MHz)
1.00 0.96 0.94 1.00 1.03 1.00 2.06 1.02 2.96 1.04 4.15 1.04 3.04 3.03
f1 (ppm)

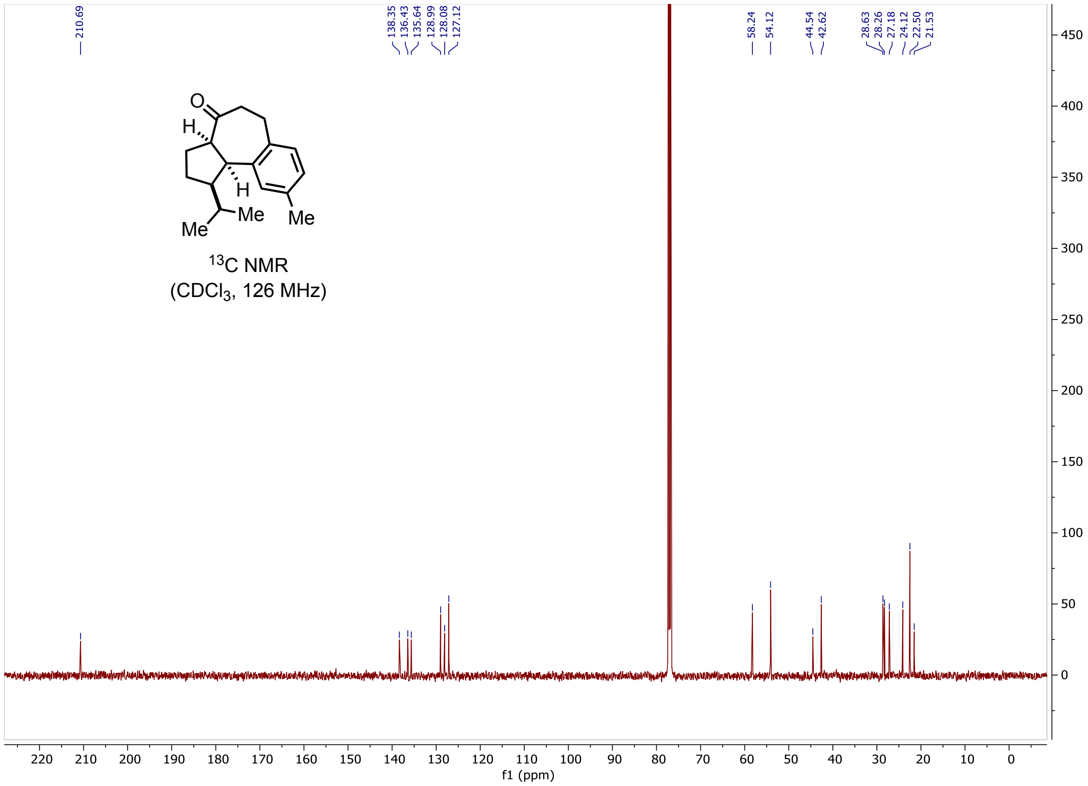
210.69
138.35 136.43 135.64 128.99 128.08 127.12
58.24 54.12 44.54 42.62 28.63 28.26 27.18 24.12 22.50 21.53
O
H
H
Me
Me
Me
^{13}C NMR
(CDCl$_3$, 126 MHz)
f1 (ppm)

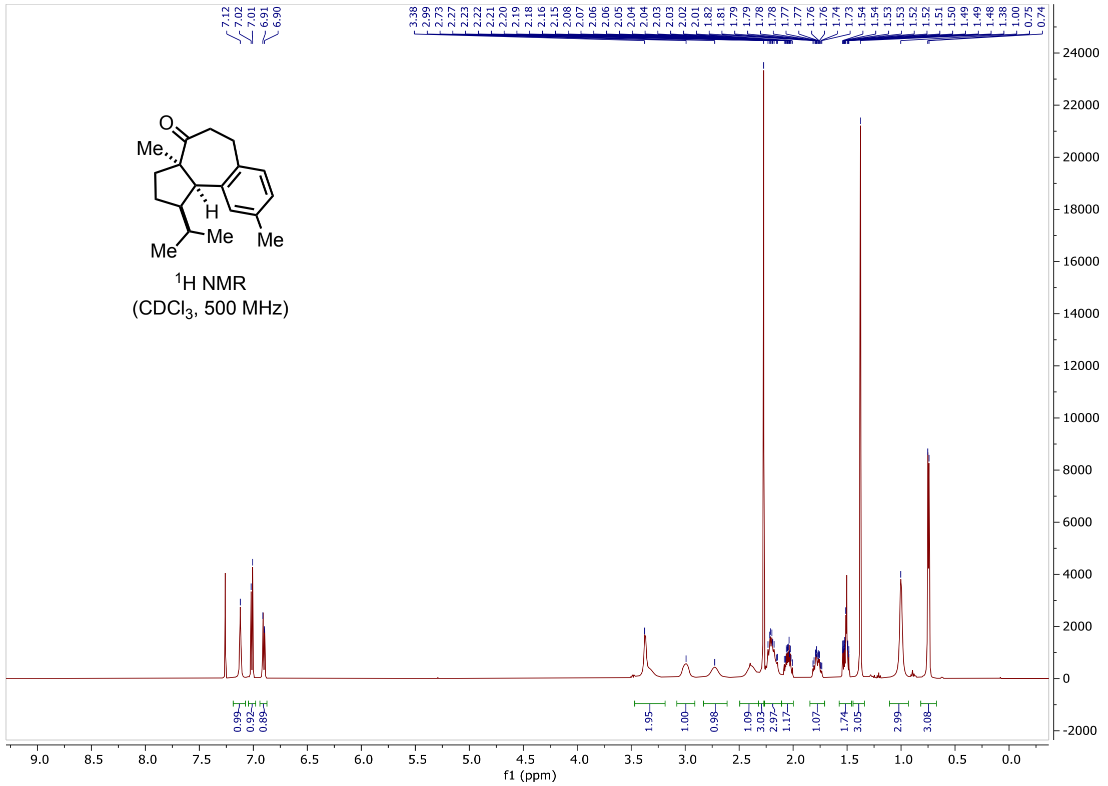
7.12
7.02
7.01
6.91
6.90
3.38
2.99
2.73
2.27
2.23
2.22
2.21
2.20
2.19
2.18
2.16
2.15
2.08
2.07
2.06
2.06
2.05
2.04
2.04
2.03
2.03
2.02
2.01
1.82
1.81
1.79
1.78
1.78
1.77
1.77
1.76
1.76
1.74
1.73
1.54
1.54
1.53
1.53
1.52
1.52
1.51
1.50
1.49
1.49
1.48
1.38
1.00
0.75
0.74
O
Me
H
Me
Me
Me
^{1}H NMR
($CDCl_3$, 500 MHz)
0.99
0.92
0.89
1.95
1.00
0.98
1.09
3.03
2.97
1.17
1.07
1.74
3.05
2.99
3.08
f1 (ppm)

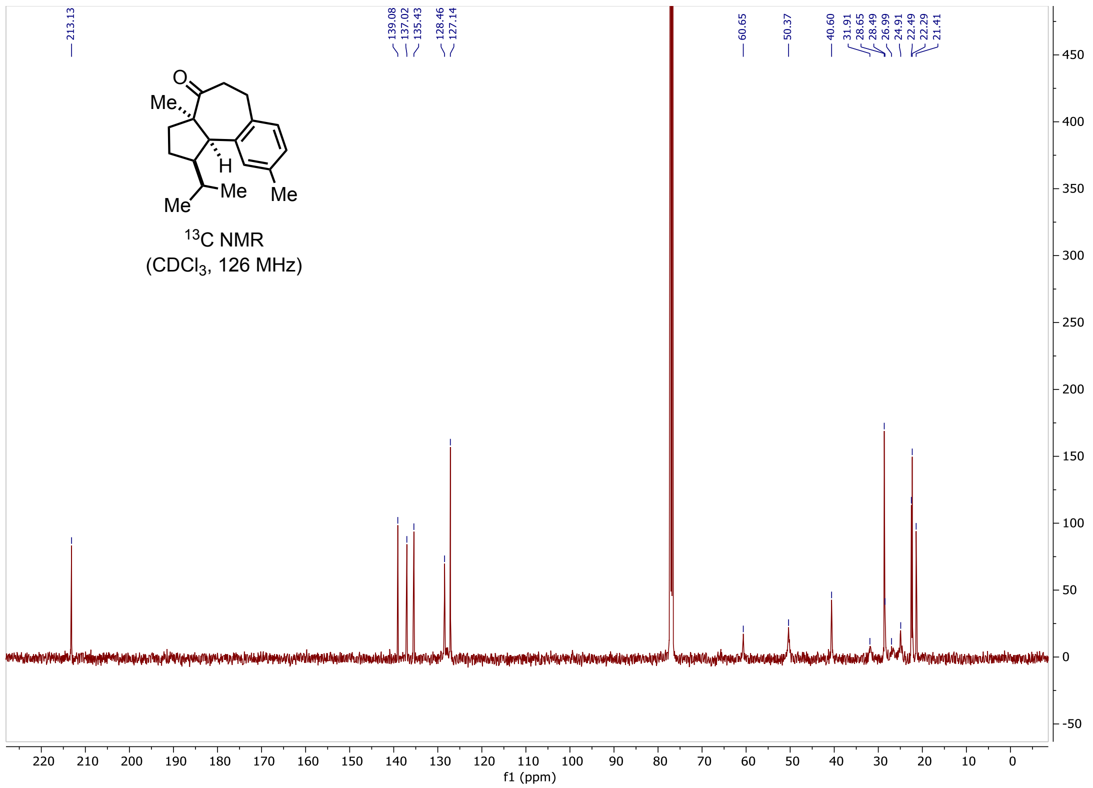
213.13
139.08
137.02
135.43
128.46
127.14
60.65
50.37
40.60
31.91
28.65
28.49
26.99
24.91
22.49
22.29
21.41
O
Me
H
Me
Me
Me
^{13}C NMR
($CDCl_3$, 126 MHz)
f1 (ppm)

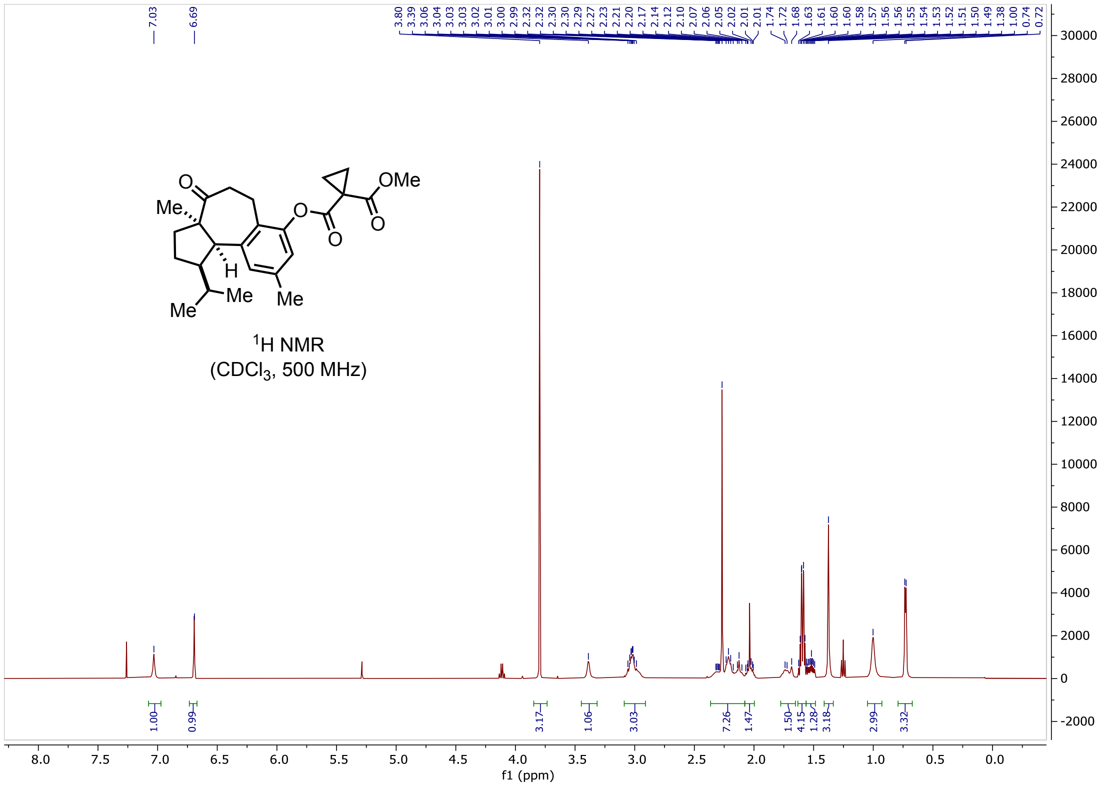
O
OMe
Me
H
Me
Me
Me
^{1}H NMR
(CDCl$_3$, 500 MHz)
7.03
6.69
3.80
3.39
3.06
3.04
3.03
3.03
3.02
3.01
3.00
2.99
2.32
2.32
2.30
2.30
2.29
2.27
2.23
2.21
2.20
2.17
2.14
2.12
2.10
2.07
2.06
2.05
2.02
2.01
2.01
1.74
1.72
1.68
1.63
1.61
1.60
1.60
1.58
1.57
1.56
1.56
1.55
1.54
1.53
1.52
1.51
1.50
1.49
1.38
1.00
0.74
0.72
1.00
0.99
3.17
1.06
3.03
7.26
1.47
1.50
4.15
1.28
3.18
2.99
3.32
f1 (ppm)

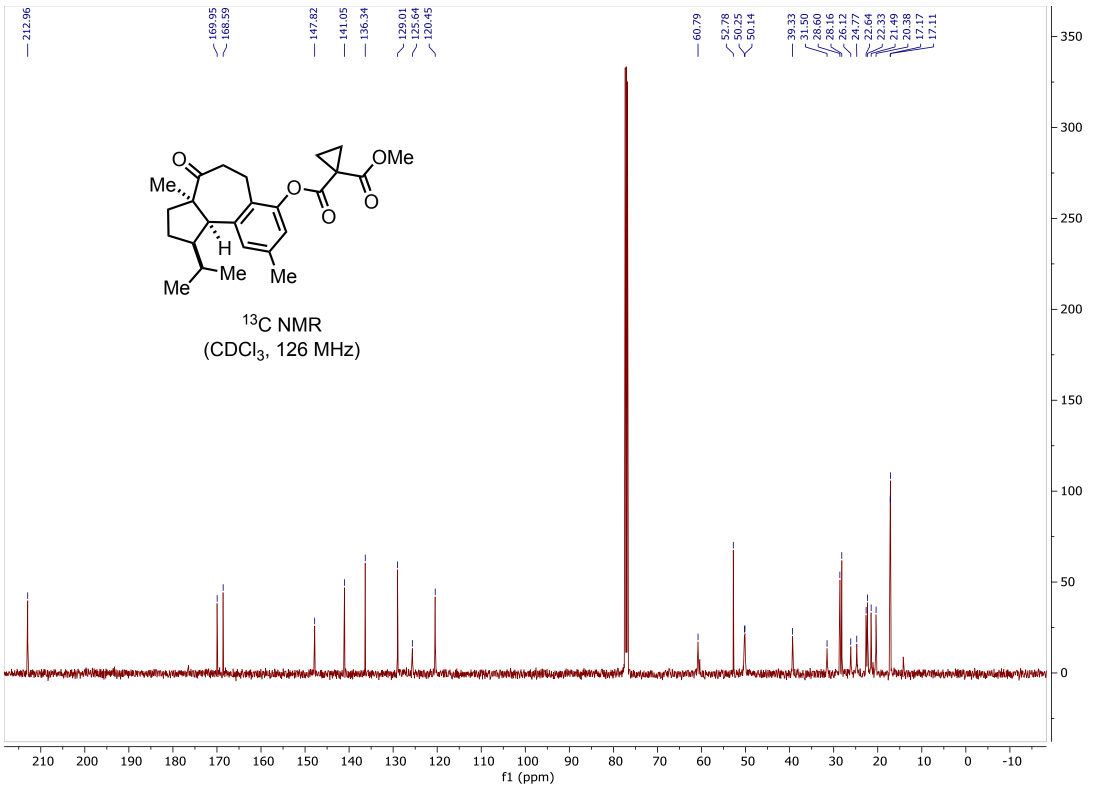
O
OMe
Me
H
Me
Me
Me
^{13}C NMR
(CDCl$_3$, 126 MHz)
212.96
169.95
168.59
147.82
141.05
136.34
129.01
125.64
120.45
60.79
52.78
50.25
50.14
39.33
31.50
28.60
28.16
26.12
24.77
22.64
22.33
21.49
20.38
17.17
17.11
f1 (ppm)

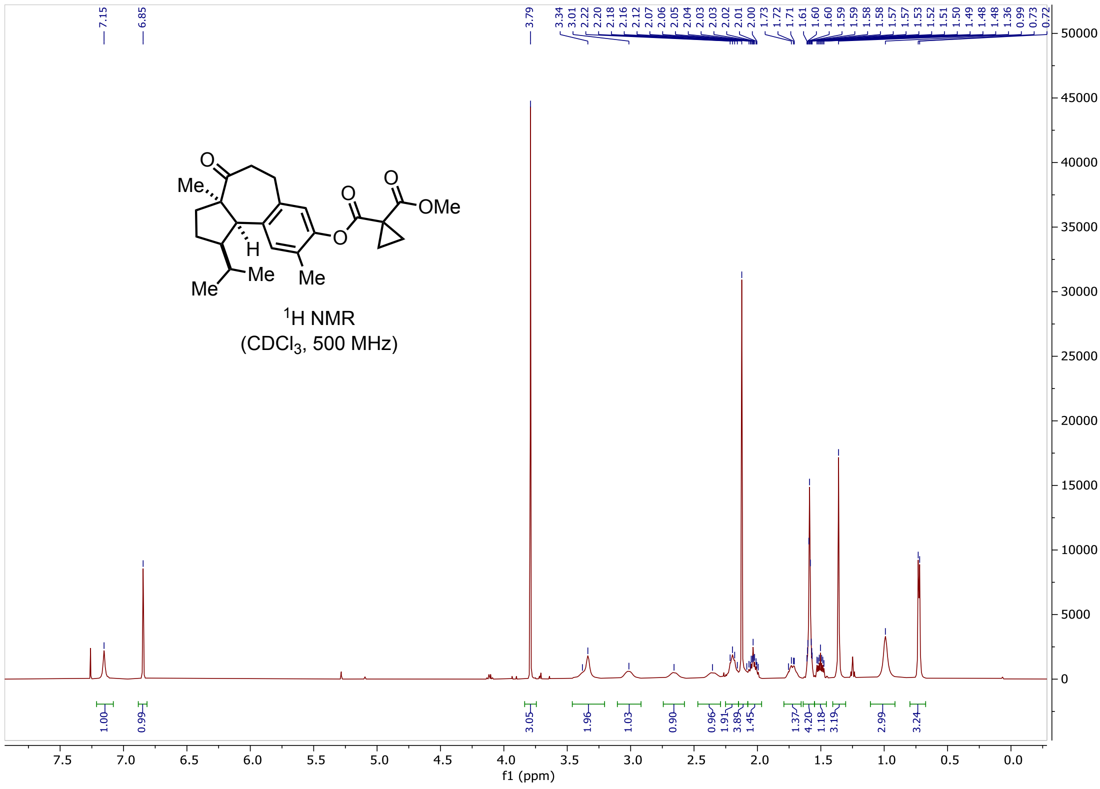

Me
H
Me
Me
Me
O
O
O
O
OMe
^{1}H NMR
(CDCl$_3$, 500 MHz)
f1 (ppm)

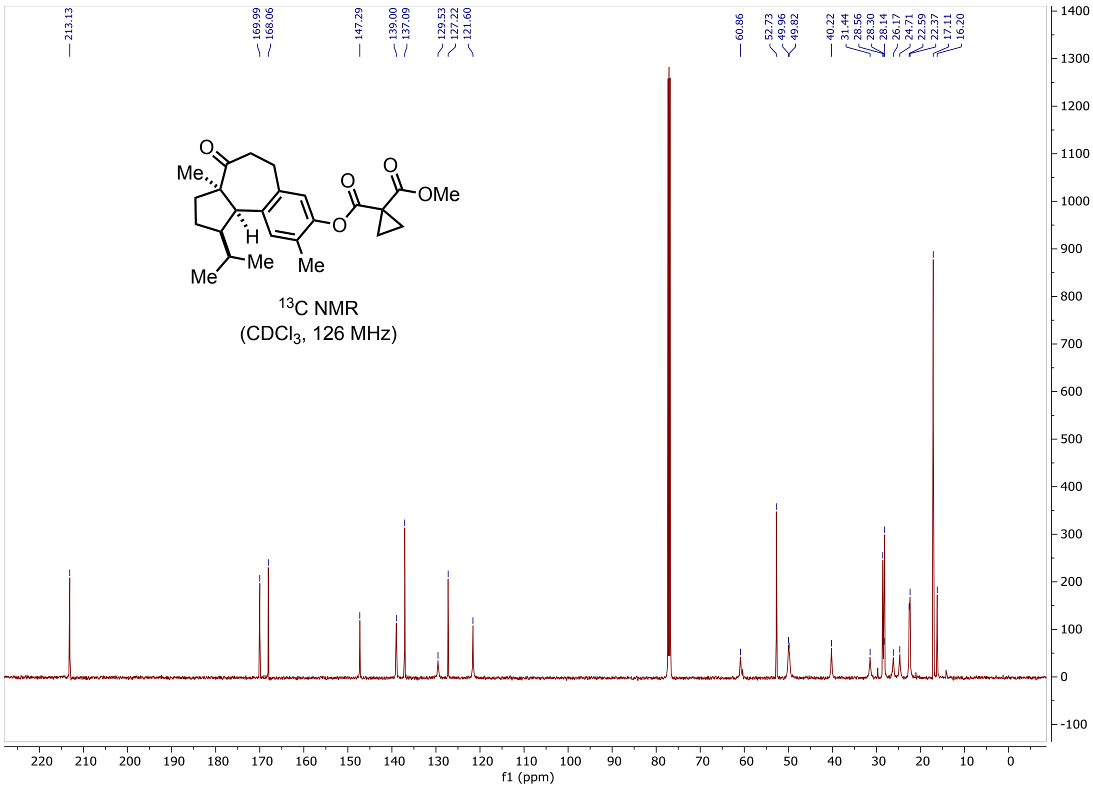

Me
H
Me
Me
Me
O
O
O
O
OMe
^{13}C NMR
(CDCl$_3$, 126 MHz)
f1 (ppm)

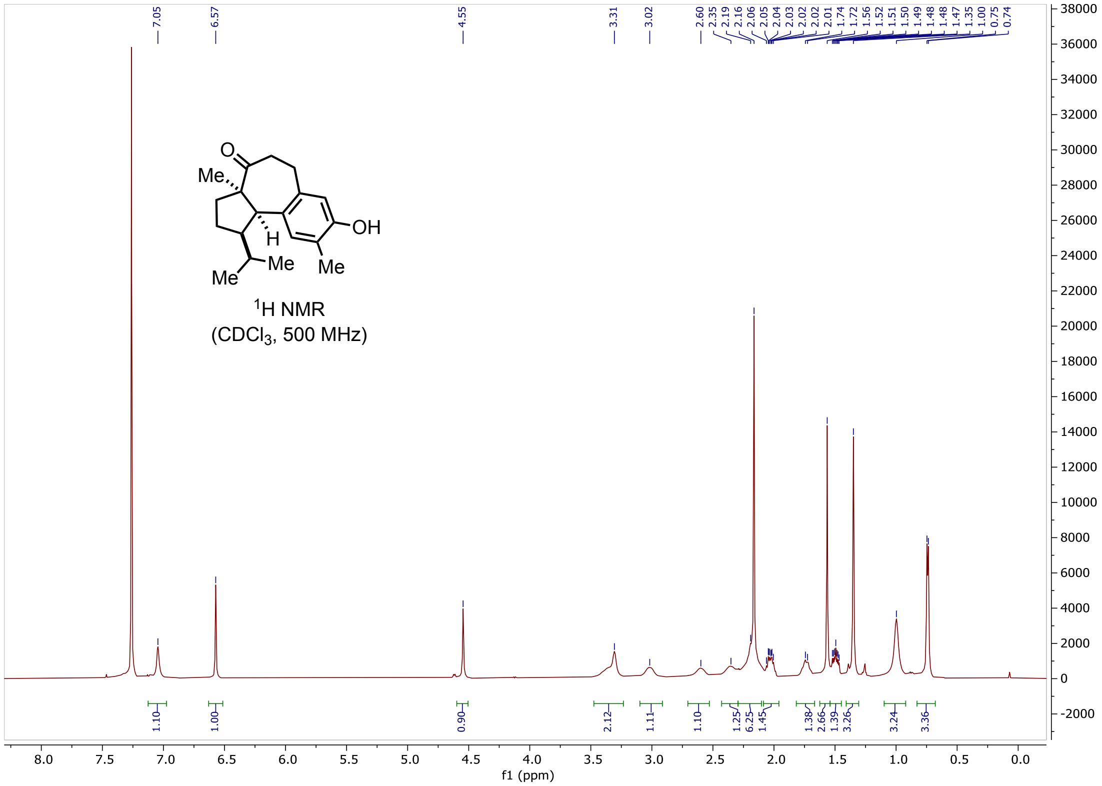

Me
O
OH
H
Me
Me
Me
^{1}H NMR
(CDCl$_3$, 500 MHz)
f1 (ppm)

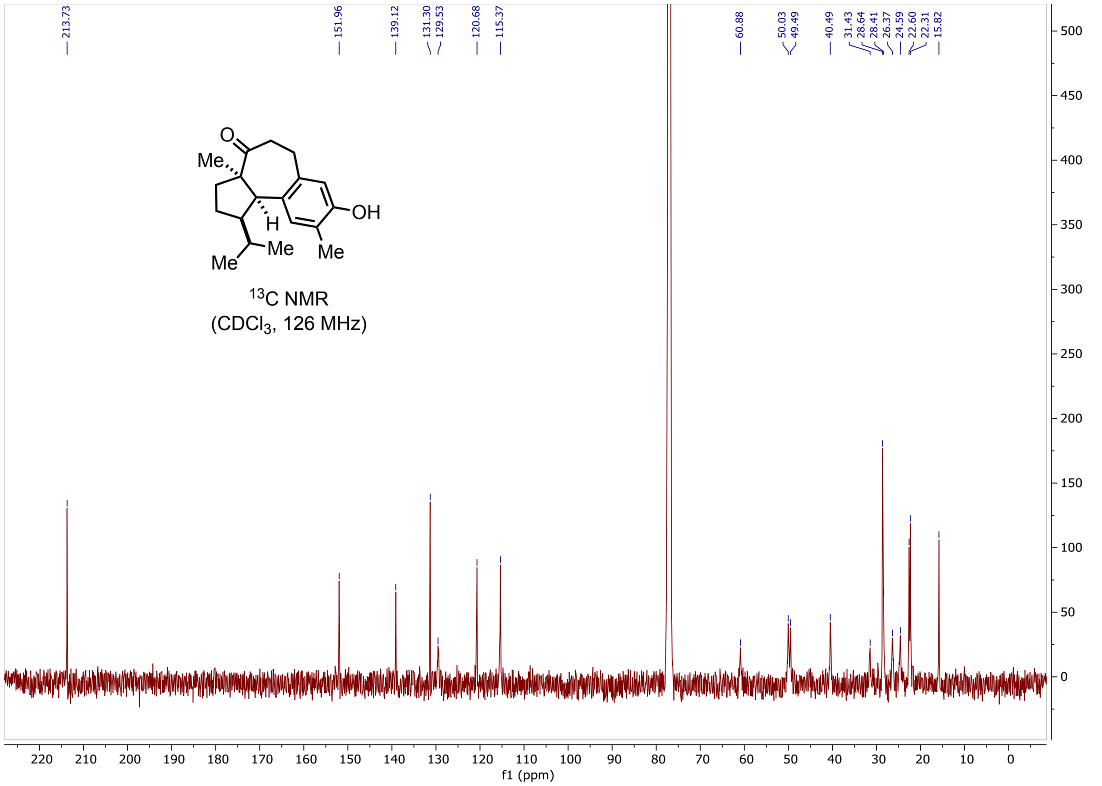

Me
O
OH
H
Me
Me
Me
^{13}C NMR
(CDCl$_3$, 126 MHz)
f1 (ppm)

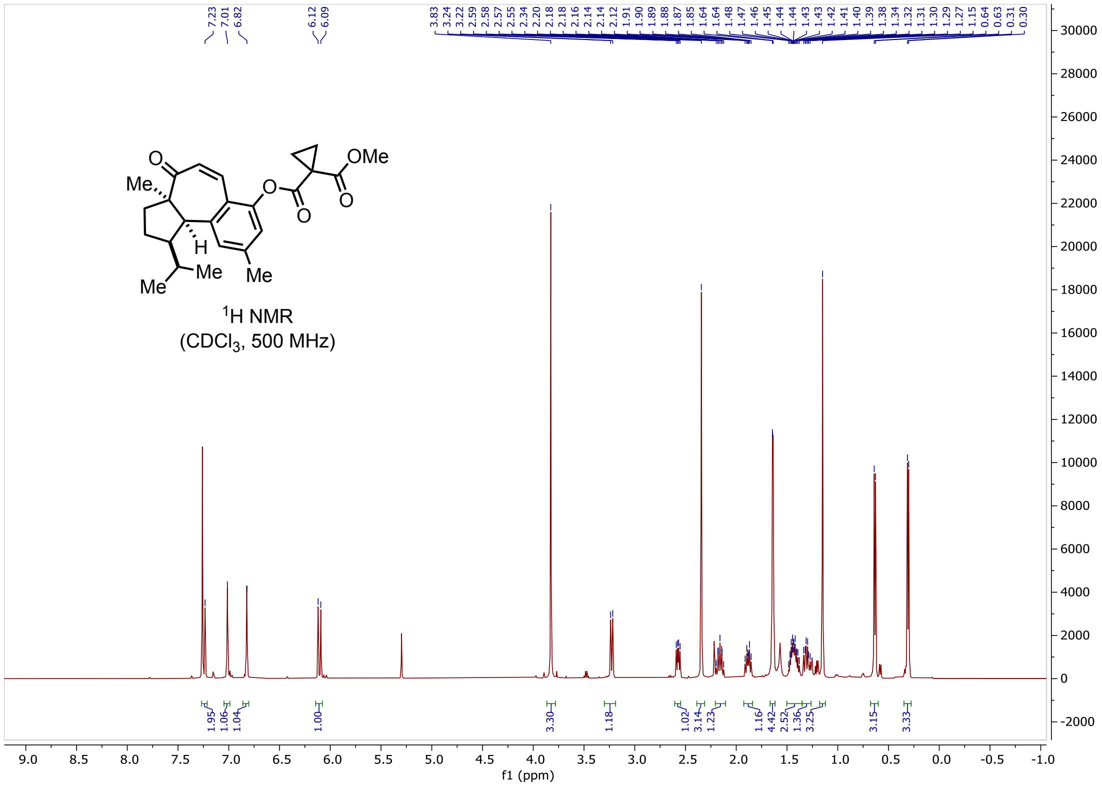

OMe
O
Me
O
O
O
H
Me
Me
Me
^{1}H NMR
(CDCl$_3$, 500 MHz)
f1 (ppm)

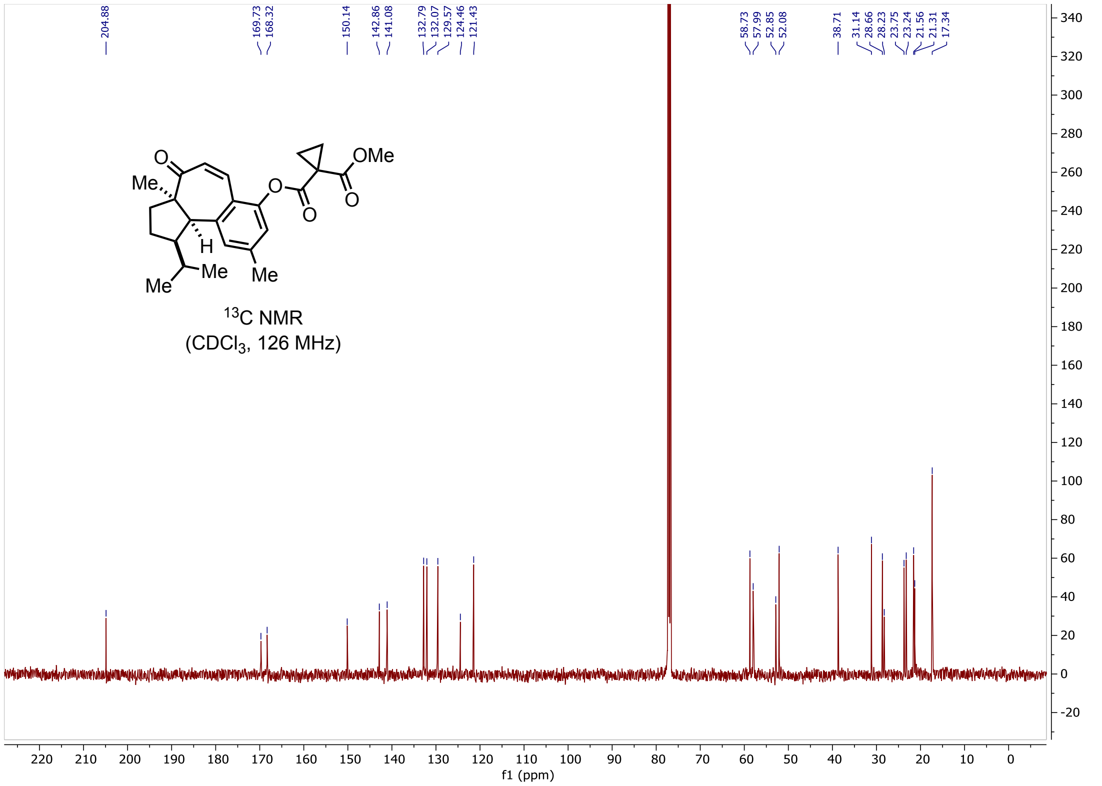

204.88
169.73
168.32
150.14
142.86
141.08
132.79
132.07
129.57
124.46
121.43
58.73
57.99
52.85
52.08
38.71
31.14
28.66
28.23
23.75
23.24
21.56
21.31
17.34
OMe
Me
H
Me
Me
Me
^{13}C NMR
(CDCl$_3$, 126 MHz)
f1 (ppm)

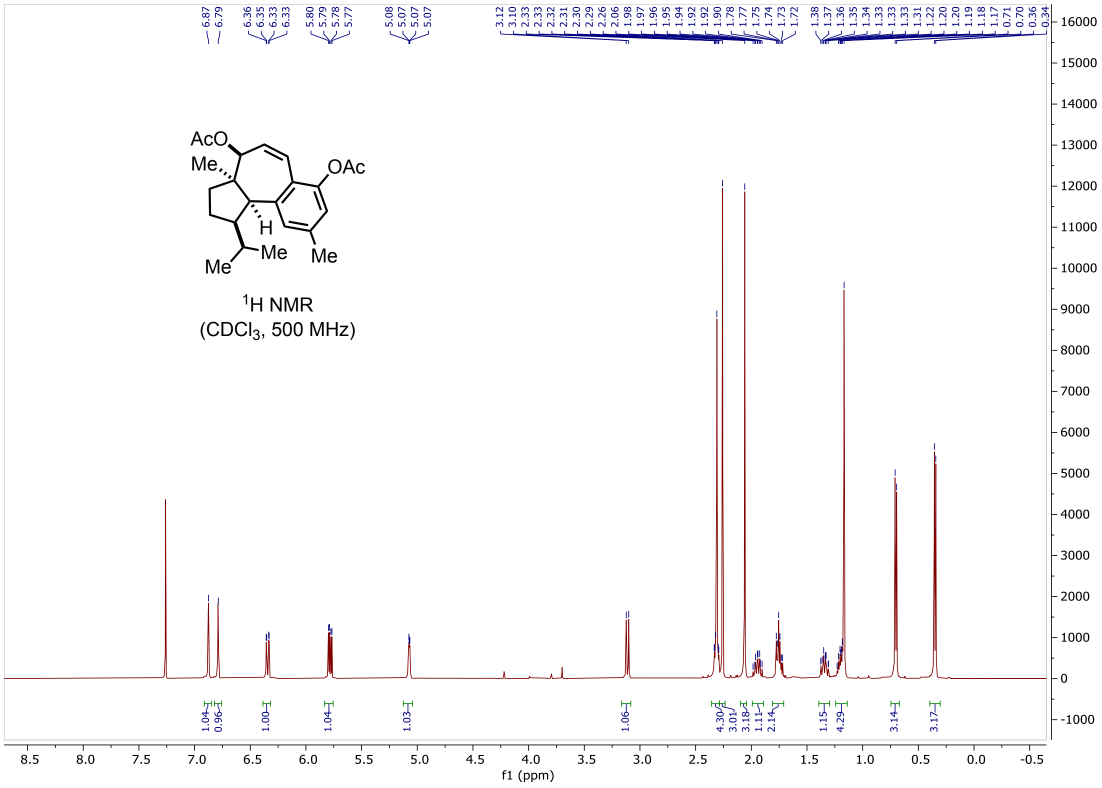
AcO
Me
OAc
H
Me
Me
Me
^{1}H NMR
(CDCl$_3$, 500 MHz)
f1 (ppm)

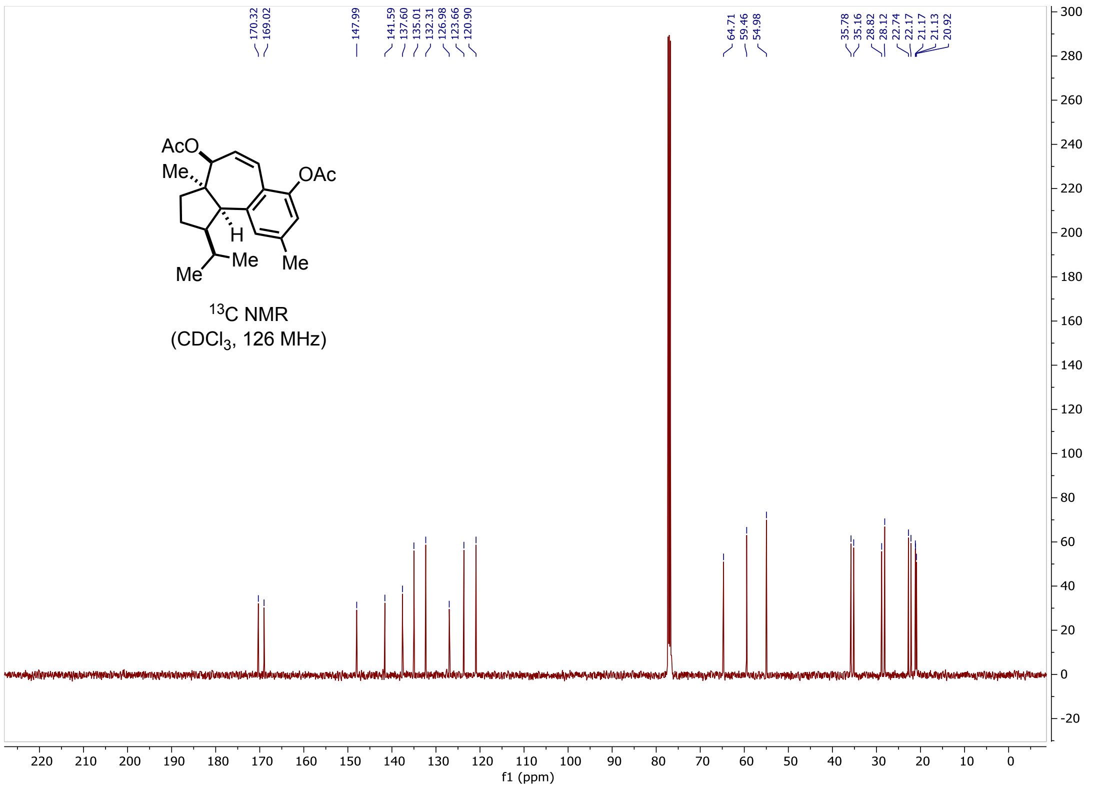
AcO
Me
OAc
H
Me
Me
Me
^{13}C NMR
(CDCl$_3$, 126 MHz)
f1 (ppm)

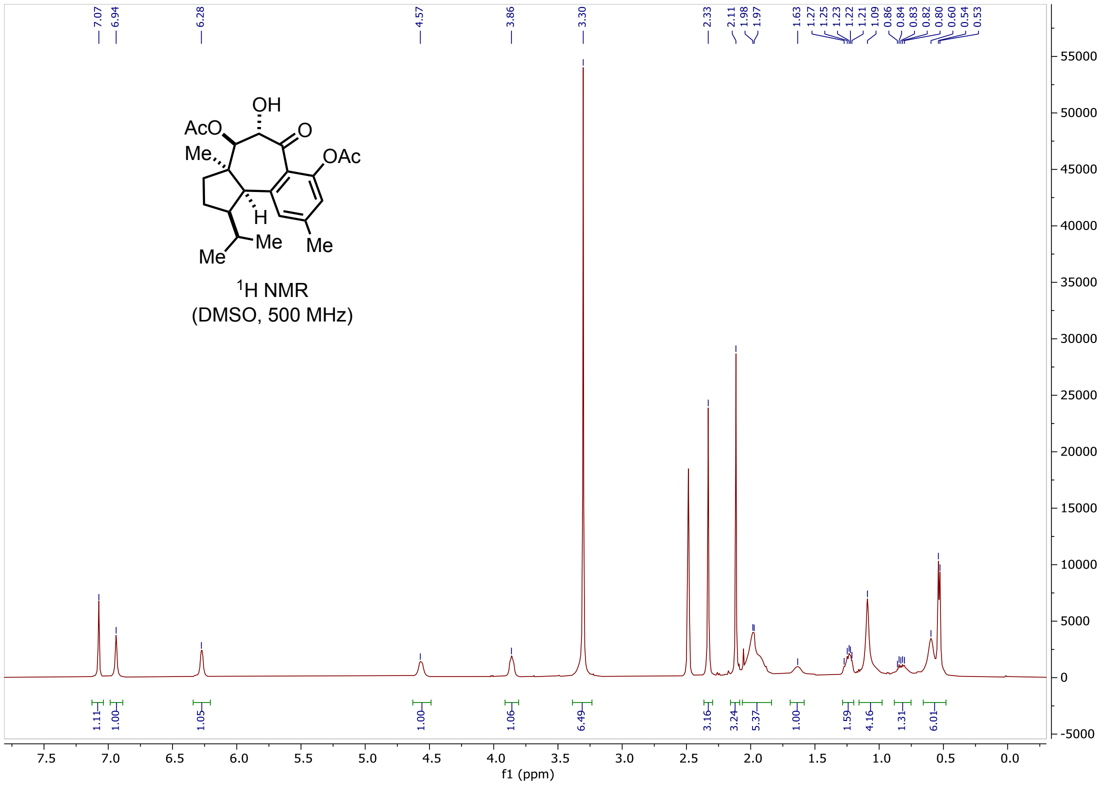

OH
AcO
O
Me
OAc
H
Me
Me
Me
^{1}H NMR
(DMSO, 500 MHz)
7.07
6.94
6.28
4.57
3.86
3.30
2.33
2.11
1.98
1.97
1.63
1.27
1.25
1.23
1.22
1.21
1.09
0.86
0.84
0.83
0.82
0.80
0.60
0.54
0.53
1.11
1.00
1.05
1.00
1.06
6.49
3.16
3.24
5.37
1.00
1.59
4.16
1.31
6.01
f1 (ppm)

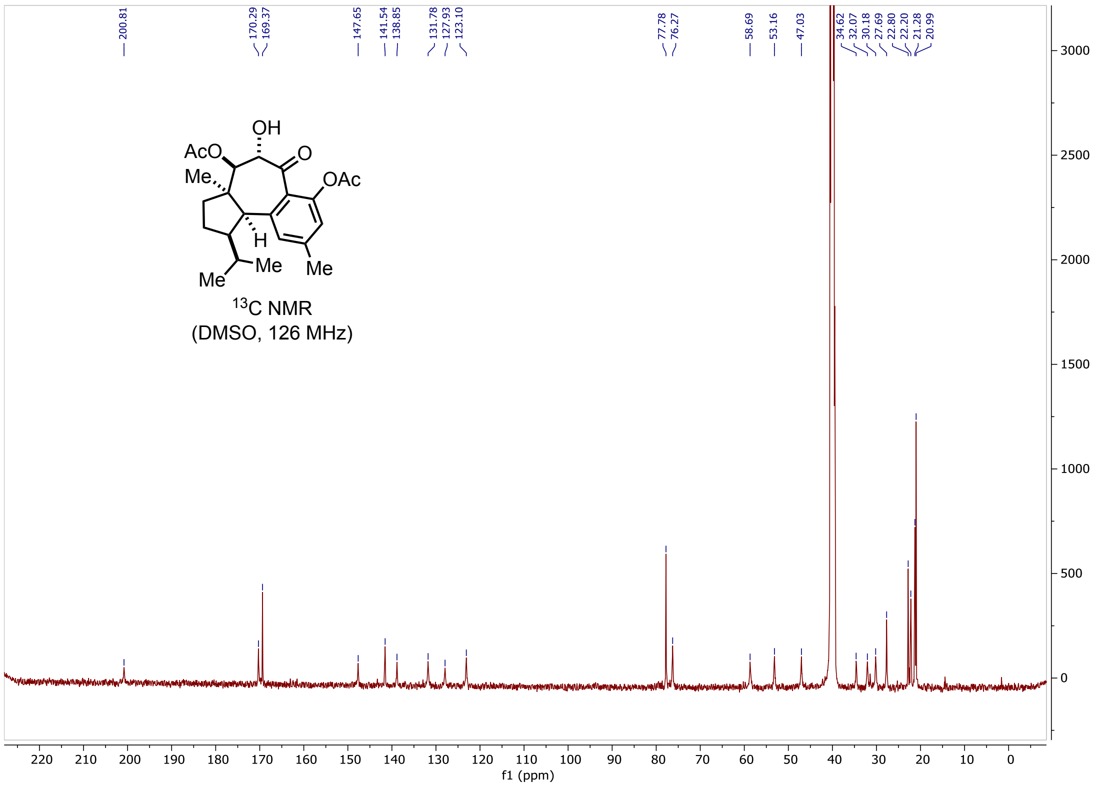

OH
AcO
O
Me
OAc
H
Me
Me
Me
^{13}C NMR
(DMSO, 126 MHz)
200.81
170.29
169.37
147.65
141.54
138.85
131.78
127.93
123.10
77.78
76.27
58.69
53.16
47.03
34.62
32.07
30.18
27.69
22.80
22.20
21.28
20.99
f1 (ppm)

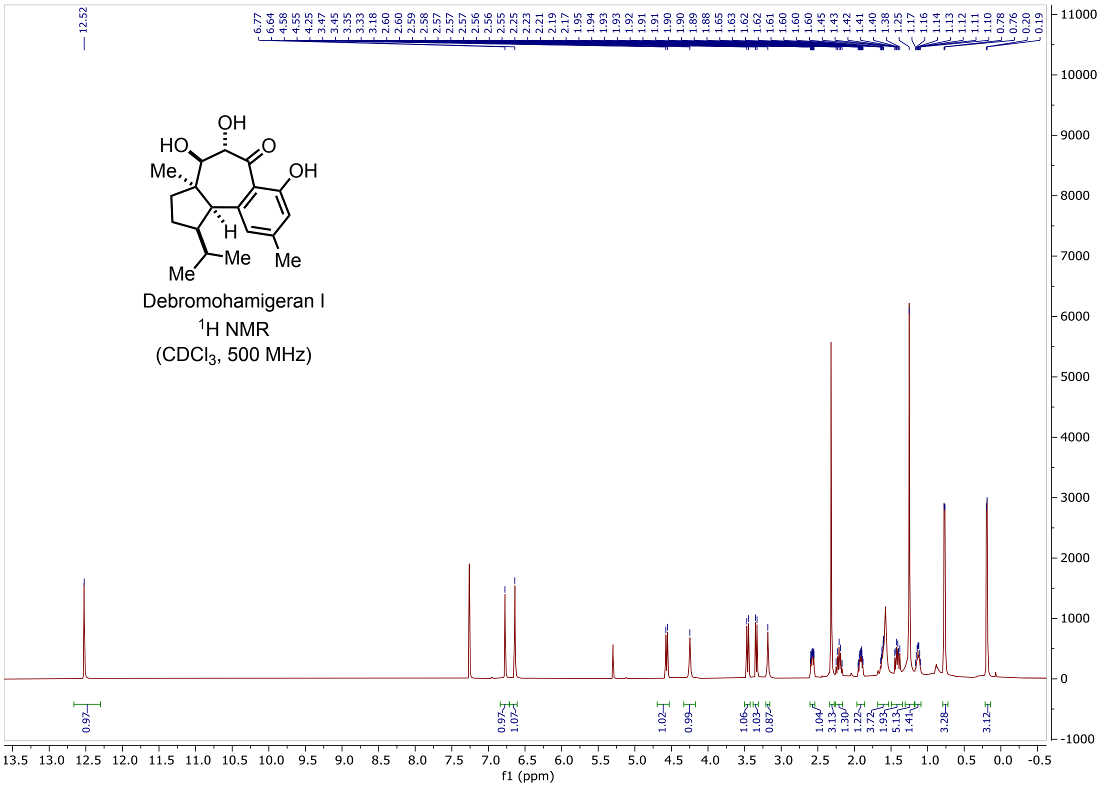

Debromohamigeran I
^{1}H NMR
($CDCl_3$, 500 MHz)
f1 (ppm)

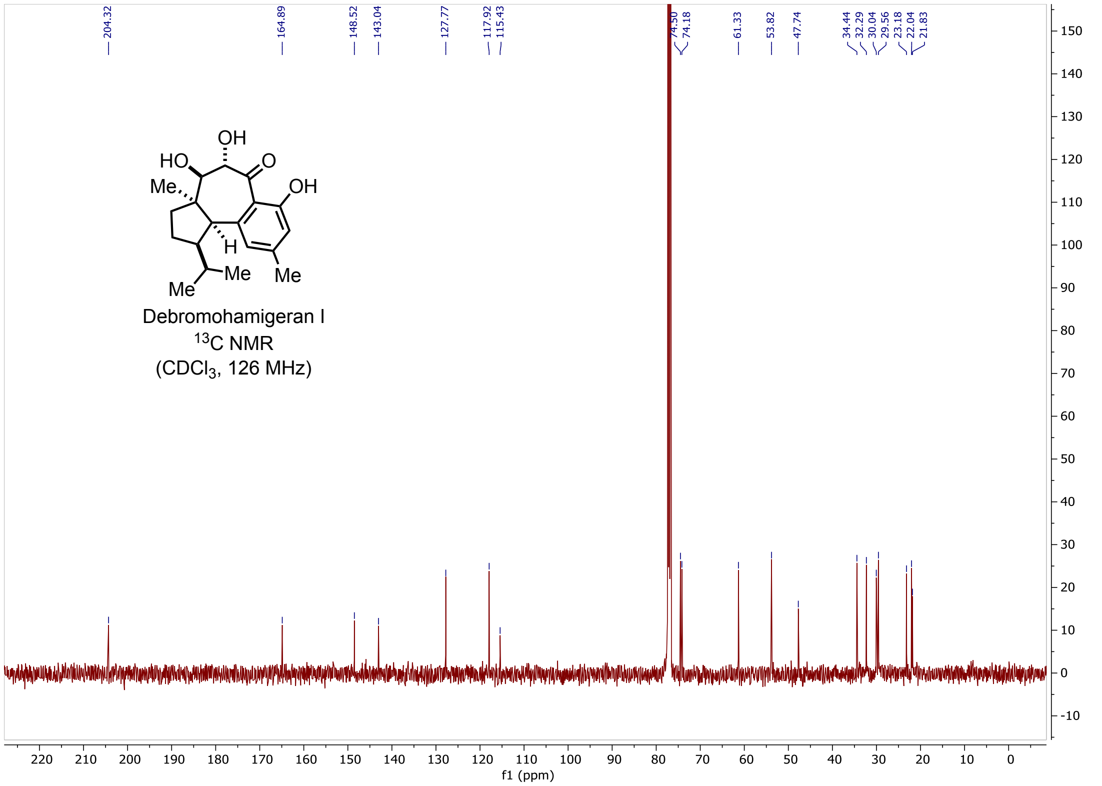

Debromohamigeran I
^{13}C NMR
($CDCl_3$, 126 MHz)
f1 (ppm)

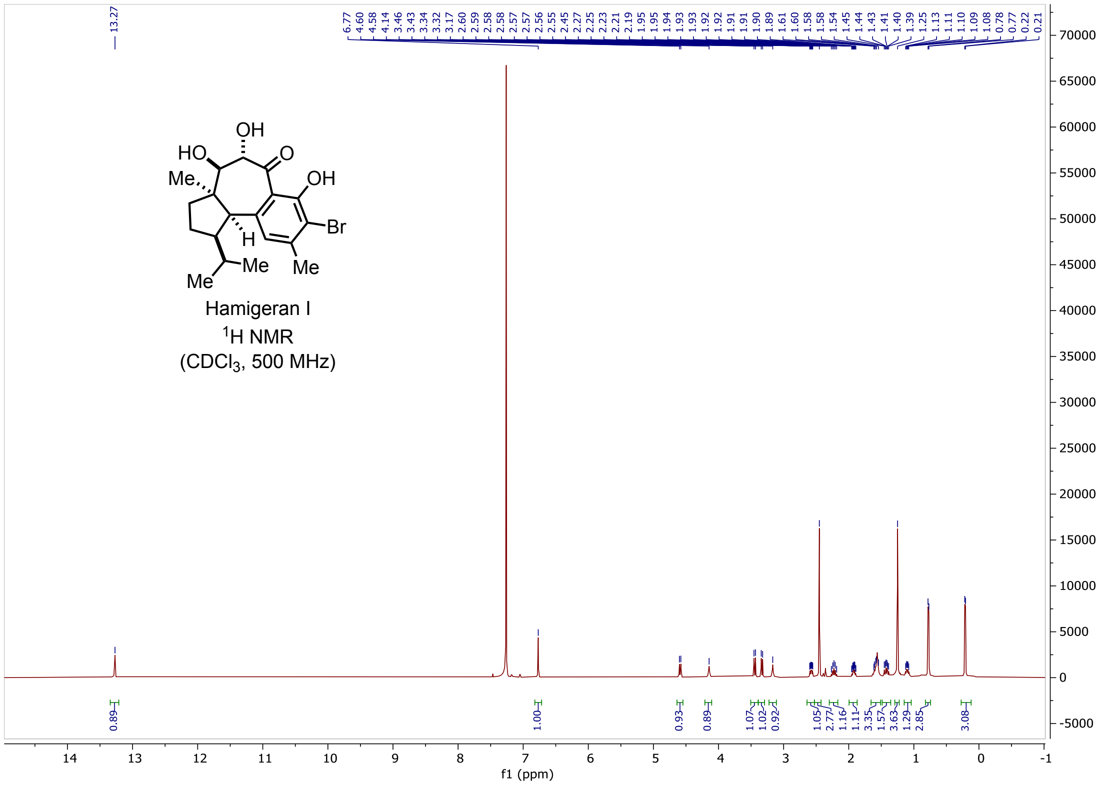
Hamigeran I
^{1}H NMR
($CDCl_3$, 500 MHz)

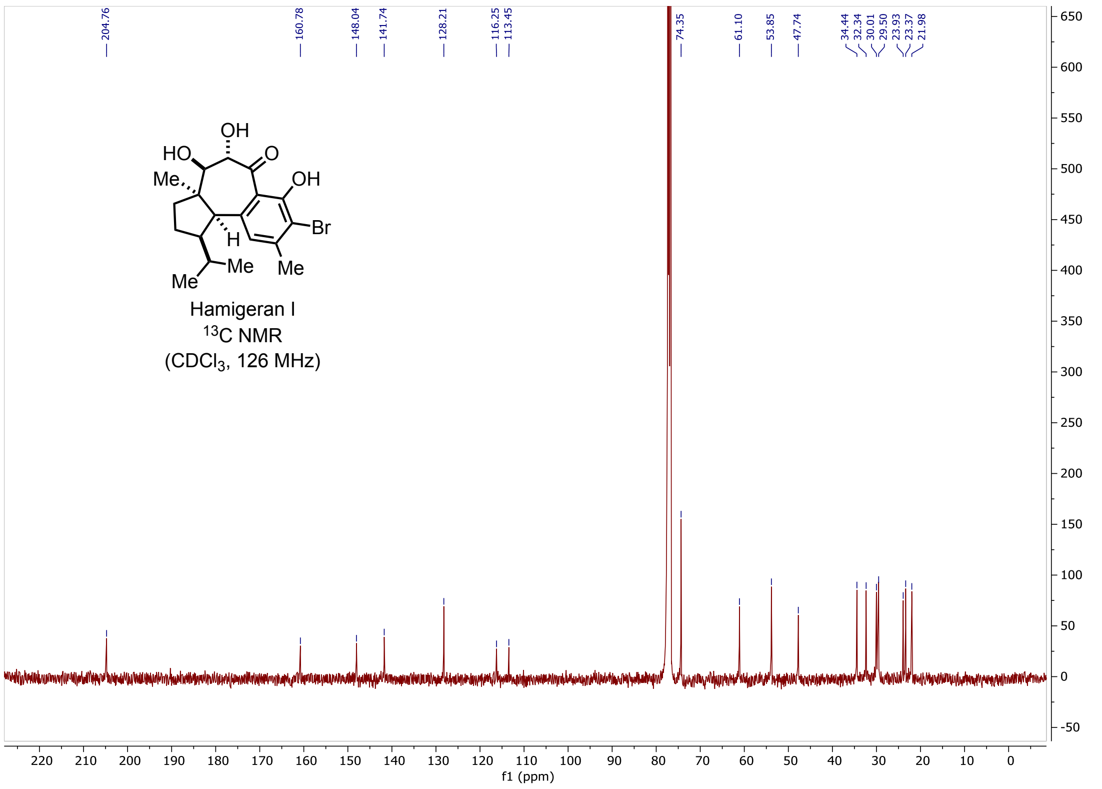
Hamigeran I
^{13}C NMR
($CDCl_3$, 126 MHz)

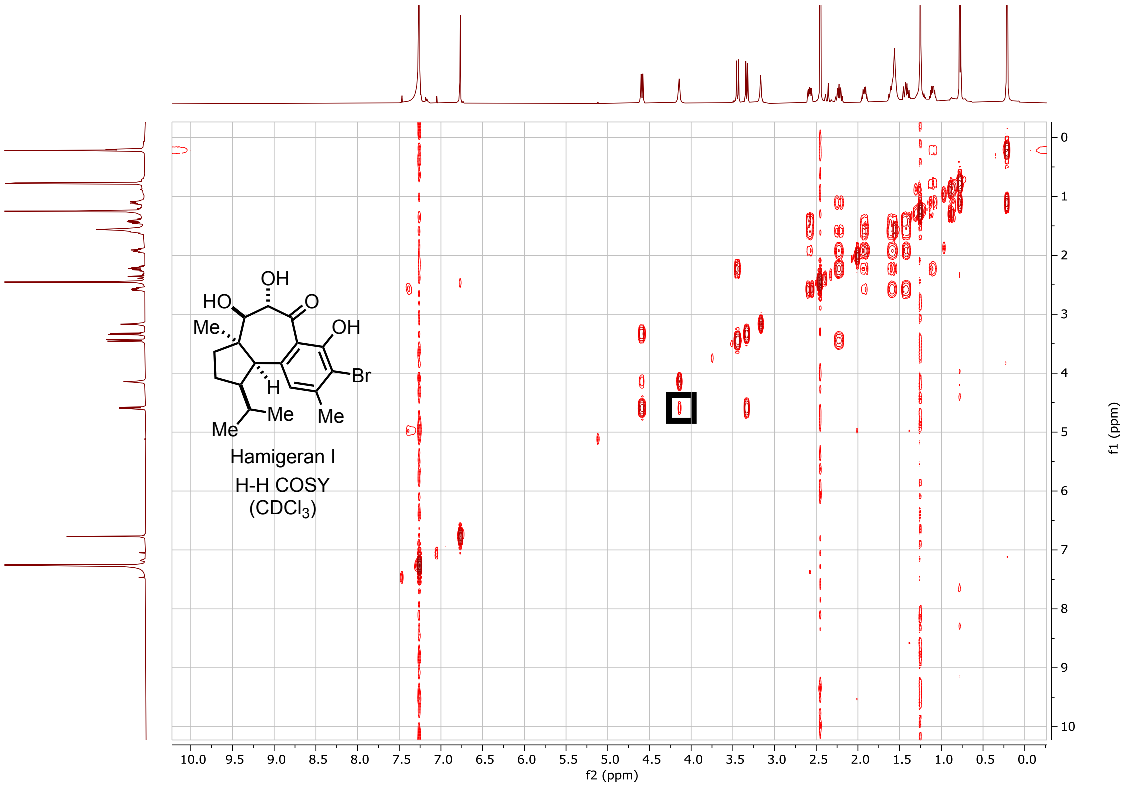
Hamigeran I
H-H COSY
(CDCl3)
f2 (ppm)
f1 (ppm)

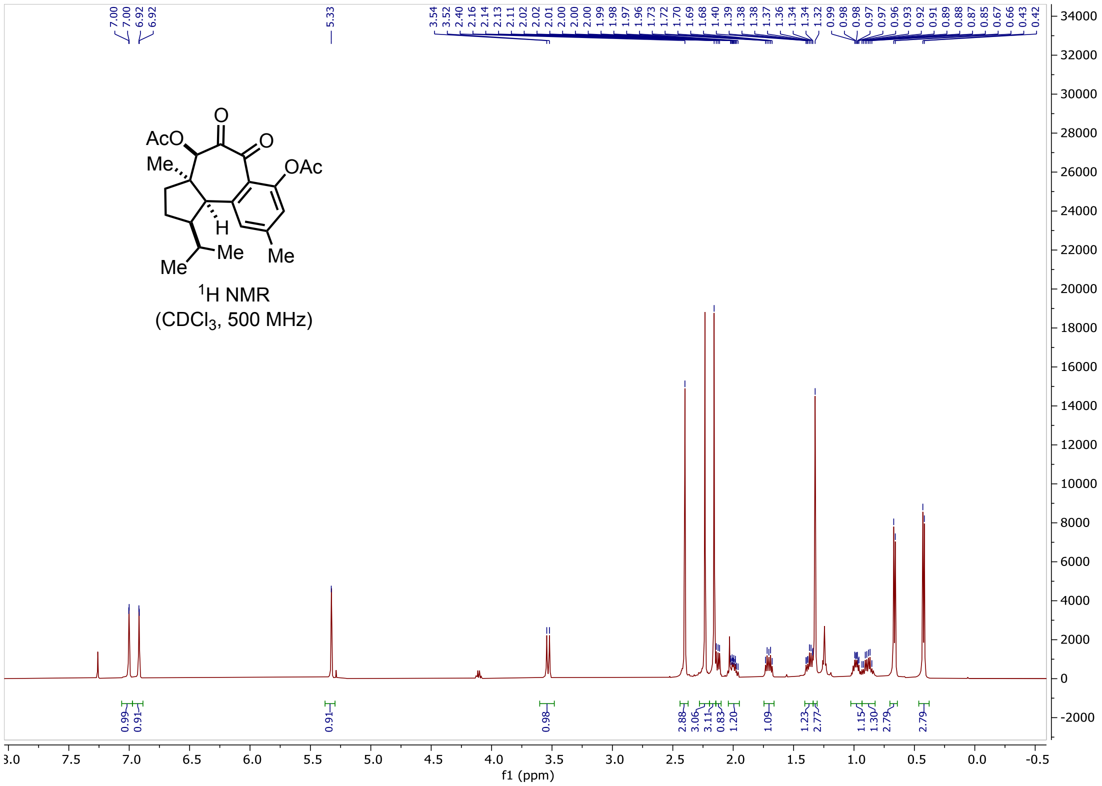

AcO
Me
OAc
H
Me
Me
Me
^{1}H NMR
($CDCl_3$, 500 MHz)
f1 (ppm)

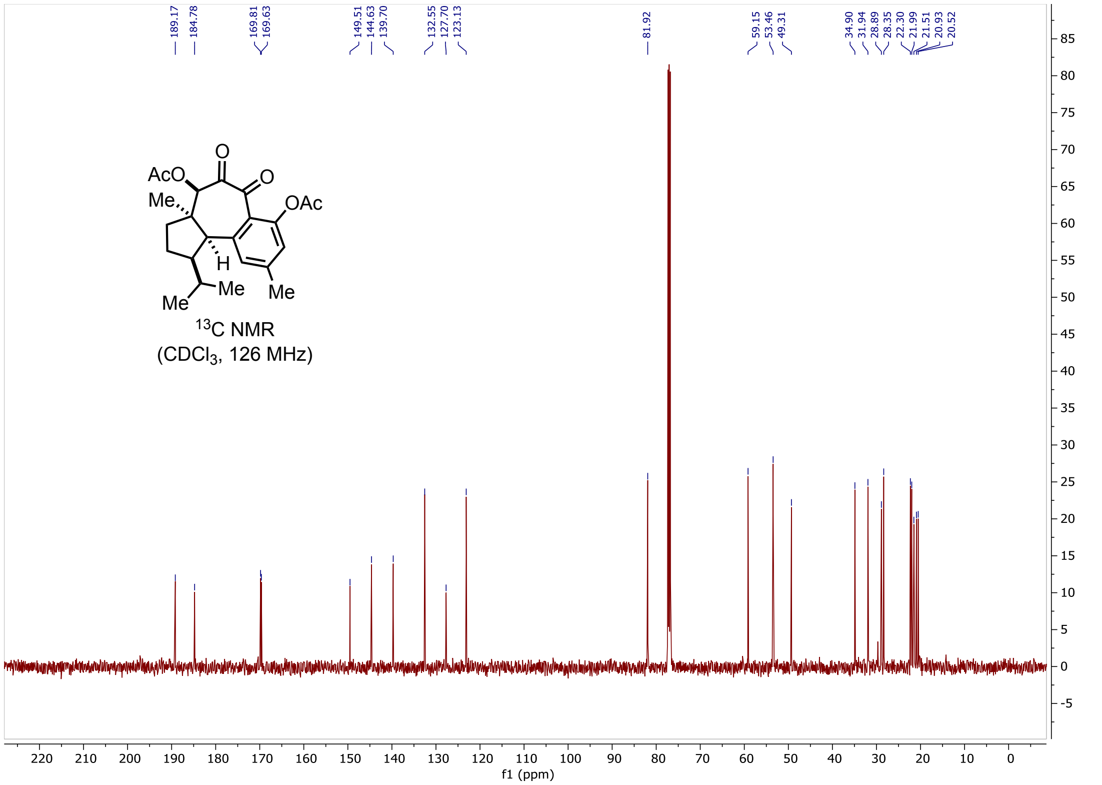

AcO
Me
OAc
H
Me
Me
Me
^{13}C NMR
($CDCl_3$, 126 MHz)
f1 (ppm)

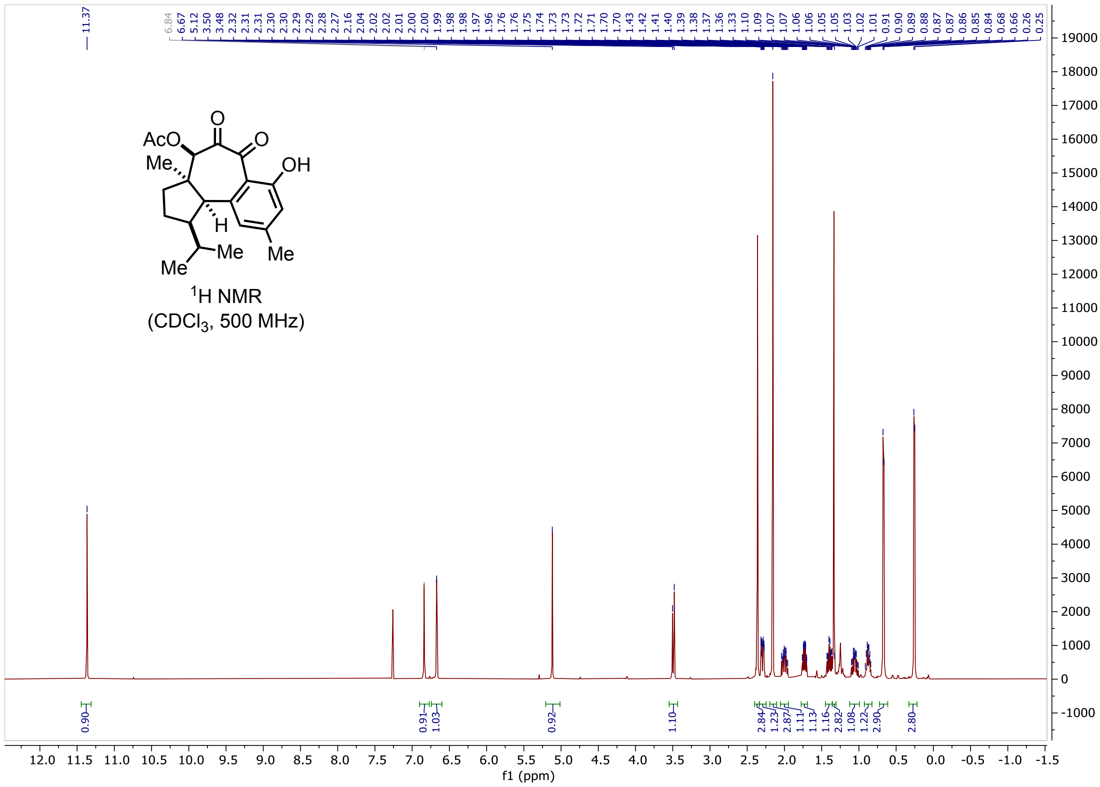
AcO
O
O
OH
Me
H
Me
Me
Me
^{1}H NMR
($CDCl_3$, 500 MHz)
11.37
6.84
6.67
5.12
3.50
3.48
0.90
0.91
1.03
0.92
1.10
2.84
1.23
2.87
1.11
1.13
1.16
2.82
1.08
1.22
2.90
2.80
f1 (ppm)

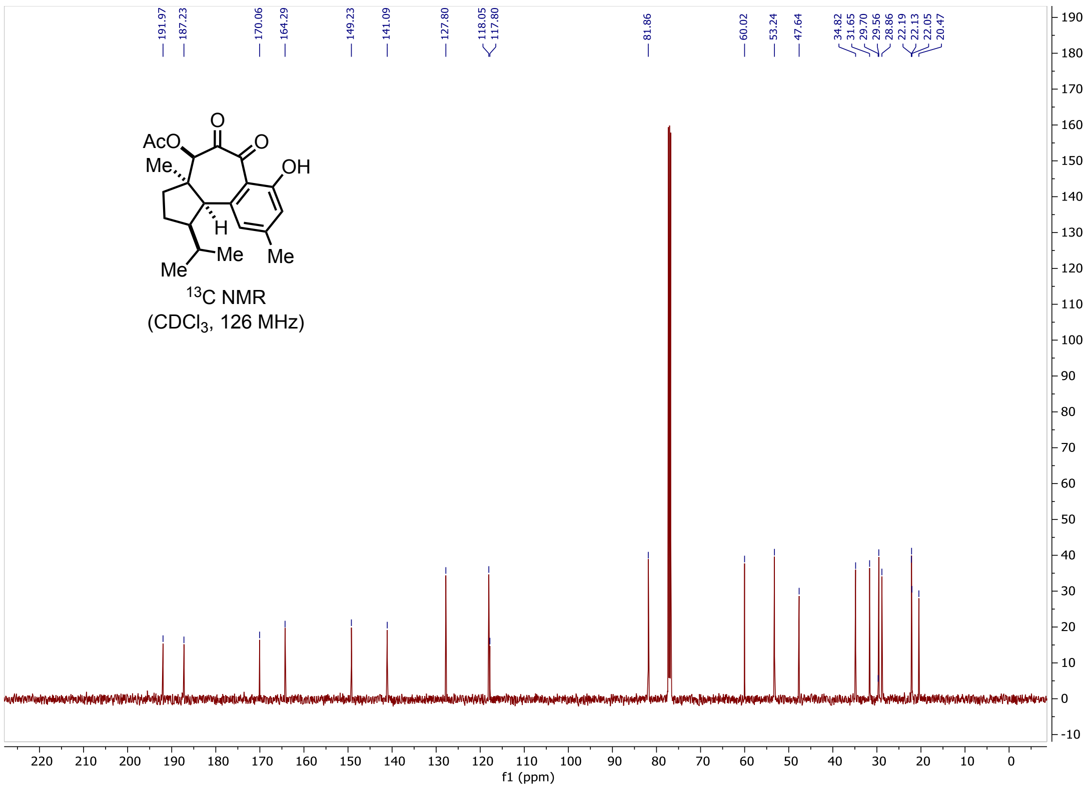
AcO
O
O
OH
Me
H
Me
Me
Me
^{13}C NMR
($CDCl_3$, 126 MHz)
191.97
187.23
170.06
164.29
149.23
141.09
127.80
118.05
117.80
81.86
60.02
53.24
47.64
34.82
31.65
29.70
29.56
28.86
22.19
22.13
22.05
20.47
f1 (ppm)

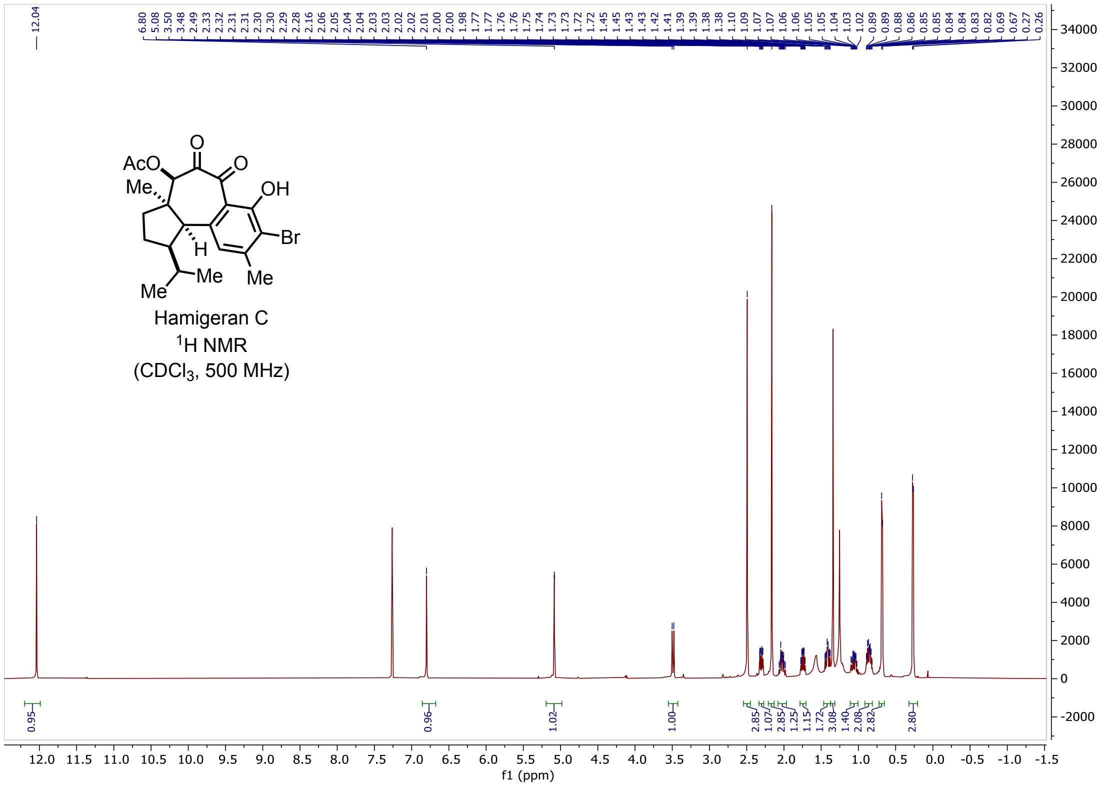
AcO
Me
OH
Br
H
Me
Me
Me
Hamigeran C
1H NMR
(CDCl3, 500 MHz)
f1 (ppm)

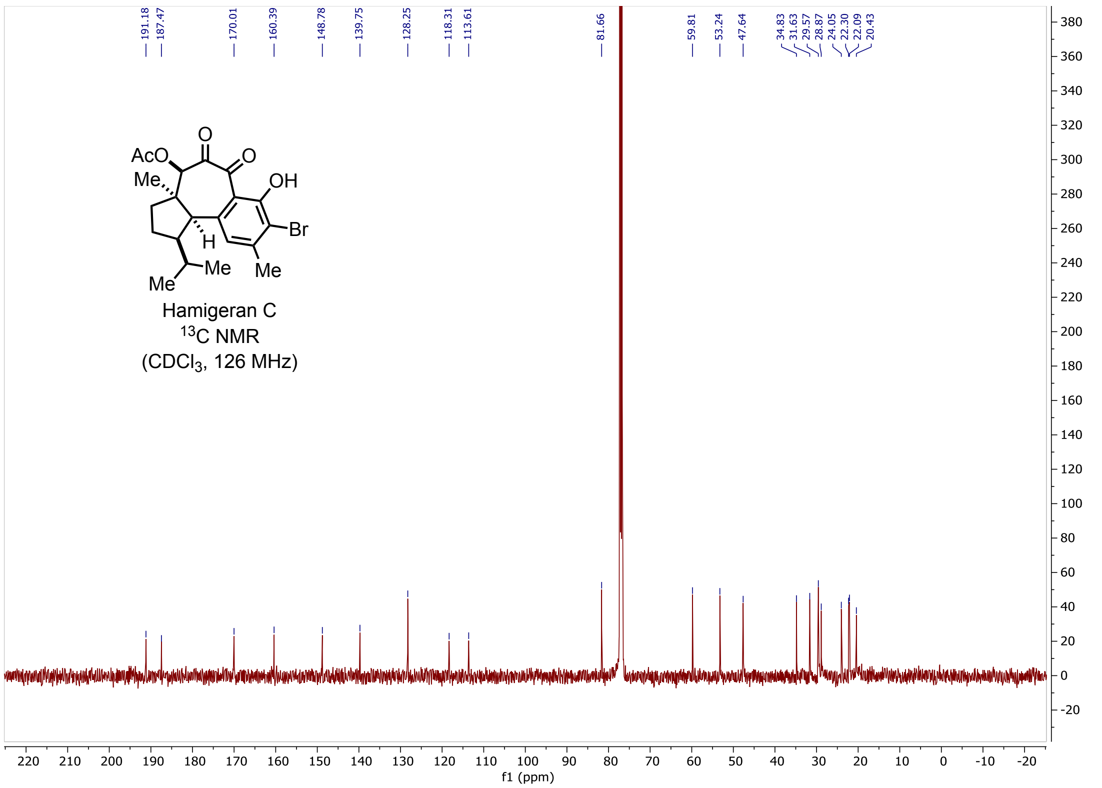
191.18
187.47
170.01
160.39
148.78
139.75
128.25
118.31
113.61
81.66
59.81
53.24
47.64
34.83
31.63
29.57
28.87
24.05
22.30
22.09
20.43
AcO
Me
OH
Br
H
Me
Me
Me
Hamigeran C
13C NMR
(CDCl3, 126 MHz)
f1 (ppm)

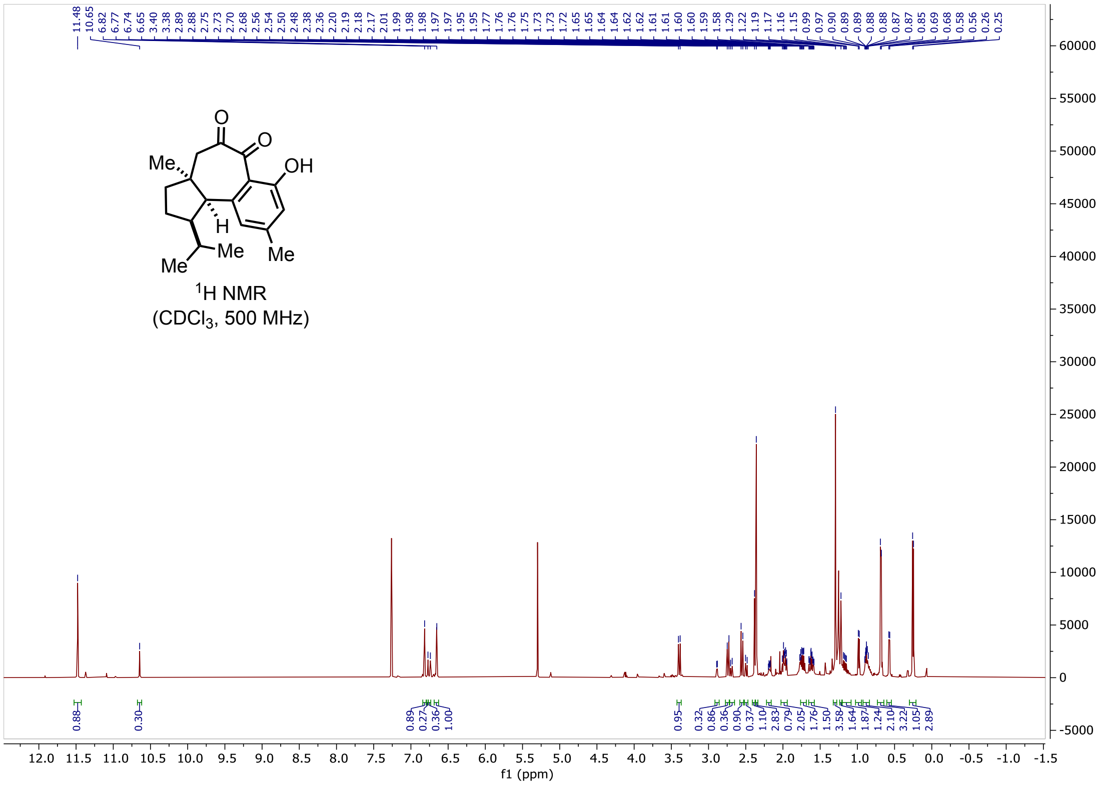

Me
O
O
OH
H
Me
Me
Me
^{1}H NMR
($CDCl_3$, 500 MHz)
f1 (ppm)

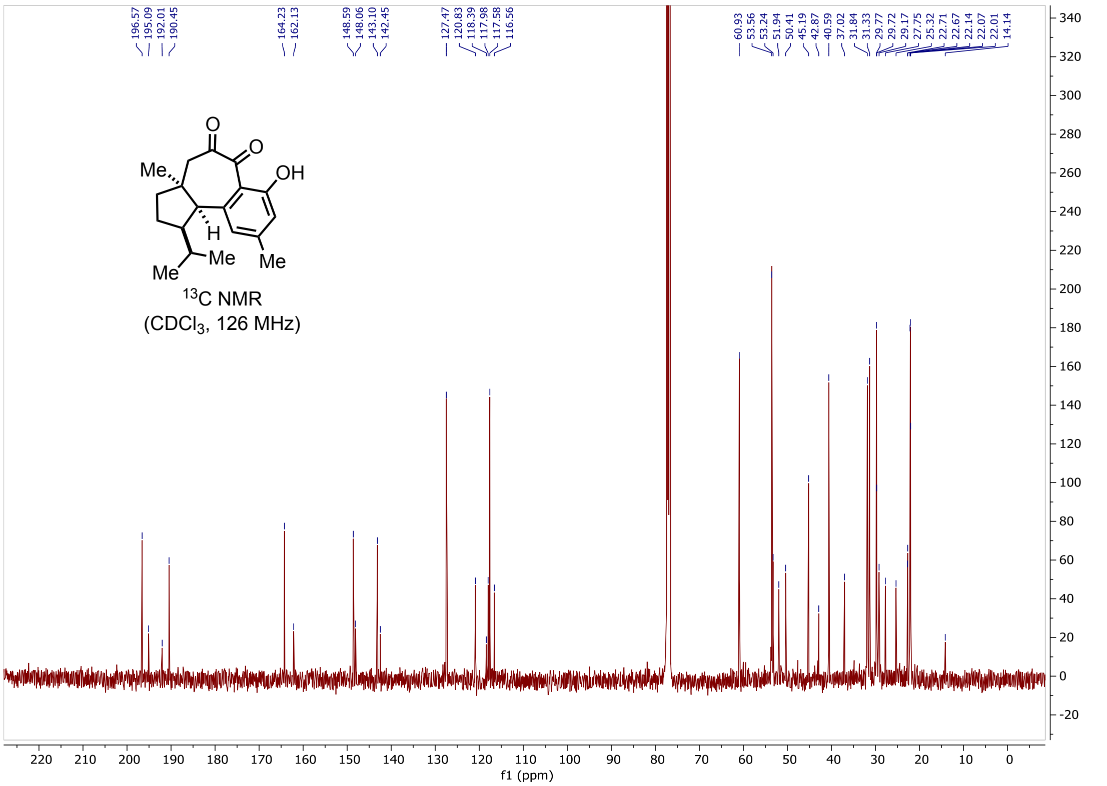

196.57
195.09
192.01
190.45
164.23
162.13
148.59
148.06
143.10
142.45
127.47
120.83
118.39
117.98
117.58
116.56
60.93
53.56
53.24
51.94
50.41
45.19
42.87
40.59
37.02
31.84
31.33
29.77
29.72
29.17
27.75
25.32
22.71
22.67
22.14
22.07
22.01
14.14
Me
O
O
OH
H
Me
Me
Me
^{13}C NMR
($CDCl_3$, 126 MHz)
f1 (ppm)

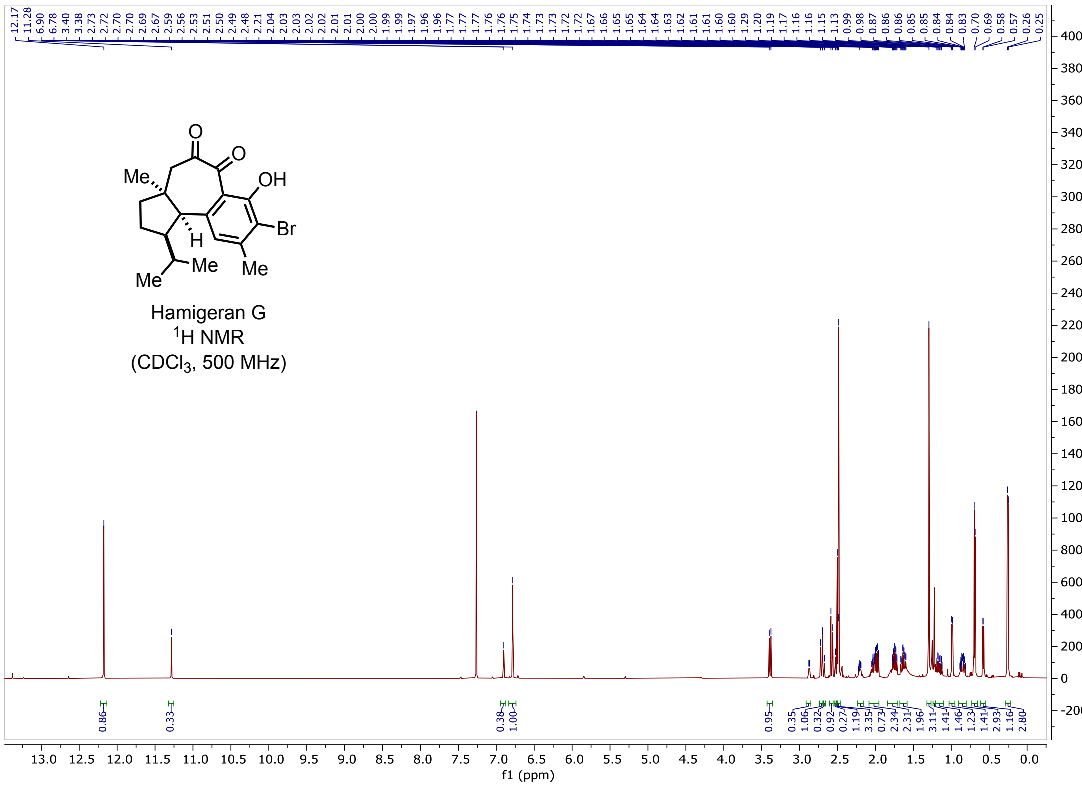
Hamigeran G
1H NMR
(CDCl3, 500 MHz)
f1 (ppm)

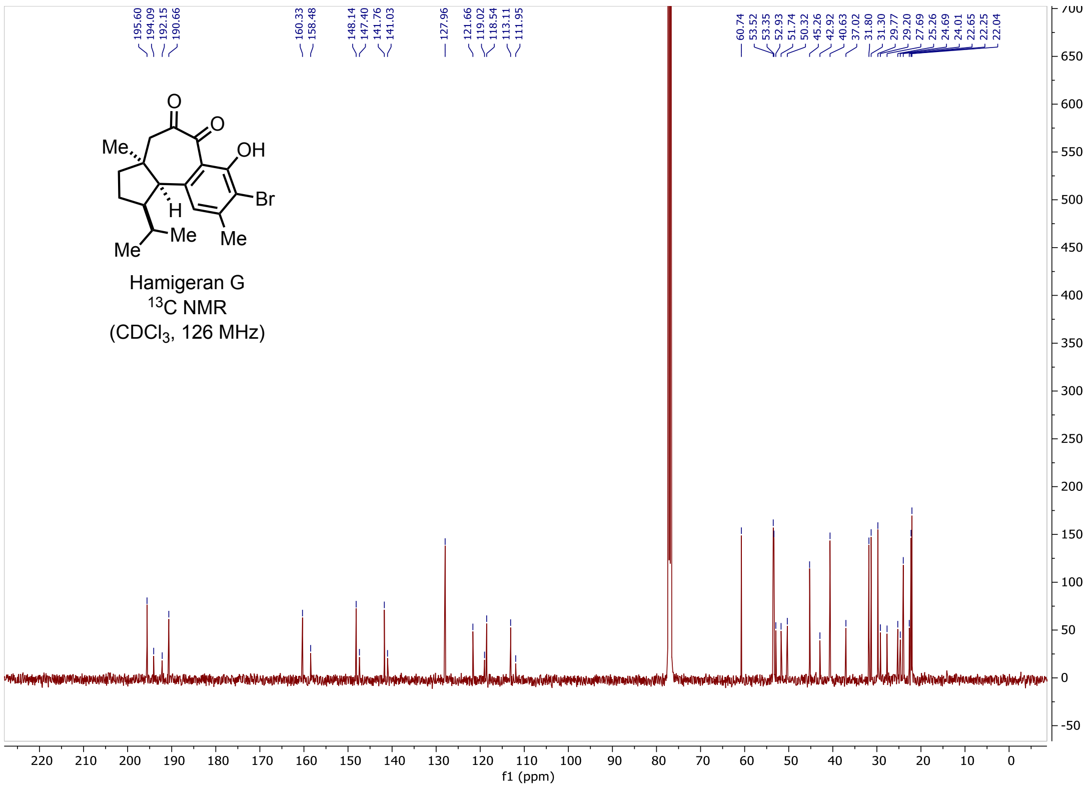
Hamigeran G
13C NMR
(CDCl3, 126 MHz)
f1 (ppm)

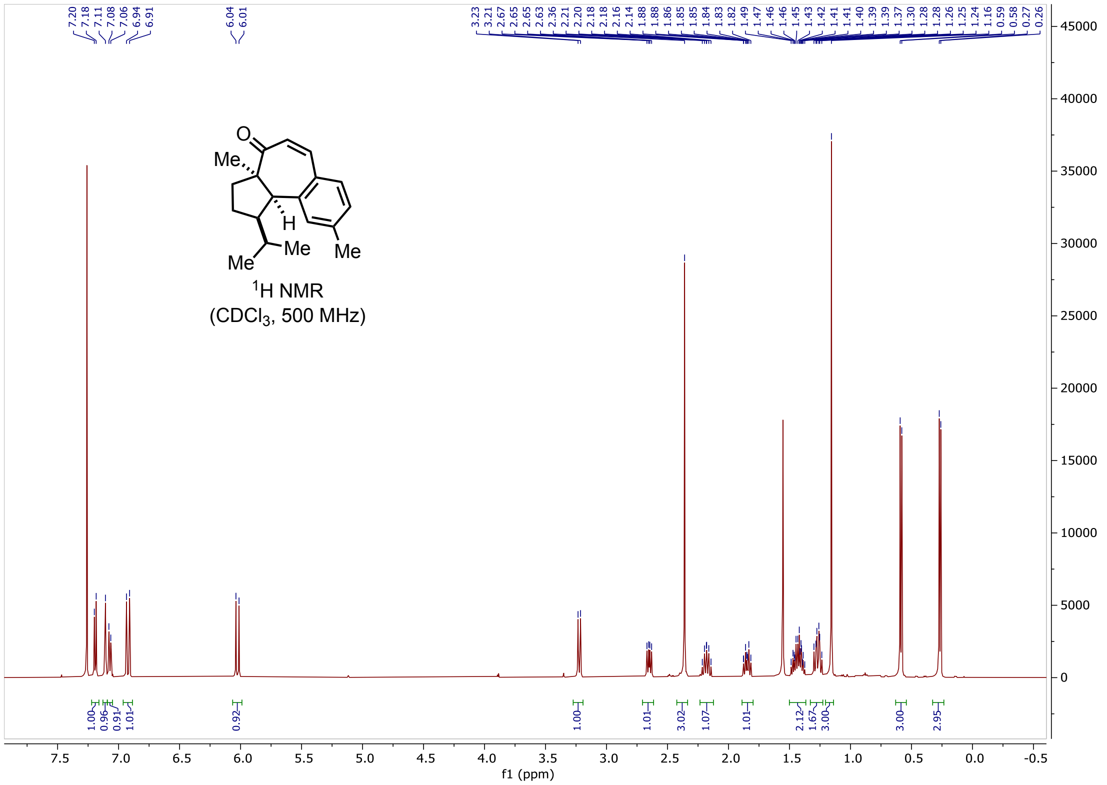
1H NMR
(CDCl3, 500 MHz)
f1 (ppm)

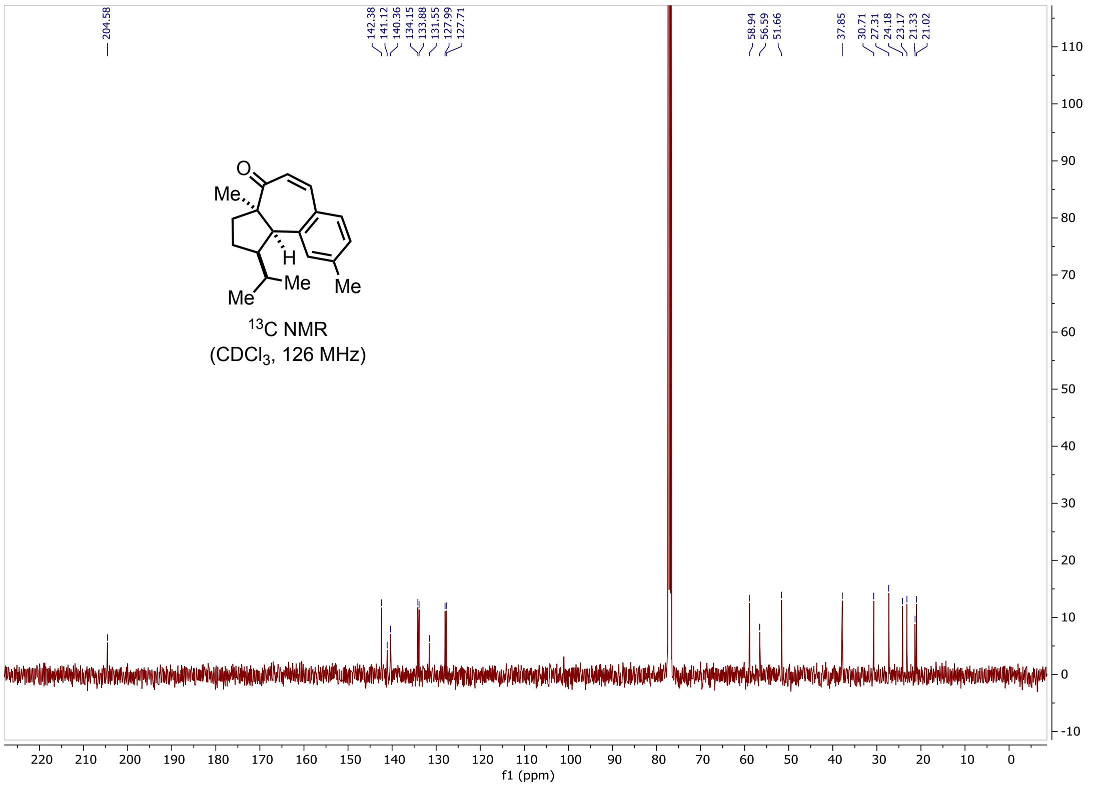
13C NMR
(CDCl3, 126 MHz)
f1 (ppm)

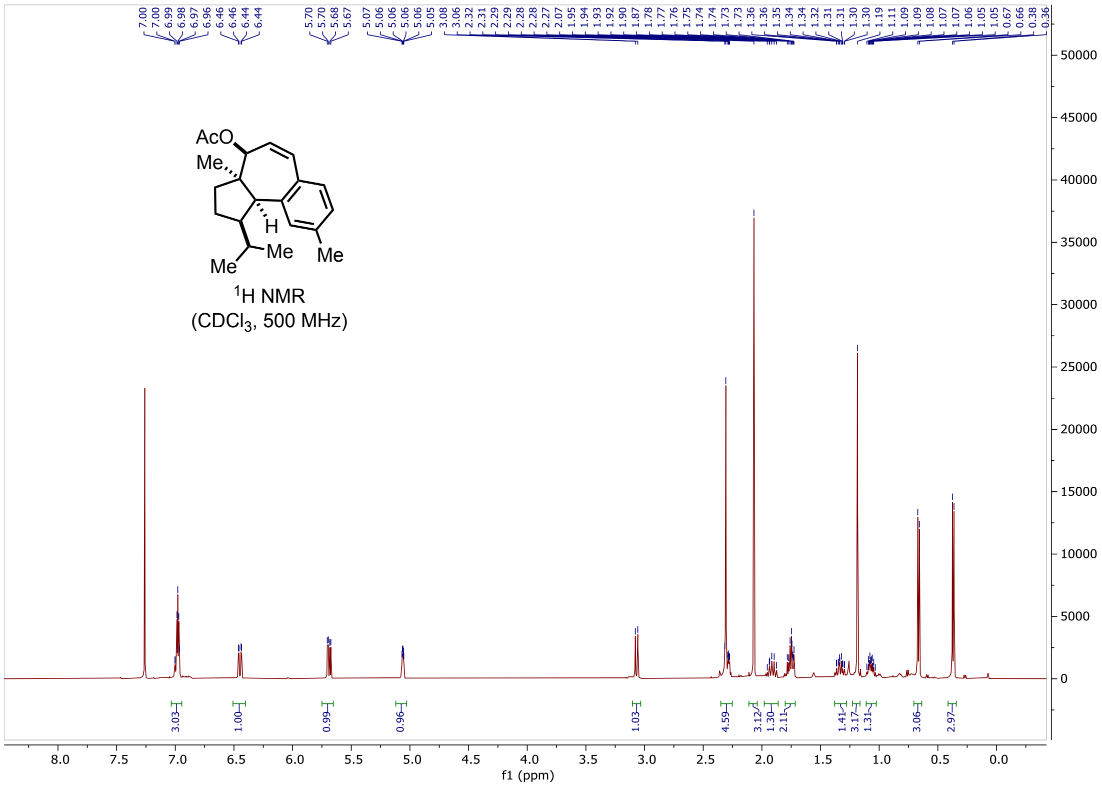

AcO
Me
H
Me
Me
Me
^{1}H NMR
(CDCl$_3$, 500 MHz)
f1 (ppm)

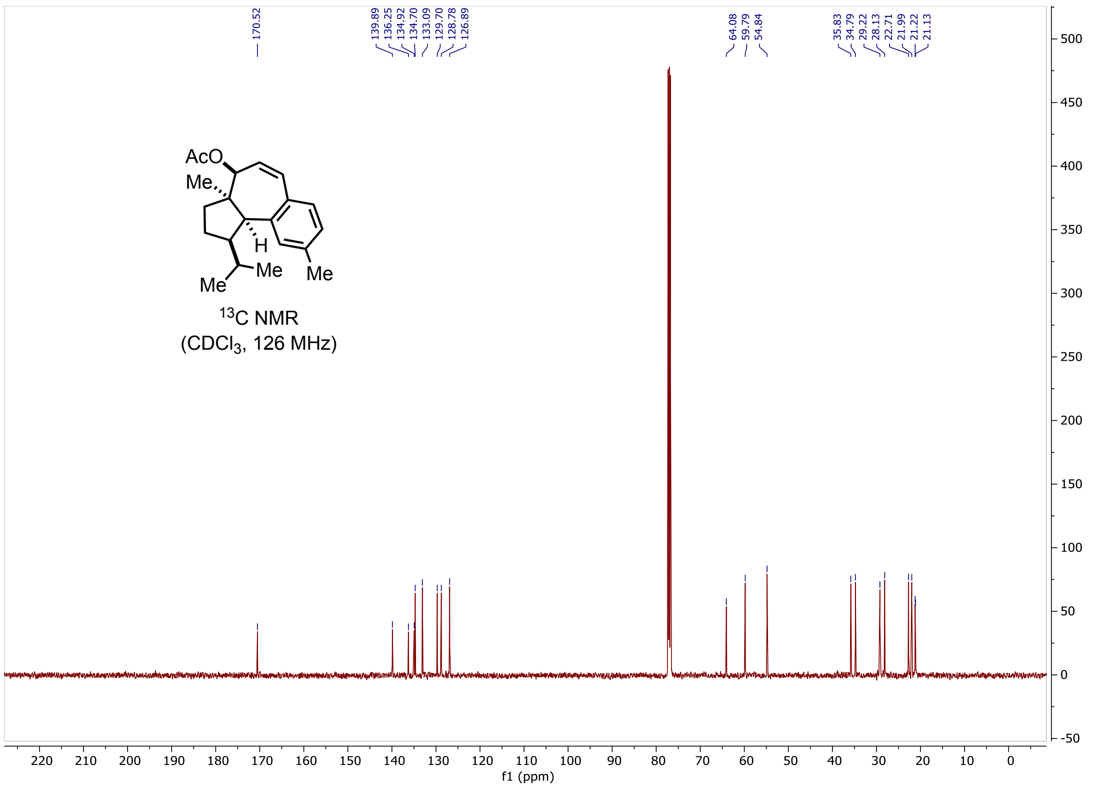

AcO
Me
H
Me
Me
Me
^{13}C NMR
(CDCl$_3$, 126 MHz)
f1 (ppm)

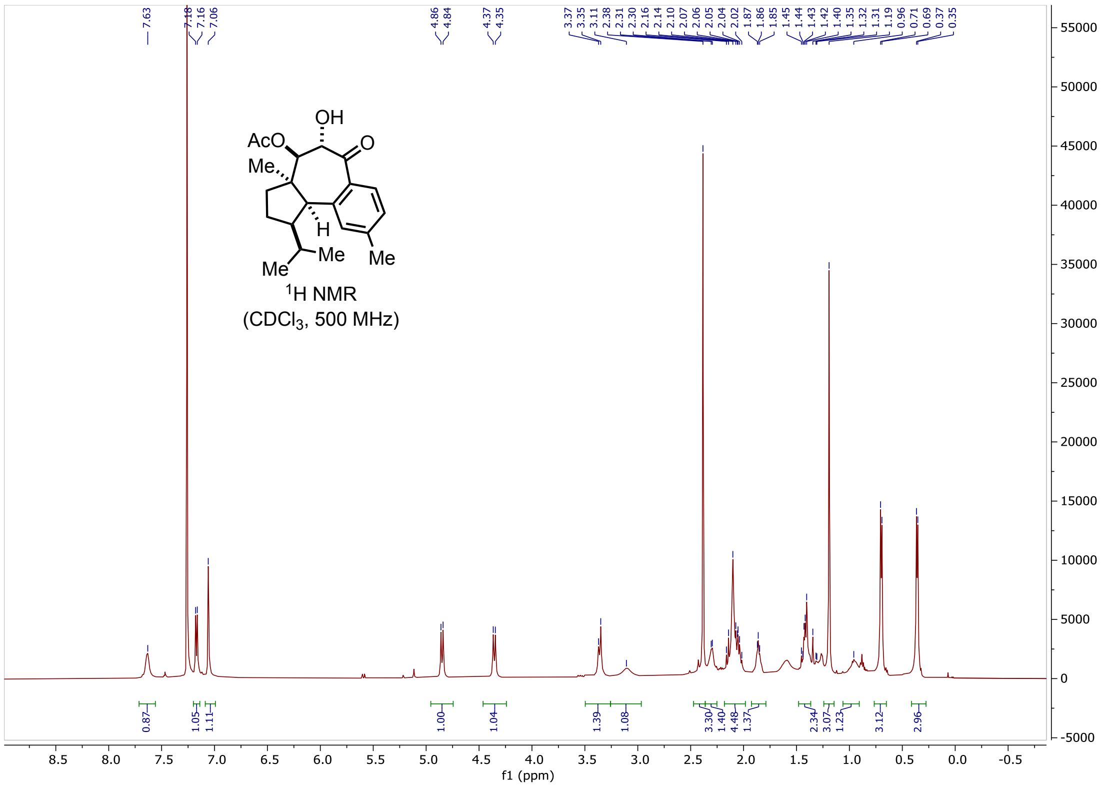
7.63
7.18
7.16
7.06
4.86
4.84
4.37
4.35
3.37
3.35
3.11
2.38
2.31
2.30
2.16
2.14
2.10
2.07
2.06
2.05
2.04
2.02
1.87
1.86
1.85
1.45
1.44
1.43
1.42
1.40
1.35
1.32
1.31
1.19
0.96
0.71
0.69
0.37
0.35
OH
AcO
O
Me
H
Me
Me
Me
1H NMR
(CDCl3, 500 MHz)
0.87
1.05
1.11
1.00
1.04
1.39
1.08
3.30
1.40
4.48
1.37
2.34
3.07
1.23
3.12
2.96
f1 (ppm)

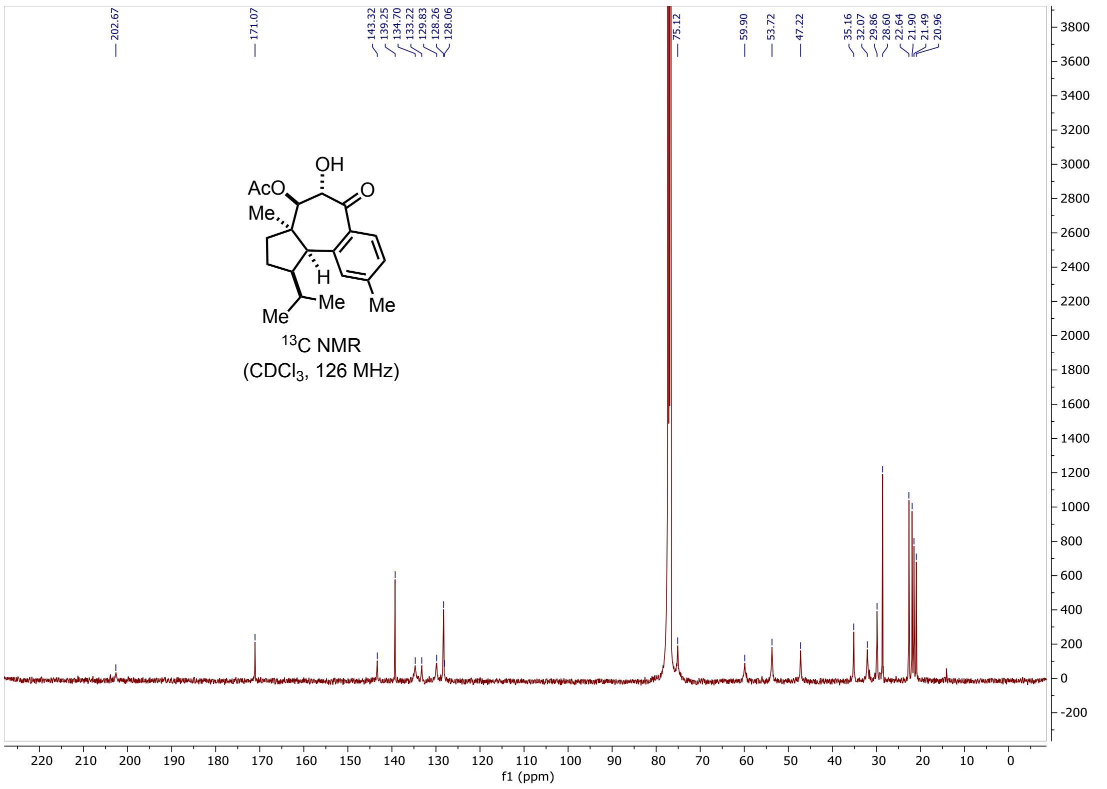
202.67
171.07
143.32
139.25
134.70
133.22
129.83
128.26
128.06
75.12
59.90
53.72
47.22
35.16
32.07
29.86
28.60
22.64
21.90
21.49
20.96
OH
AcO
O
Me
H
Me
Me
Me
13C NMR
(CDCl3, 126 MHz)
f1 (ppm)

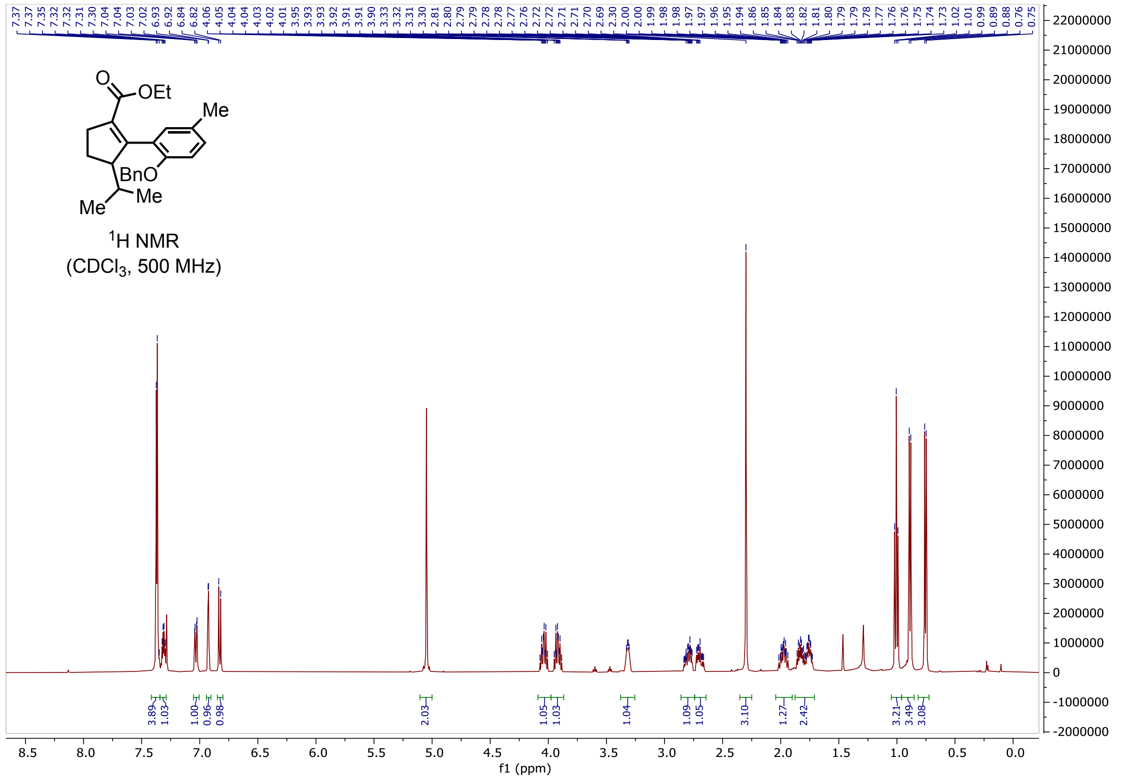
OEt
Me
BnO
Me
Me
^{1}H NMR
(CDCl$_3$, 500 MHz)
f1 (ppm)

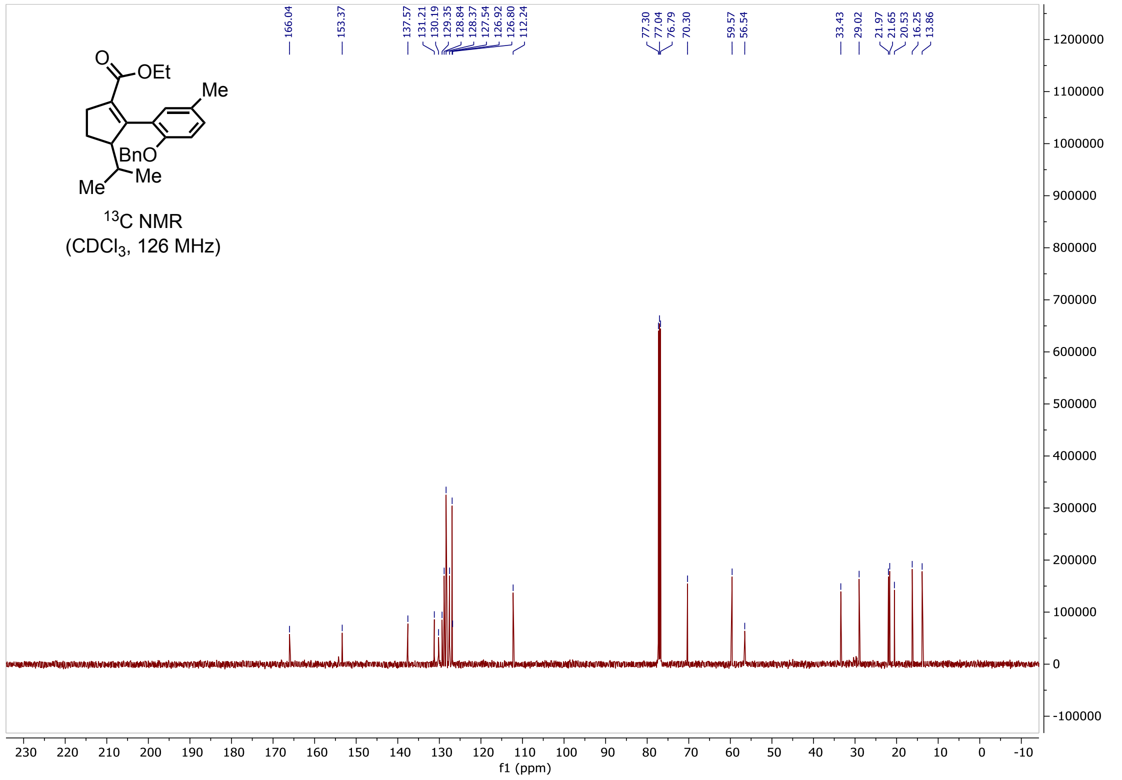
OEt
Me
BnO
Me
Me
^{13}C NMR
(CDCl$_3$, 126 MHz)
166.04
153.37
137.57
131.21
130.19
129.35
128.84
128.37
127.54
126.92
126.80
112.24
77.30
77.04
76.79
70.30
59.57
56.54
33.43
29.02
21.97
21.65
20.53
16.25
13.86
f1 (ppm)

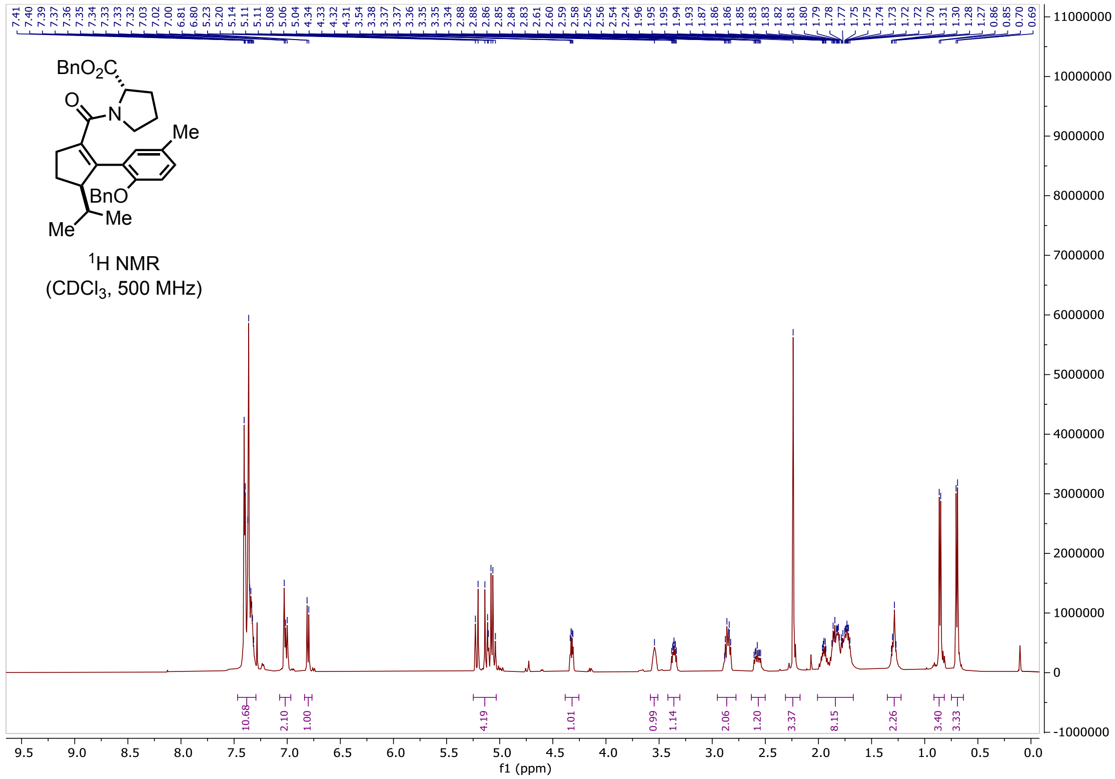
BnO$_2$C
O
N
Me
BnO
Me
Me
^{1}H NMR
(CDCl$_3$, 500 MHz)
f1 (ppm)

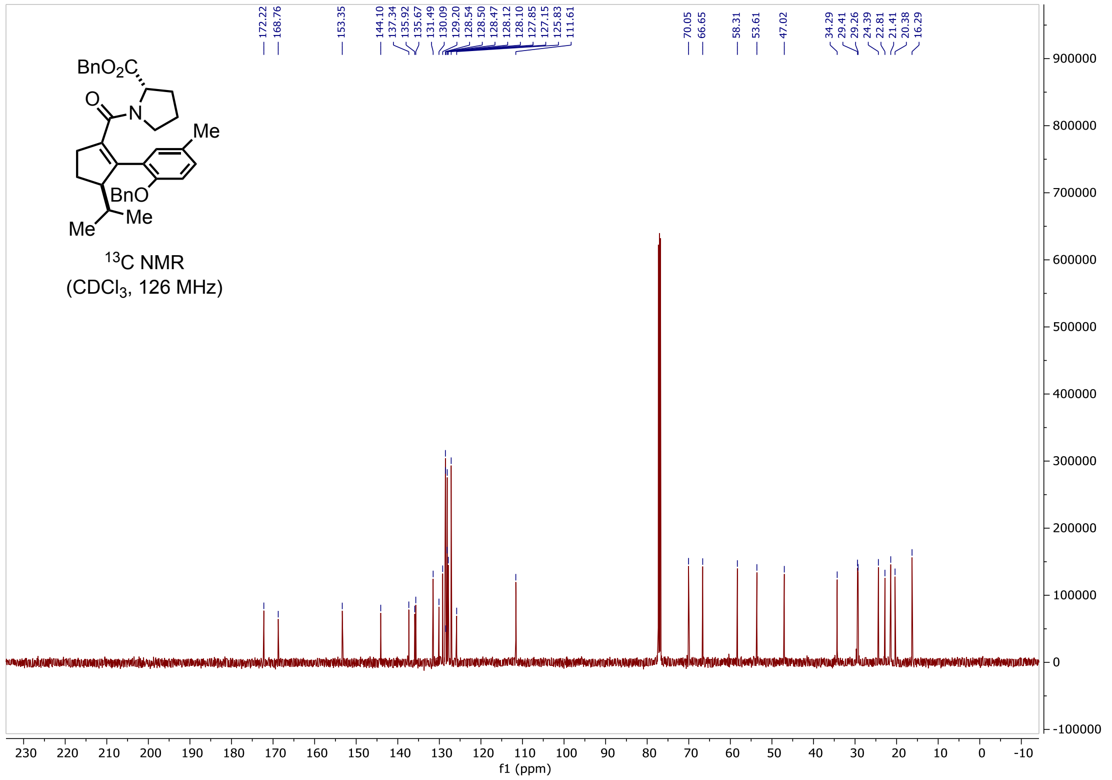
BnO$_2$C
O
N
Me
BnO
Me
Me
^{13}C NMR
(CDCl$_3$, 126 MHz)
172.22
168.76
153.35
144.10
137.34
135.92
135.67
131.49
130.09
129.20
128.54
128.50
128.47
128.12
128.10
127.85
127.15
125.83
111.61
70.05
66.65
58.31
53.61
47.02
34.29
29.41
29.26
24.39
22.81
21.41
20.38
16.29
f1 (ppm)

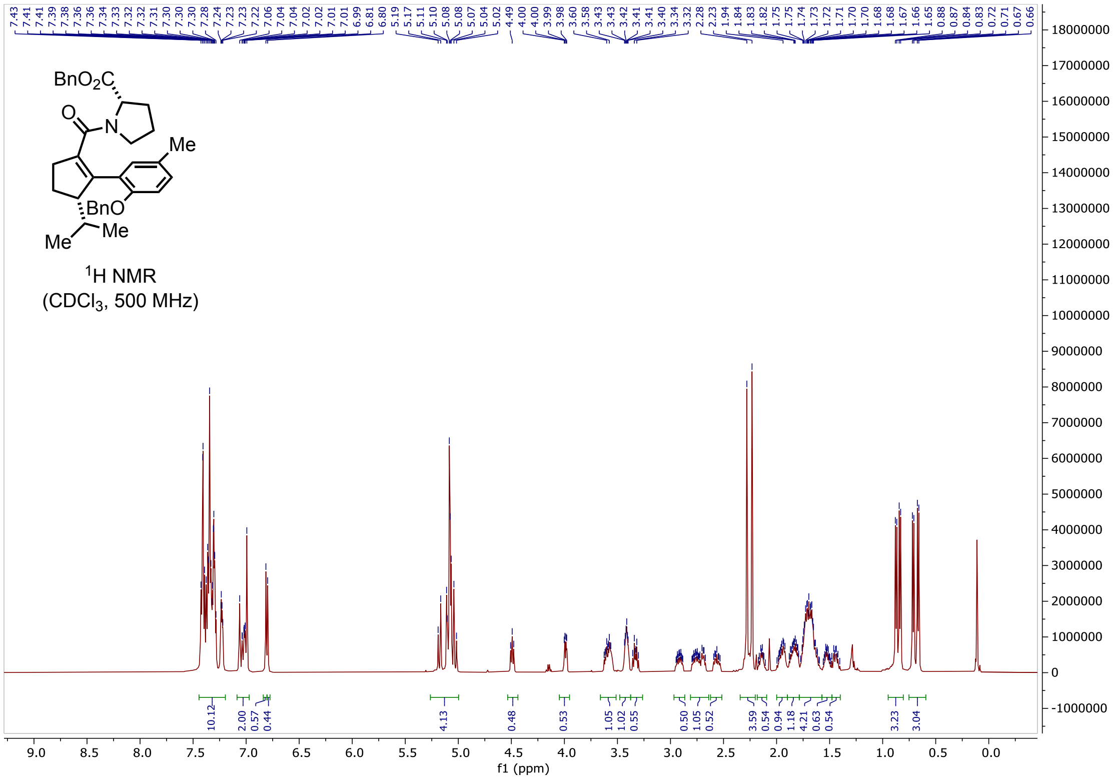
7.43 7.41 7.39 7.38 7.36 7.36 7.34 7.33 7.32 7.32 7.31 7.30 7.30 7.30 7.28 7.24 7.23 7.23 7.22 7.06 7.04 7.04 7.02 7.02 7.01 7.01 6.99 6.81 6.80 5.19 5.17 5.11 5.10 5.08 5.08 5.07 5.04 5.02 4.49 4.00 3.99 3.98 3.60 3.58 3.43 3.43 3.42 3.41 3.41 3.40 3.34 3.32 2.28 2.23 1.94 1.84 1.83 1.82 1.75 1.75 1.74 1.73 1.72 1.71 1.70 1.70 1.68 1.68 1.67 1.66 1.65 0.88 0.87 0.84 0.83 0.72 0.71 0.67 0.66
BnO2C
O
N
Me
BnO
Me
Me
1H NMR
(CDCl3, 500 MHz)
10.12 2.00 0.57 0.44 4.13 0.48 0.53 1.05 1.02 0.55 0.50 1.05 0.52 3.59 0.54 0.94 1.18 4.21 0.63 0.54 3.23 3.04
f1 (ppm)

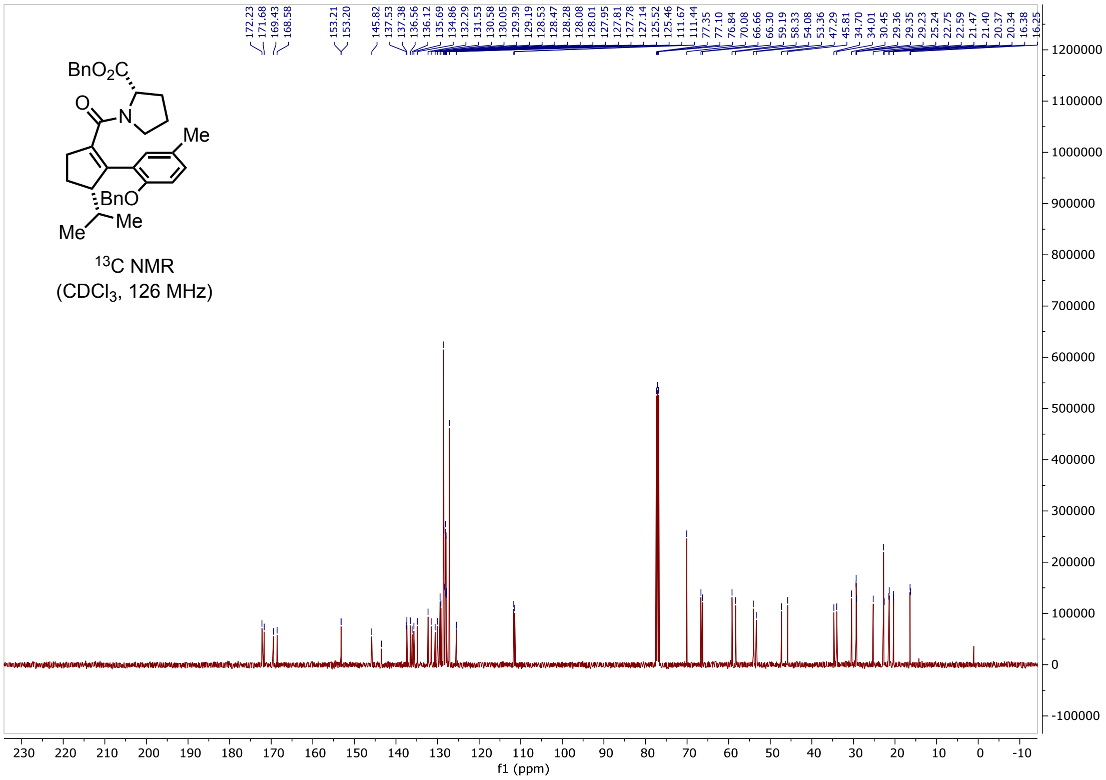
172.23 171.68 169.43 168.58 153.21 153.20 145.82 137.53 137.38 136.56 136.12 135.69 134.86 132.29 131.53 130.58 130.05 129.39 129.19 128.53 128.47 128.28 128.08 128.01 127.95 127.81 127.78 127.14 125.52 125.46 111.67 111.44 77.35 77.10 76.84 70.08 66.66 66.30 59.19 58.33 54.08 53.36 47.29 45.81 34.70 34.01 30.45 29.36 29.35 29.23 25.24 22.75 22.59 21.47 21.40 20.37 20.34 16.38 16.25
BnO2C
O
N
Me
BnO
Me
Me
13C NMR
(CDCl3, 126 MHz)
f1 (ppm)
